# Rh(iii)-Catalyzed [5 + 1] annulation of 2-alkenylanilides and 2-alkenylphenols with allenyl acetates†

**DOI:** 10.1039/d1sc06097j

**Published:** 2022-01-19

**Authors:** Anurag Singh, Rahul K. Shukla, Chandra M. R. Volla

**Affiliations:** Department of Chemistry, Indian Institute of Technology Bombay Powai Mumbai-400076 India Chandra.volla@chem.iitb.ac.in

## Abstract

Herein, we report a mild and highly regioselective Rh(iii)-catalyzed non-oxidative [5 + 1] vinylic C–H annulation of 2-alkenylanilides with allenyl acetates, which has been elusive so far. The reaction proceeds *via* vinylic C–H activation, regioselective 2,3-migratory insertion, β-oxy elimination followed by nucleophilic cyclization to get direct access to 1,2-dihydroquinoline derivatives. The strategy was also successfully extended to C–H activation of 2-alkenylphenols for constructing chromene derivatives. In the overall [5 + 1] annulation, the allene serves as a one carbon unit. The acetate group on the allene is found to be crucial both for controlling the regio- and chemoselectivity of the reaction and also for facilitating β-oxy elimination. The methodology was scalable and also further extended towards late stage functionalization of various natural products.

## Abstract 2

A highly regioselective Rh(iii)-catalyzed non-oxidative [5 + 1] vinylic C–H annulation of 2-alkenylanilides and 2-alkenylphenols with allenyl acetates was described for accessing dihyroquinoline and chromene derivatives.

## Graphical abstract

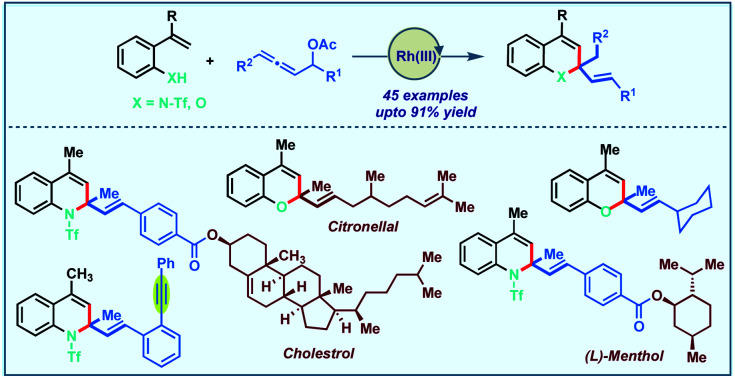

## Introduction

**Scheme 1:**
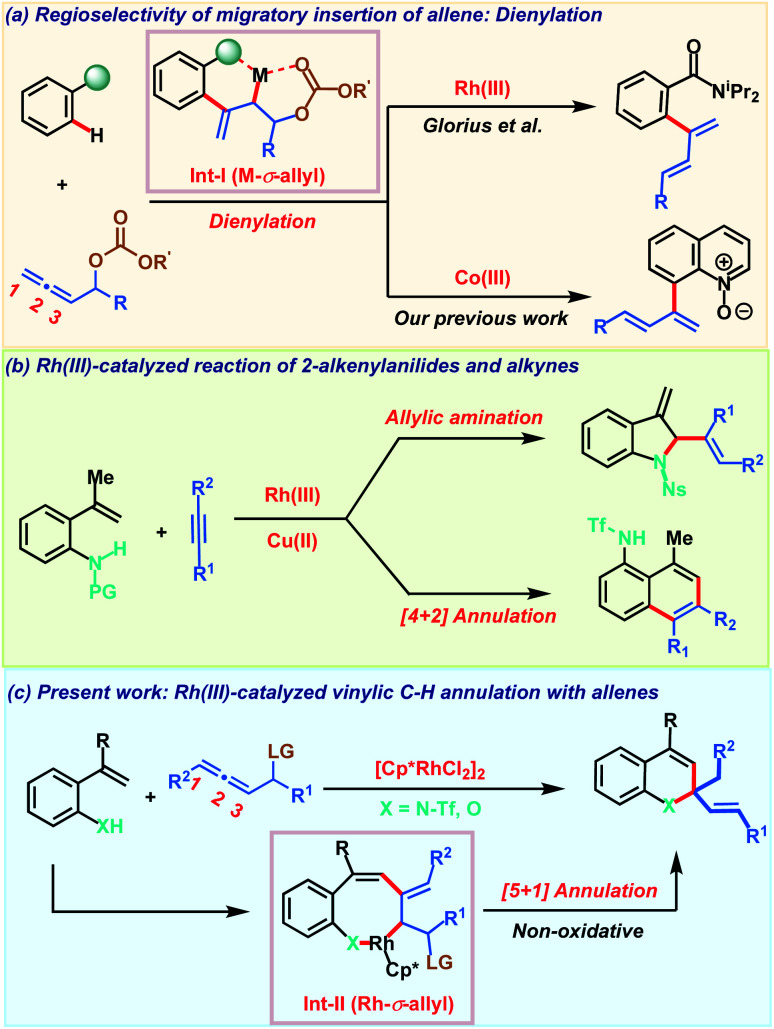
Overview of work.

Controlling regio- and chemoselectivity are the major challenging factors that impede the use of allenes in transition metal catalyzed C–H functionalizations in comparison to other π-systems like alkenes and alkynes.^1^ At the same time, deciphering the versatile reactivity of allenes in C–H annulations has great potential to generate molecular complexity rapidly.^2^ Addressing these inherent selectivity issues, recent years have witnessed significant advancements in unravelling the unique reactivities of allenes in C–H activation reactions with a variety of transition metals.^3,4^ The regioselectivity of migratory insertion of an allene with organometallic species R–M is influenced by the nature of the metal M and also by the steric and electronic attributes of the substituents present on the allene. In addition to these factors, we recently noticed that the heteroatom at the *α*-position of the allene also plays a key role in governing the insertion pattern of allenes.^5^ Taking advantage of this, Glorius and our group demonstrated C–H dienylation reactions of benzamides and quinoline-*N*-oxides employing allenyl carbinol carbonates (Scheme 1a). These dienylations proceed *via* regioselective 2,3-migratory insertion of the allene with an organometallic intermediate resulting in M-*σ*-allyl intermediate Int-I. The presence of oxygen induces the 2,3-migratory insertion selectively as the coordination interaction offers extra stability to the metallacycle and at the same time, also facilitates key β-oxy elimination. Allenes having a leaving group at the *α*-position were also exploited by Ackermann and co-workers in C–H activation with earth abundant and inexpensive iron salts involving an unprecedented 1,4-iron migration.^6^ Despite these three reports on allenyl carbinol carbonates, these allenes have been elusive so far in transition metal catalyzed C–H annulation reactions to access heterocycles.

On the other hand, transition metal catalyzed C–H functionalization^7^ of alkenyl C–H bonds is more appealing yet challenging as compared to aryl or alkyl C–H bond activation.^8^ Strong π-coordinating ability, low rotational barrier and sensitivity towards the steric hindrance on the alkene moiety make alkenyl C–H functionalization more arduous with transition metal catalysts. Key developments in this direction employing α,β-unsaturated oximes, acids, amides and enones for the construction of carbo- and heterocycles were achieved.^9^ These transformations typically proceed *via* C–H activation, insertion of a π-component followed by reductive elimination to deliver the five or six membered cyclic derivative *via* formal [3 + 2] or [4 + 2] annulation respectively. In contrast, Gulius and co-workers demonstrated oxidative annulations of *N*-protected 2-alkenylanilides with alkynes to furnish indolines and naphthalene derivatives (Scheme 1b).^10^ Despite their versatile reactivity, 2-alkenylanilides have not been studied in metal catalyzed C–H activation with allenes. Our continuous interest in allenes inspired us to explore their reactivity with 2-alkenylanilides under Rh(iii)-catalysis. Based on our previous report, we envisaged that diene formed after *β*-oxy elimination from the Rh-σ-allyl intermediate Int-II can be trapped by the nucleophilic heteroatom (NHTf or OH) for constructing valuable heterocycles. Furthermore, most of the alkenyl C–H annulations are oxidative processes and so require expensive metal oxidants based on silver or copper. So, they generate stoichiometric amounts of by-products decreasing the overall efficiency of the transformation. Consequently, development of non-oxidative processes bypassing the toxic metal-oxidants are desirable for achieving sustainable alkenyl C–H annulations. Addressing all these aspects associated with allenes and alkenyl C–H annulation, herein we demonstrate a robust Rh(iii)-catalyzed C–H non-oxidative [5 + 1] annulation of 2-alkenylanilides or 2-alkenylphenols with allenyl acetates to access 1,2-dihydroquinoline or chromene derivatives (Scheme 1c).

**Scheme 2:**
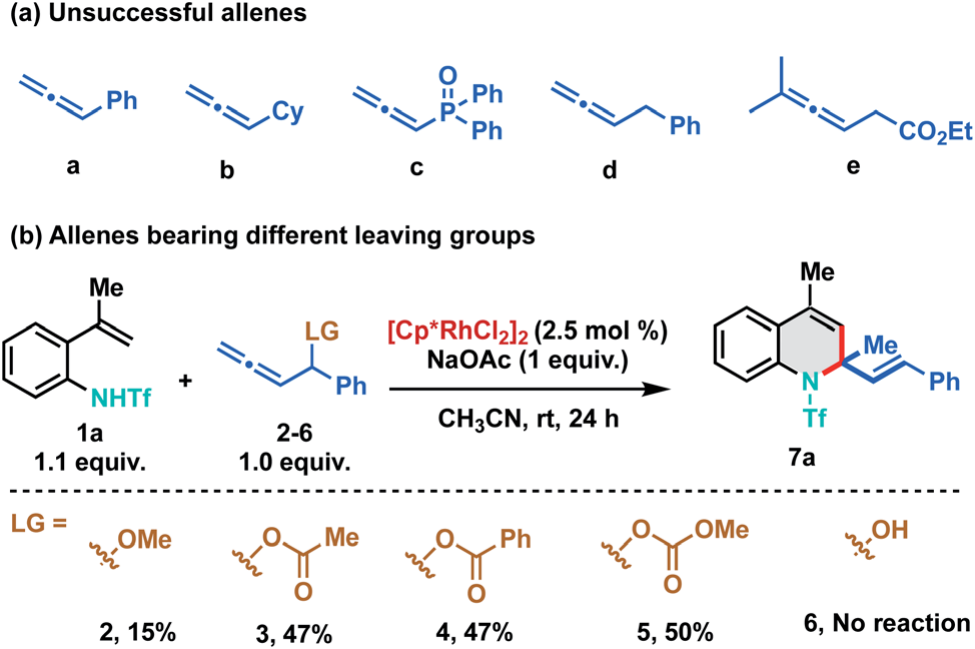
(a) Screening of various allenes. (b) Optimization of leaving groups.

As stereo-electronic attributes of allene-substituents play a key role in controlling their reactivity and selectivity of carbometalation, we commenced our initial investigation by studying the reaction of *N*-triflyl protected 2-alkenylanilide 1a with differently substituted allenes under Rh(iii)-catalysis (Scheme 2). No reaction was observed when 1a was treated with phenyl allene (a) using catalytic amounts of [Cp*RhCl_2_]_2_. Further studies with other allenes like cyclohexyl allene (b), phosphinyl allene (c), benzyl allene (d) and allenyl ester (e) were also found to be futile, as no clean reaction was observed using these allenes (Scheme 2a). At this juncture, we speculated that an allene bearing a leaving group at the *α*-position would promote the carbometalation as the heteroatom can facilitate migratory insertion by coordinating with the rhodium complex. We anticipated that the heteroatom would also induce migratory insertion with the C2–C3 double bond in a regioselective manner. Consequently, we tested phenyl allenyl methoxy ether 2 with 1a and to our delight, the desired [5 + 1] annulation product 7a was observed albeit in 15% yield. To facilitate the β-oxy elimination from the Rh-σ-allyl complex, we screened various leaving groups on the allene and as expected a significant increase in yield (47%) was observed with acetate (3) and benzoate (4) derivatives. Furthermore, allenyl carbinol carbonate (5) was proved to be most favorable for the transformation as it delivered the desired 1,2-dihydroquinoline in 50% yield. No dehydrative annulation was observed when phenyl allenyl alcohol (6) was employed.

To achieve further enhancement in yield, we carried out rigorous optimization studies by varying different reaction parameters and arrived at the following reaction conditions: 1.1 equiv. of 1a and 1.0 equiv. of 3a in the presence of 2.5 mol% of [Cp*RhCl_2_]_2_, and 30 mol% of NaOAc in DCE at room temperature to afford 7a in an excellent yield of 91% (isolated yield of 89%) (Table 1, entry 1). Both the Rh(iii)-catalyst and NaOAc were essential for the transformation, as the reaction did not proceed in the absence of the Rh(iii) catalyst and only 10% of 7a was observed without NaOAc (entries 2 and 3). Other additives like Cu(OAc)_2_ and AgOAc did not show any significant effect on the reaction (entries 4 and 5). Silver salts like AgSbF_6_ and AgBF_4_, having non-coordinating counter anions, which are known to enhance the reactivity of the Rh(iii)-catalyst, led to 7a only in 70% and 68% yield, respectively (entries 6 and 7). Screening of other solvents instead of DCE led to 7a in lower yields (entry 8). Increasing the temperature to 70 °C had a significant impact as the annulation was completed within 30 min providing 7a in 87% yield (entry 9). No 7a was detected in the crude NMR spectrum when other metal catalysts like [Ru(*p*-cymene)Cl_2_]_2_, [Cp*Co(CO)I_2_] or [Cp*IrCl_2_]_2_ were employed instead of Rh(iii) (entries 10–12), indicating the unique selectivity of [Cp*RhCl_2_]_2_ for the transformation.

**Table 1:** Optimization of reaction conditions^a^

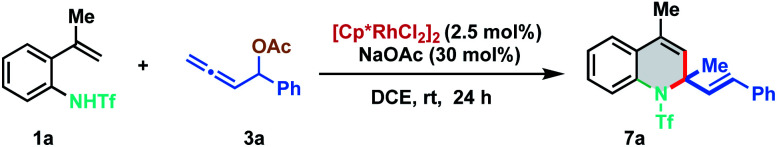		
Entry	Variation from standard conditions	Yields^b^ (%)
1	None	91(89)^c^
2	Without [Cp*RhCl_2_]_2_	—
3	Without NaOAc	10
4^d^	Cu(OAc)_2_ instead of NaOAc	73
5^d^	AgOAc instead of NaOAc	68
6^e^	AgSbF_6_ instead of NaOAc	70
7^e^	AgBF_4_ instead of NaOAc	68
8	Toluene, MeOH, 1,4-dioxane, DMF, CH_3_CN instead of DCE	<75
9	*T* = 70 °C, 30 min	87
10	[Ru(*p*-cymene)Cl_2_]_2_ instead of Rh(iii)	—
11	[Cp*Co(CO)I_2_] instead of Rh(iii)	—
12	[Cp*IrCl_2_]_2_ instead of Rh(iii)	—

a Reaction conditions: 1a (0.2 mmol), 3a (0.18 mmol), [Cp*RhCl_2_]_2_ (2.5 mol%), and NaOAc (30 mol%) in 1 mL DCE at room temperature for 24 h.

b Yield is calculated based on ^1^H NMR of the crude reaction mixture using 1,3,5-trimethoxybenzene as the internal standard.

c Yield in parentheses refers to isolated yield.

d Additive in 1 equiv.

e Additive in 10 mol%.

**Scheme 3:**
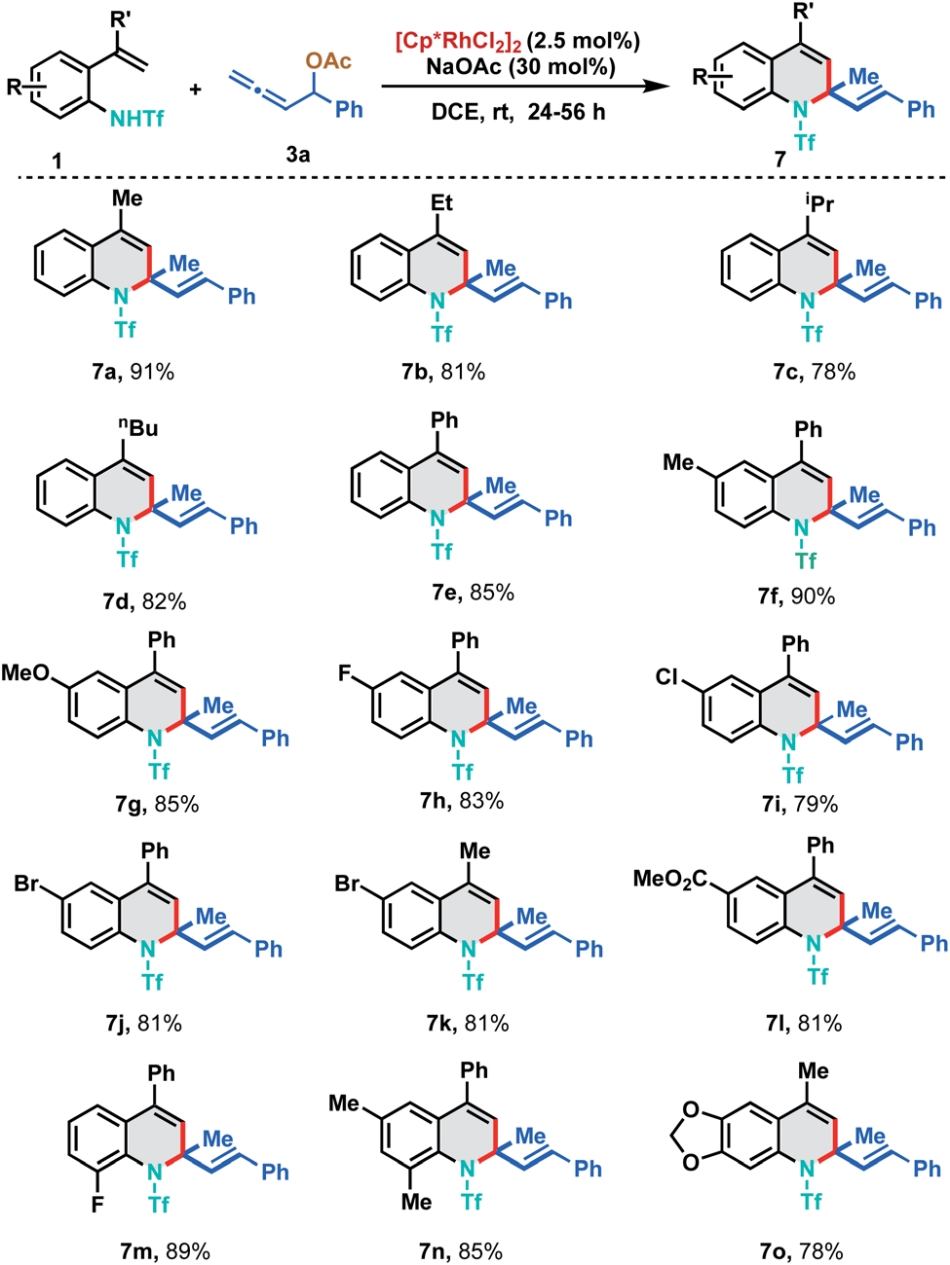
Substrate scope of 2-alkenyl anilides.

With the optimized reaction conditions in hand, we investigated the generality of the developed protocol with diversely substituted 2-alkenylanilides 1 employing allene 3a. As depicted in Scheme 3, a wide range of desired 1,2-dihydroquinoline derivatives were obtained in good to excellent yields (7a–7o). Regarding the alkenyl substituent fragment, ethyl, i-propyl, *n*-butyl and phenyl derivatives were found to be suitable substrates to afford the corresponding quinolines 7b–7e in 78–85% yields. It is worth mentioning that simple vinyl substituted aniline (R′=H) and trisubstituted alkenylanilides did not show any reactivity for the annulation. In order to explore the functional group compatibility, differently substituted 2-alkenylanilides were tested for [5 + 1] annulation. Electron donating groups like methyl and methoxy fared well under the standard reaction conditions furnishing the corresponding 1,2-DHQ products 7f and 7g in 90% and 85% yield respectively. 2-Alkenylanilides bearing halogen substituents like fluoro, chloro and bromo were also probed to isolate the corresponding annulated products in good to excellent yields (7h–7k, 79–83%). Electron withdrawing group like ester resulted in 7l with 81% yield. Further, *ortho*-substituted as well as disubstituted alkenylanilides delivered the corresponding dihydroquinoline derivatives in good to excellent yields under the optimized reaction conditions (7m–7o, 78–89%).

**Scheme 4:**
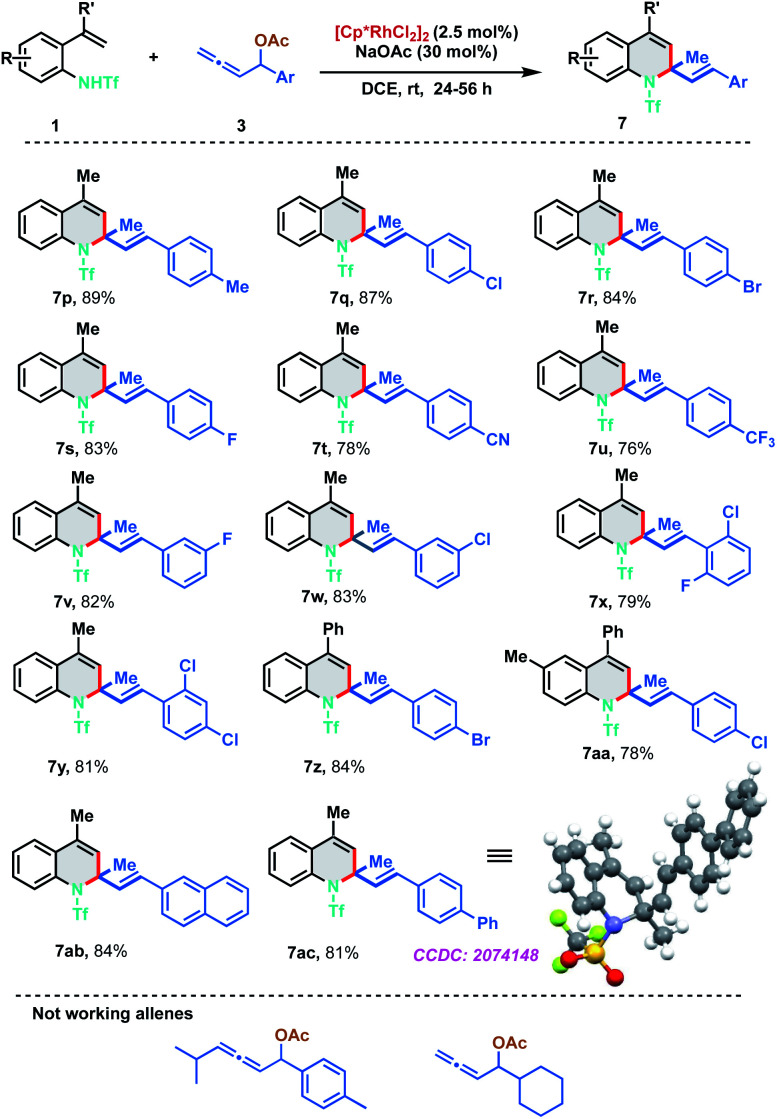
Substrate scope of allenyl acetates.

To further evaluate the scope of the developed protocol, different substituents on the aryl ring of allenyl acetate were probed with 2-alkenylanilide 1a (Scheme 4). Experimental outcomes indicated that both electron-donating and electron-withdrawing substituents at *ortho*-, *meta*- and *para*-positions of aryl rings were well tolerable for the [5 + 1] annulation to furnish the dihydroquinoline derivatives 7p–7aa in good to excellent yields (76–89%). Electron withdrawing groups like CN and CF_3_ were found to be slightly less efficient than other substituents. Highly conjugated 2-naphthyl and biphenyl derivatives were also found to be amenable substrates for the protocol and delivered the corresponding 7ab and 7ac in 84% and 81% yield respectively. The structure of the [5 + 1]-annulation was unambiguously confirmed by the single-crystal X-ray diffraction analysis of 7ac. Our efforts to expand the protocol with 1,3-disubstituted allenes and aliphatic allenes met with failure even at high temperatures and long reaction times.

**Scheme 5:**
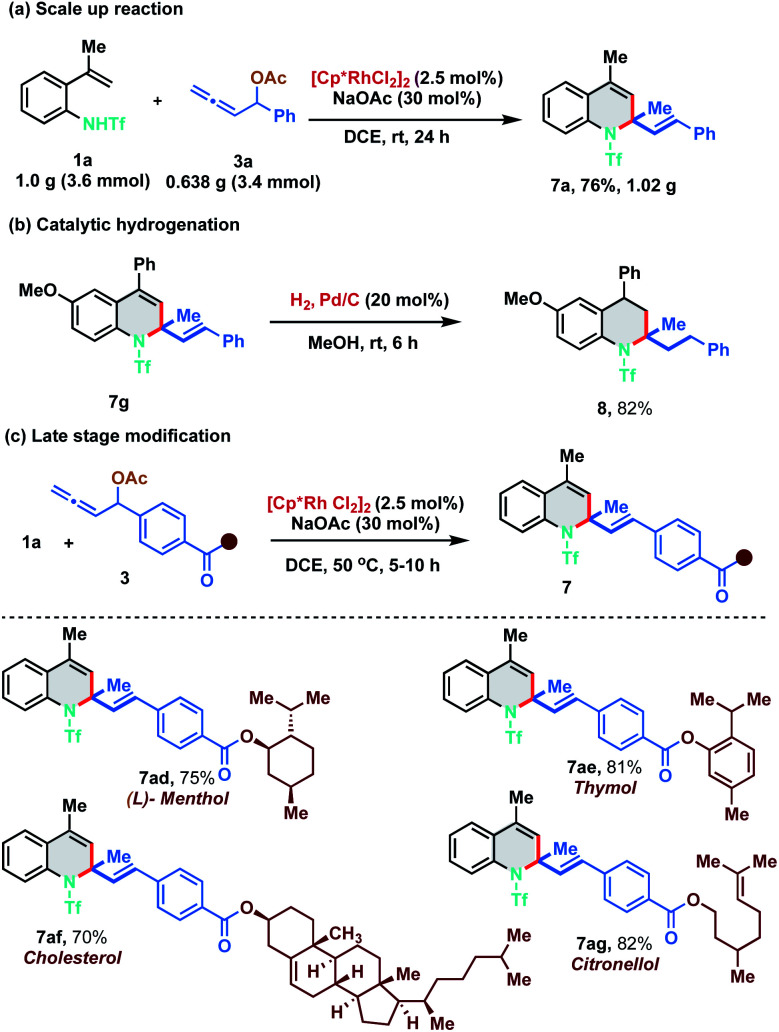
Scale up reaction, functionalization and diversification of natural product derivatives.

As our developed protocol provides an atom economical entry for synthesizing an array of 1,2-dihydroquinolines under mild reaction conditions, we performed a scale up reaction to probe its synthetic utility. Reaction of 1.0 g of 1a (3.6 mmol) with 0.638 g of 3a (3.4 mmol) under the optimized reaction conditions furnished 1.02 g of 7a in 76% yield (Scheme 5a). Furthermore, 1,2-DHQ 7g was readily converted to 1,2,3,4-tetrahydroquinoline 8 (82% yield) under palladium-catalyzed hydrogenation conditions (Scheme 5b). The 1,2-DHQ motif is a privileged heterocycle that is present in many biologically active molecules like antioxidants, antibacterials, natural products and antijuvenile hormone insecticides.^11^ Direct installation of the 1,2-DHQ moiety represents a unique strategy for accessing a new pool of functionalized analogues. Thus, to test the practicality of our developed method in late stage functionalization, allenyl acetates derived from (l)-menthol, thymol, cholesterol and citronellol were synthesized and subjected to the optimized reaction conditions with 1a at a slightly higher temperature (50 °C). To our delight, [5 + 1] annulation proceeded smoothly to afford 7ad, 7ae, 7af, and 7ag in 75%, 81%, 70% and 82% yield respectively (Scheme 5c).

**Scheme 6:**
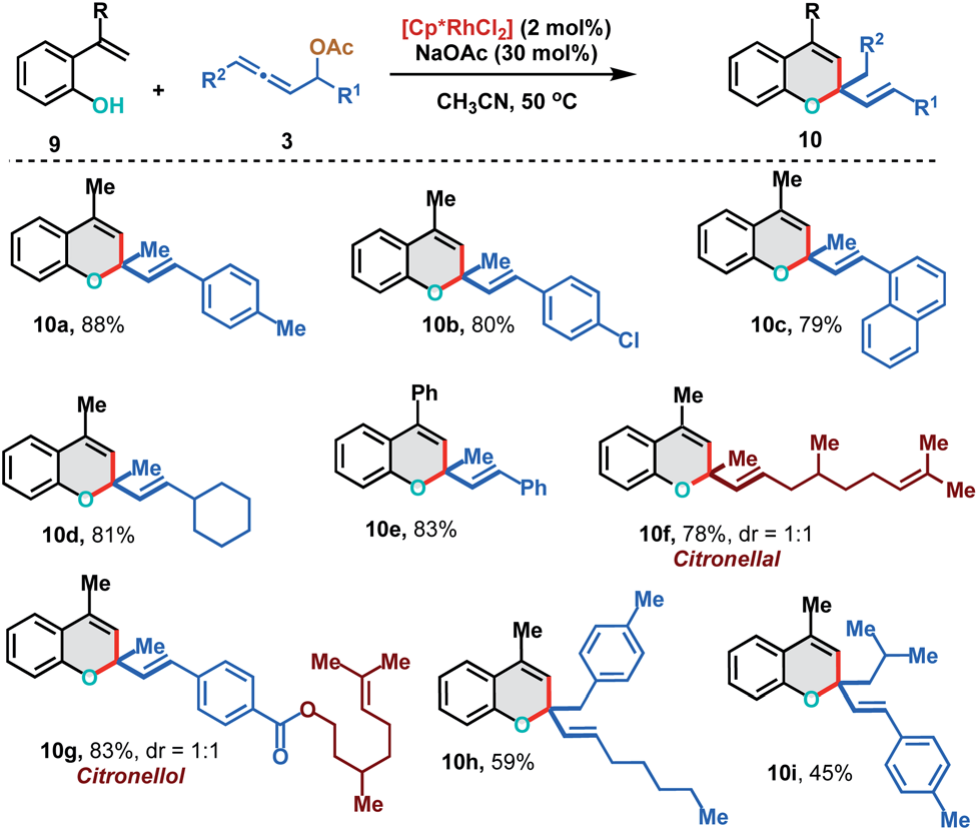
[5 + 1] annulation of 2-alkenylphenols with allenyl acetates.

We further extended the [5 + 1] annulation strategy by coupling 2-alkenylphenols 9 with allenyl acetates 3 to access chromene derivatives 10 in the presence of 2 mol% of the Rh(iii)-catalyst under slightly modified reaction conditions (Scheme 6). 2-(Prop-1-en-2-yl)phenol 9a reacted smoothly with *p*-Me and *p*-Cl substituted allenyl acetates to deliver the chromenes 10a and 10b in excellent yields (88% and 80%). Interestingly, allene having an aliphatic substituent, which failed to react with 2-alkenylanilide, displayed facile reactivity with 2-alkenylphenol to provide 10d in 81% yield. Allenyl acetate derived from natural products like citronellal and citronellol could also be effectively applied to access the corresponding chromenes 10f and 10g in 78% and 83% yield respectively. Further, 1,3-disubstituted allenes also fared well under the reaction conditions to furnish 10h and 10i in moderate yields (59% and 45%).

**Scheme 7:**
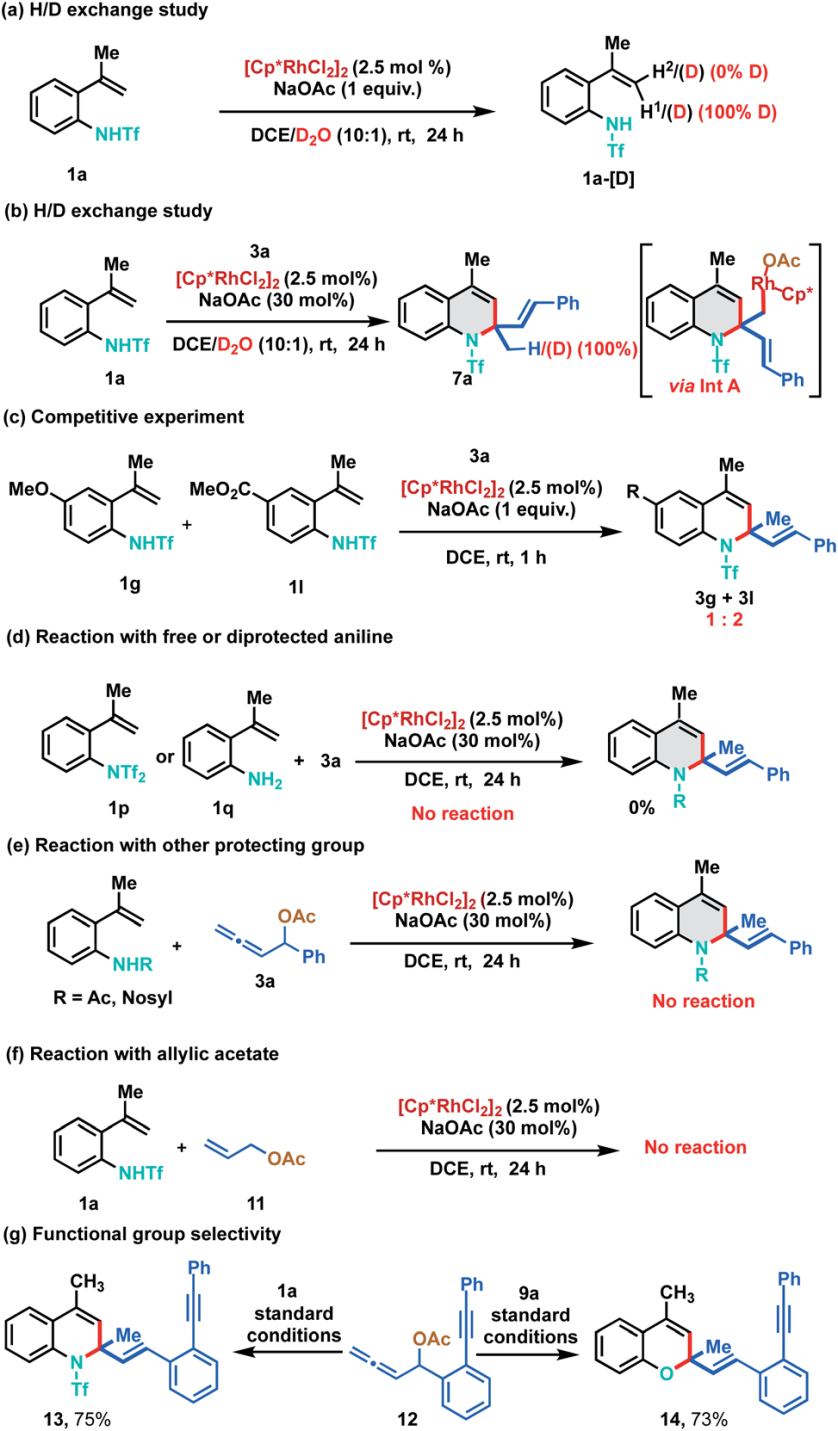
Preliminary mechanistic studies.

In order to gain understanding on the reaction mechanism, different control and deuterium exchange experiments were carried out (Scheme 7). A deuterium exchange experiment of 1a in the absence of allenyl acetate, using a 10 : 1 mixture of DCE and D_2_O indicated 100% deuterium incorporation of alkenyl C–H (H^1^) that is *cis* to the phenyl ring. However, no deuterium incorporation was detected on the *trans* alkenyl C–H (H^2^) of 1a (Scheme 7a). These observations reveal that C–H bond cleavage is reversible in nature and indicate that C–H activation might be proceeding *via* the typical concerted metalation–deprotonation (CMD) mechanism rather than intramolecular attack of the conjugated alkene to the electrophilic Rh(iii) complex. Further, similar studies in the presence of allenyl acetate 3a resulted in dihydroquinoline 7a having 100% deuterium incorporation, suggesting that the reaction might be proceeding *via* the protonation of the organorhodium intermediate (Int A) (Scheme 7b, *vide infra*). An intermolecular competitive experiment with 4-methoxy and 4-ester substituted alkenylanilides 1g and 1l with allene 3a was conducted, which indicated that the annulation proceeds preferentially with electron deficient alkenylanilides, throwing light on the C–H activation step (Scheme 7c). Under the standard reaction conditions, a control experiment between di-protected aniline 1p and 3a did not provide any product, suggesting that initial N–Rh bond formation ensues *via* the abstraction of the acidic N–H proton. Similarly, 2-alkenyl aniline 1q also failed to furnish any product indicating that the free amino group is not compatible in this catalytic cycle (Scheme 7d). In another control study, other protecting groups like acetyl and nosyl were tested instead of triflyl under the standard reaction conditions. However, the corresponding products were not observed implying the crucial role of the triflyl group for the success of the transformation (Scheme 7e). Finally, the use of allyl acetate 11 instead of allenyl acetate 3 was probed in the reaction with 1a and the reaction failed to provide any product (Scheme 7f). To examine the functional group selectivity of the annulation reaction, we synthesized substrate 12 having both allene and alkyne functionalities. Interestingly, cyclization occurred selectively with the allene when 1a or 9a was treated with 12 under standard reaction conditions to afford 13 (75%) or 14 (73%) respectively leaving the alkyne intact (Scheme 7g).

**Scheme 8:**
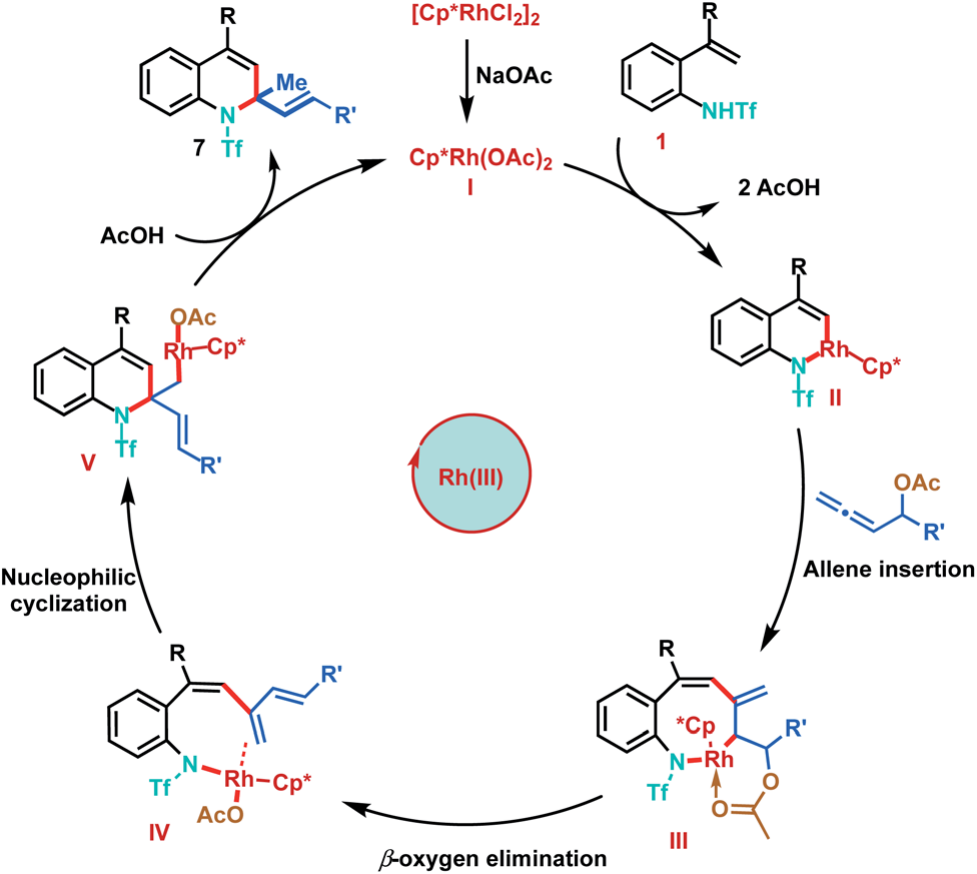
Proposed mechanism.

Based on the mechanistic studies and previous literature precedence, the plausible mechanism for the Rh(iii)-catalysed [5 + 1]-annulation is proposed as shown in Scheme 8. The catalytic cycle is initiated by the generation of active Rh(iii) complex I in the presence of NaOAc. Reaction of I with 2-alkenylanilide 1 proceeds *via* anion-exchange followed by vinyl C–H activation to form six-membered rhodacycle II, through a typical concerted metalation–deprotonation step (CMD). Furthermore, co-ordination of allene 3 followed by regioselective 2,3-migratory insertion with intermedium II gives the eight-membered Rh(iii) intermediate III. Subsequent β-oxygen elimination from species III gives the diene intermediate IV, which undergoes intramolecular nucleophilic addition of the nitrogen–rhodium bond resulting in [5 + 1]-annulation. Finally, protonation of Int V provides the desired 1,2-dihydroquinolines 7 and regenerates the active Rh(iii) catalyst.

## Conclusions

In conclusion, we developed an unprecedented, robust Rh(iii)-catalyzed C–H functionalization of 2-alkenylanilides with allenyl acetates for the construction of 1,2-DHQ derivatives. Essentially, it is the first report where allenes having leaving groups were used as coupling partners in Rh(iii)-catalyzed C–H annulation reactions. The developed protocol displayed a broad substrate scope with good to excellent yields, against a variety of diversely substituted 2-alkenylanilides and allenyl acetates at room temperature. A mechanistic study suggests that the reaction proceeds *via* β-oxygen elimination leading to the formation of a butadiene intermediate, which gets trapped by the nucleophilic directing group, NHTf to furnish the [5 + 1] annulation products. The annulation was also expanded to 2-alkenylphenols to get direct access to chromene derivatives. Further, synthetic utility was justified by carrying out a gram scale reaction and functionalization by readily converting 1,2-DHQ to 1,2,3,4-tetrahydroquinolines. To expand the generality of the protocol, diversification of natural products like (l)-menthol, thymol, citronellol and cholesterol was also carried out.

## Data availability

Detailed synthetic procedures, complete characterization data for all new compounds can be found in the ESI.†

## Author contributions

A. S. and R. K. S. designed and conducted all experiments and characterized the novel compounds. A. S. and C. M. R. V. wrote the manuscript. C. M. R. V. directed the research.

## Conflicts of interest

There are no conflicts to declare.

## Supplementary Material

SC-013-D1SC06097J-s001

SC-013-D1SC06097J-s002
