# Supplementary material for: Rh(iii)-Catalyzed [5 + 1] annulation of 2-alkenylanilides and 2-alkenylphenols with allenyl acetates
Source: Chem Sci. 2022 Jan 19;13(7):2043–9. doi: 10.1039/d1sc06097j (PMC8848808; doi:10.1039/d1sc06097j)
Supplement: SC-013-D1SC06097J-s001 [file SC-013-D1SC06097J-s001.pdf]

Electronic Supplementary Material (ESI) for Chemical Science.
This journal is © The Royal Society of Chemistry 2022

Rh(III)-Catalyzed [5+1] Annulation of 2-Alkenylanilides and 2-Alkenylphenols with Allenyl Acetates

Anurag Singh, Rahul K. Shukla, Chandra M. R. Volla.

Department of Chemistry, Indian Institute of Technology Bombay, Powai, Mumbai-400076, India
Chandra.volla@chem.iitb.ac.in

Supporting Information

Contents :

1. General methods S2
2. Preparation of 2-alkeny anilides and allenyl acetate S3
3. Optimization S4-S5
4. General procedure **A**: Synthesis of 1,2dihydroquinolin derivatives S5
5. Spectroscopic data S6-S23
6. Functionalization S24
7. Mechanistic studies S25-S28
8. References S28
9. NMR spectra S29

1. General information:

All reactions were performed in a 15 mL reaction tube. Unless otherwise noted, staring materials, reagents and solvents were purchased from common commercial sources and used without further purification. Starting materials were prepared according to the existing literature procedures. ^{1}H NMR spectra were recorded at 400 MHz and 500 MHz using TMS as internal standard. ^{13}C NMR spectra were recorded at 100 MHz and 125 MHz using TMS as internal standard. High resolution mass measurements were carried out using Micromass Q-ToF ESI instrument using direct inlet mode. Analytical thin-layer chromatography (TLC) was performed on pre-coated 0.2 mm thick Merck 60 F_{245} silica plates and various combinations of ethyl acetate and petroleum ether were used as eluent. Visualization of spots of allene and final product was accomplished by subjecting to $KMnO_4$ stain. All compounds were purified using silica gel (100-200 mesh) column chromatography and gave spectroscopic data consistent with being ≥95% the assigned structure.

2. General procedure for the synthesis of 2-alkenyl anilides.[1,2]

Step 1:

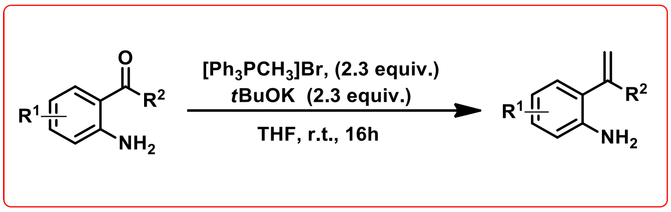

To a solution of methyltriphenylphosphonium bromide (2.04 g, 2.3 equiv) and potassium tert-butoxide (0.699 g, 2.3 equiv) in THF (26 mL) under Ar atmosphere was added the 2-aminoacetophenone derivative (0.50 g, 4.09 mmol). The reaction was heated at 30 °C and stirred for 12 hours and then cooled to room temperature. Solvents were removed in vacuo and the resulting mixture was extracted with diethyl ether. The combined organic layers wer washed with brine, and dried over anhydrous sodium sulfate. Evaporation of the solvent followed by purification by flash chromatography on silica gel (hexanes:diethylether; 4:6) gave the 2-alkenyl aniline derivatives.

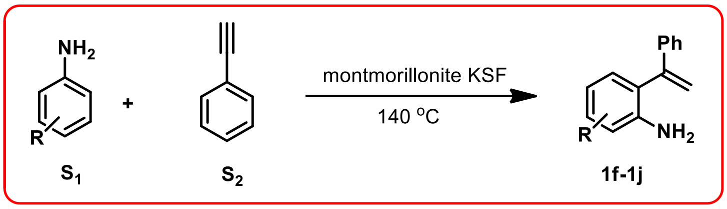

Aniline (5 mmol) A, phenylacetylene (0.51 g, 5 mmol) B and montraorillonite KSF S4 (0.51 g) are introduced in a round bottomed flask equipped with magnetic stirrer and a reflux condenser. The reaction mixture is heated at 140 °C for 5 hours and then cooled to room temperature. The products was dissolved with dichloromethane and filtered. Then the solvent were concentrated in vacuo and the crude was purified by column chromatography (silica gel, appropriate mixture of petroleum ether /ethyl acetate) to give corresponding 2-alkenyl aniline.

Step 2:

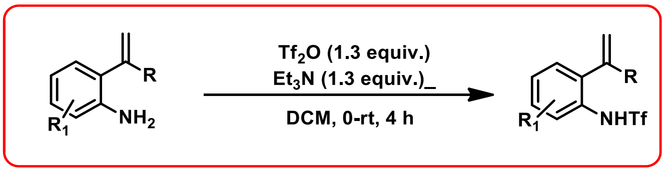

To a solution of o-isopropenylaniline (1 mL, 7.34 mmol) in dichloromethane (25 mL) under Ar atmosphere was added triethylamine (1.228 ml, 1.2 equiv) at °C. Then trifluoromethanesulfonic anhydride (1.489 ml, 1.2 equiv) was added dropwise. The reaction was stirred at 0 °C for 1.5 hours and quenched with saturated NH_4Cl aqueous solution. The resulting mixture was extracted with dichloromethane and dried over anhydrous sodium sulfate. Evaporation of the solvent followed by purification column flash chromatography on silica gel (hexanes:diethylether; 8:2) affording corresponding products.

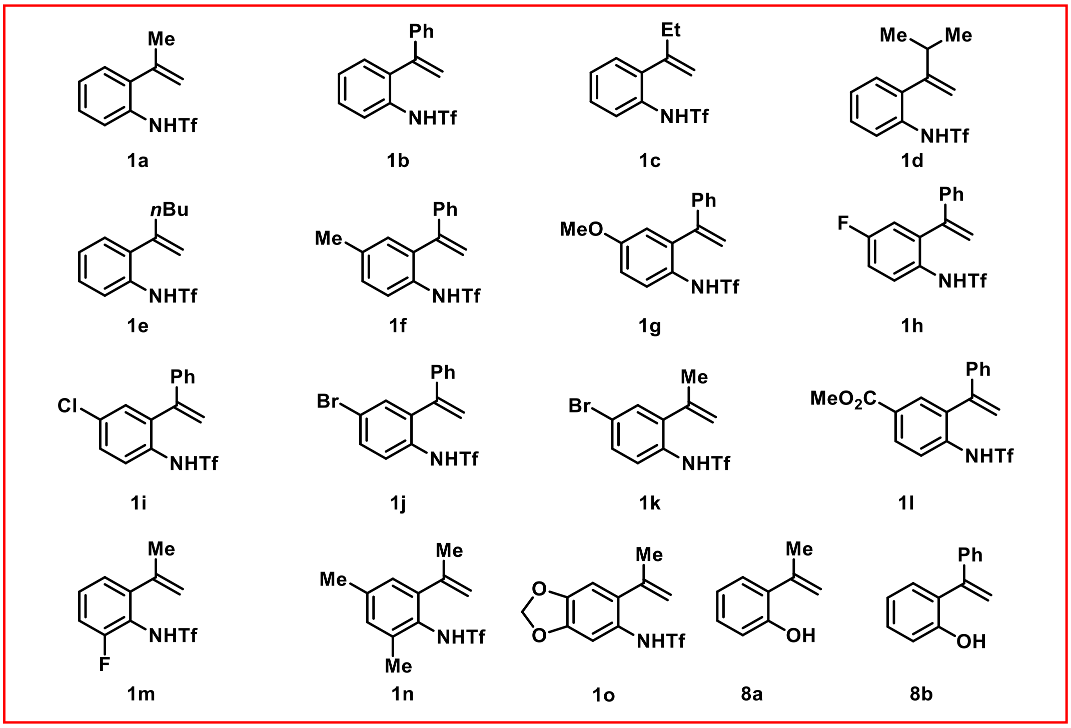

General procedure for the synthesis of Allenyl Acetate.[3,4]

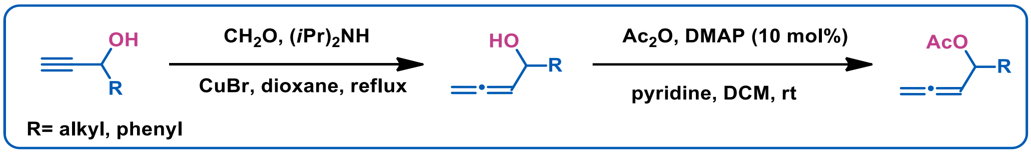

Step 1:

To a two-necked round bottom-flask equipped with a magnetic stir bar were added under argon propargylic alcohol (10 mmol), 15 mL dioxane, 0.72 g of cuprous bromide, 0.74 g of paraformaldehyde, and 1.85 g of diisopropylamine. The reaction mixture was refluxed for 2 h and then cooled to room temperature. To mixture was filtered through a Celite plug. The filtrate is diluted with water followed by diethyl ether and acidified with 6 N HCl to pH 2. The organic layer was separated and the aqueous phase was extracted with diethyl ether for additional two times. The organic phase was then washed with saturated $NaHCO_3$, brine and dried over $MgSO_4$. After filtration and evaporation under reduced pressure, the residue was subjected directly for the next step.

Step 2: To a round bottom-flask equipped with a magnetic stir bar were added under argon allenyl carbinol, DMAP (122 mg, 1.0 mmol, 0.2 equiv), pyridine (790 mg, 10 mmol, 2.0 equiv) and dichloromethane (0.3 M). The mixture was cooled to 0 °C and the chloro methyl formate (708.8 mg, 7.5 mmol, 1.5 equiv) was slowly added. The reaction was allowed to stir at room temperature until completion (typically 1 – 16 h). The mixture was diluted with dichloromethane and washed successively with 1 N HCl, saturated $NaHCO_3$, and brine. The organic phase was dried over $MgSO_4$, filtered and evaporated under reduced pressure. The residue was purified by flash column chromatography to yield the desired product.

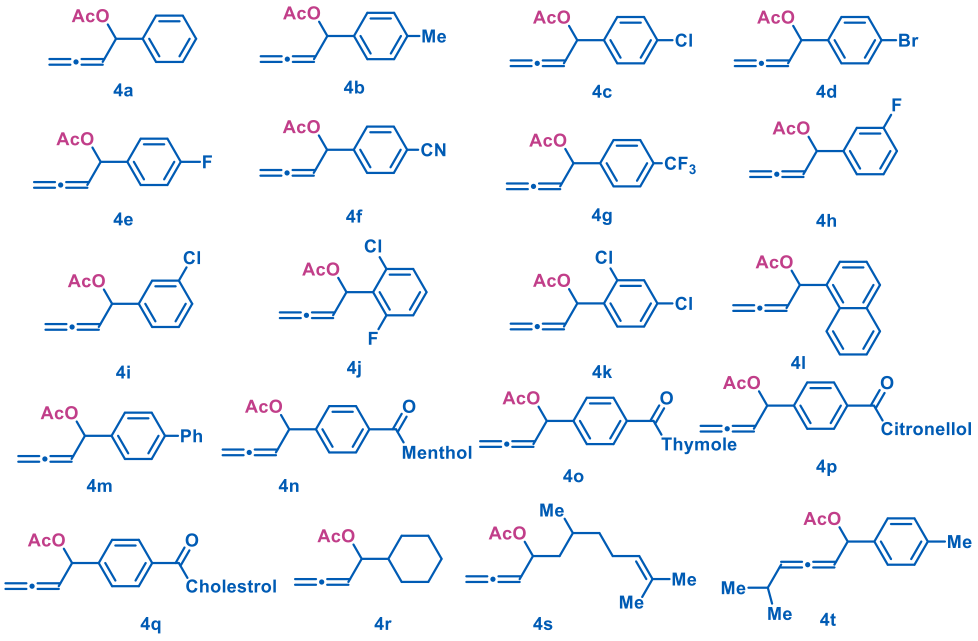

3. Optimization of reaction condition:

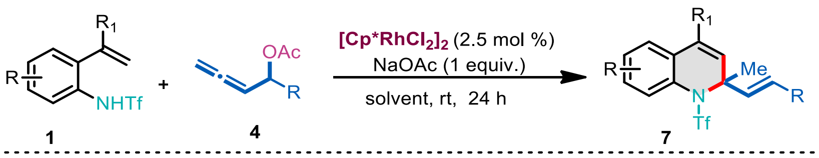

To achieve further enhancement in yield, different solvents were screened. DCE proved to be best solvent for developed protocol delivering the product **7a** in 93% yield, while other solvents like toluene, 1,4 dioxane, MeOH and DMF were found less efficient hence lowered yield were observed (entry 3-6). The presence of other additives like $Cu(OAc)_2$, CsOAc and AgOAc did not exhibit any significant improvement in reaction yield (entry 7-9). Surprisingly, silver salts like $AgSbF_6$ and $AgBF_4$, having non-coordinating counter anions which are known to enhance the reactivity of the Rh(III)-catalyst, gave the product **3a** only in 70% and 68%, respectively (entries 10 and 11). After carefully examination of optimization, we found that loading of NaOAc could be further lower down to 30 mol% in DCE solvent (entry 12). In the absence of NaOAc, diminished yield of **7a** (20%) was observed (entry 11). When reaction was also carried out at elevated temperature at 70 °C, formation of **7a** was noticed within 20 minute with 86% yield (entry 12). Other metal catalyst like $Pd(OAc)_2$, [Cp*Co(CO)I_2], [Cp*$IrCl_2$]$_2$ and [Ru(p-cymene)Cl2]2 were found completely ineffective when used instead of Rh(III) (entry 13). Absence of Rh catalyst did not furnish the cyclized product (entry 14).

entry	catalyst	solvent	base	yield (%)

**1**	$[Cp^*RhCl_2]_2$	CH_3CN	NaOAc	45 (42)[b]
**2**	$[Cp^*RhCl_2]_2$	DCE	NaOAc	93 (89)
**3**	$[Cp^*RhCl_2]_2$	Toluene	NaOAc	75
**4**	$[Cp^*RhCl_2]_2$	1,4-dioxane	NaOAc	25
**5**	$[Cp^*RhCl_2]_2$	MeOH	NaOAc	64
**6**	$[Cp^*RhCl_2]_2$	DMF	NaOAc	43
**7**	$[Cp^*RhCl_2]_2$	DCE	$Cu(OAc)_2$	73
**8**	$[Cp^*RhCl_2]_2$	DCE	CsOAc	68
**9**	$[Cp^*RhCl_2]_2$	DCE	AgOAc	68
**10**	$[Cp^*RhCl_2]_2$	DCE	$AgSbF_6$	70
**11**	$[Cp^*RhCl_2]_2$	DCE	$AgBF_4$	68
**12**	$[Cp^*RhCl_2]_2$	DCE	NaOAc	91 [c]
**13**	$[Cp^*RhCl_2]_2$	DCE	-	10
**14**	$[Cp^*RhCl_2]_2$	DCE	NaOAc	87[d]
**15**	[Ru(*p*-cymene)$Cl_2]_2$	DCE	NaOAc	-
**16**	$Pd(OAc)_2$	DCE	NaOAc	-
**17**	$[Cp^*IrCl_2]_2$	DCE	NaOAc	-
**18**	$[Cp^*Co(CO)I_2]_2$	DCE	NaOAc	-
**17**	-	DCE	NaOAc	-

[a] Reaction condition: **1a** (0.2 mmol), **2a** (0.18 mmol), solvent 2 ml, 36 h. [b] isolated yield. [c] Used 30 mol % NaOAc. [d] T= 70 °C.

3. General procedure for Rh(III) -catalyzed [5+1] annulation of *o*-alkenylanilides with allenic acetates.

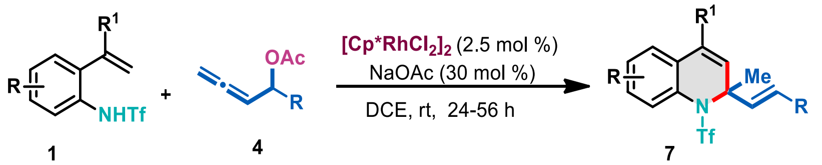

1 4 7

A sealed tube containing $[Cp^*RhCl_2]_2$ (2.5 mol %), NaOAc (30 mol %) was evacuated and purged with nitrogen gas three times. Then, *o*-alkenylanilides **1** (0.20 mmol) and allenic acetate **4** (0.18 mmol) in CH_3CN (2 ml) were added via syringe under nitrogen atmosphere and the reaction mixture was allowed to stir at r.t. for 24 h. Then, the mixture was diluted with CH_2Cl_2 (10 mL). The mixture was filtered through a celite pad and washed with CH_2Cl_2 (3 × 10 mL). The filtrate was concentrated under reduced

pressure. The residue was purified by silica gel column chromatography using hexane/ethyl acetate as eluent to afford the desired annulated pure product **7**.

4. Spectroscopic data:

(E)-2,4-dimethyl-2-styryl-1-((trifluoromethyl)sulfonyl)-1,2-dihydroquinoline

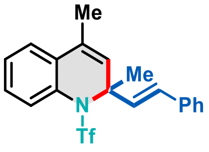

7a, 91%

64.4 mg, 91%, colorless liquid;

^{1}H NMR (500 MHz, $CDCl_3$) δ 7.61 – 7.53 (m, 1H), 7.35 (dd, *J* = 7.4, 1.9 Hz, 1H), 7.32 – 7.27 (m, 4H), 7.27 – 7.22 (m, 3H), 6.44 (d, *J* = 15.9 Hz, 1H), 6.17 (d, *J* = 15.9 Hz, 1H), 5.97 (s, 1H), 2.25 (d, *J* = 1.4 Hz, 3H), 1.88 (s, 3H).

^{13}C{^{1}H} NMR (125 MHz, $CDCl_3$) δ 136.16, 134.27, 132.87, 132.17, 131.27, 130.68, 129.61, 128.55, 128.06, 127.93, 127.89, 127.72, 126.59, 123.09, 120.45 (q, *J* = 325.9 Hz), 64.29, 26.56, 17.95.

HRMS (ESI-TOF) *m/z* $[M + Na]^+$ calcd for $C_{20}H_{18}F_3NNaO_2S$ 416.0903, found 416.0907.

(E)-4-ethyl-2-methyl-2-styryl-1-((trifluoromethyl)sulfonyl)-1,2-dihydroquinoline

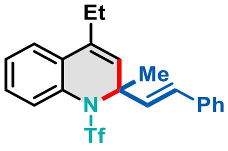

7b, 81%

59.407 mg, 81%, colorless liquid;

^{1}H NMR (400 MHz, $CDCl_3$) δ 7.55 (dd, *J* = 7.6, 1.5 Hz, 1H), 7.35 – 7.31 (m, 1H), 7.29 – 7.18 (m, 7H), 6.39 (d, *J* = 15.9 Hz, 1H), 6.12 (d, *J* = 15.9 Hz, 1H), 5.97 – 5.85 (m, 1H), 2.62 (q, *J* = 7.4 Hz, 2H), 1.86 (s, 3H), 1.23 (q, *J* = 7.2 Hz, 3H).

^{13}C{^{1}H} NMR (100 MHz, $CDCl_3$) δ 138.47, 136.16, 134.57, 131.45, 131.33, 129.60, 129.20, 128.52, 128.32, 127.90, 127.74, 127.70, 126.57, 122.85, 121.96, 120.45 (q, *J* = 326.1 Hz) 26.63, 24.45, 12.56.

HRMS (ESI-TOF) *m/z* $[M + Na]^+$ calcd for $C_{21}H_{20}F_3NNaO_2S$ 430.1059, found 430.1063.

(E)-4-isopropyl-2-methyl-2-styryl-1-((trifluoromethyl)sulfonyl)-1,2-dihydroquinoline

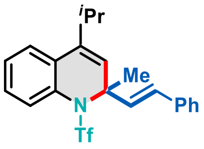

7c, 78%

57.5 mg, 78%, sticky brown solid.

^{1}**H NMR** (400 MHz, $CDCl_3$) δ 7.54 (d, *J* = 7.7 Hz, 1H), 7.36 (d, *J* = 9.2 Hz, 1H), 7.28 – 7.25 (m, 2H), 7.25 – 7.19 (m, 3H), 7.19 – 7.15 (m, 2H), 6.33 (d, *J* = 15.9 Hz, 1H), 6.08 (d, *J* = 15.9 Hz, 1H), 5.85 (s, 1H), 3.12 – 2.99 (sept, *J* = 6.7 Hz, 1H), 1.86 (s, 3H), 1.31 (d, *J* = 6.7 Hz, 3H), 1.16 (d, *J* = 6.9 Hz, 3H).

^{13}**C{^{1}H} NMR** (100 MHz, $CDCl_3$) δ 142.92, 136.12, 134.70, 131.45, 131.41, 129.55, 128.51, 128.47, 127.89, 127.84, 127.66, 127.61, 126.55, 122.83, 120.45 (*q*, *J* = 325.3 Hz), 64.26, 27.96, 26.70, 22.09, 21.40.

HRMS (ESI-TOF) *m/z* $[M + H]^+$ calcd for $C_{22}H_{23}F_3NO_2S$ 422.1396, found 422.1392.

(E)-4-butyl-2-methyl-2-styryl-1-((trifluoromethyl)sulfonyl)-1,2-dihydroquinoline

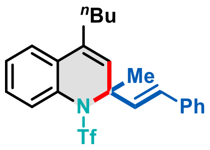

7d, 82%

64.5 mg, 82%, Brown sticky solid.

^{1}**H NMR** (400 MHz, $CDCl_3$) δ 7.54 (d, *J* = 7.8 Hz, 1H), 7.32 (d, *J* = 7.3 Hz, 1H), 7.29 – 7.17 (m, 7H), 6.38 (d, *J* = 15.9 Hz, 1H), 6.12 (d, *J* = 15.9 Hz, 1H), 5.90 (s, 1H), 2.67 – 2.46 (m, 2H), 1.85 (s, 3H), 1.62 – 1.44 (m, 4H), 1.00 (t, *J* = 7.1 Hz, 3H).

^{13}**C{^{1}H} NMR** (100 MHz, $CDCl_3$) δ 137.29, 136.15, 134.57, 131.51, 131.31, 129.87, 129.59, 128.53, 128.26, 127.90, 127.72, 127.65, 126.57, 122.97, 120.45 (q, *J* = 325.8 Hz), 64.27, 31.32, 30.44, 26.60, 22.80, 13.95.

HRMS (ESI-TOF) *m/z* $[M + H]^+$ calcd for $C_{23}H_{25}F_3NO_2S$ 436.1553, found 436.1548.

(E)-2-methyl-4-phenyl-2-styryl-1-((trifluoromethyl)sulfonyl)-1,2-dihydroquinoline

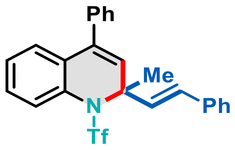

7e, 85%

69.9 mg, 85%, colorless liquid;

^{1}**H NMR** (500 MHz, $CDCl_3$) δ 7.64 (d, *J* = 8.0 Hz, 1H), 7.53 – 7.44 (m, 5H), 7.33 – 7.28 (m, 3H), 7.27 – 7.20 (m, 4H), 7.13 (dd, *J* = 7.7, 1.3 Hz, 1H), 6.55 (d, *J* = 16.0 Hz, 1H), 6.23 (d, *J* = 16.0 Hz, 1H), 6.20 (s, 1H), 1.97 (s, 3H).

$^{13}C\{^1H\}$ **NMR** (125 MHz, $CDCl_3$) δ 139.61, 137.63, 136.03, 135.04, 131.55, 131.25, 130.83, 129.94, 129.41, 128.71, 128.58, 128.57, 128.51, 128.44, 128.38, 128.27, 128.06, 127.64, 126.93, 126.64, 125.98, 120.45(q, J = 326 Hz), 64.33, 26.64.

HRMS (ESI-TOF) m/z $[M + Na]^+$ calcd for $C_{25}H_{20}F_3NNaO_2S$ 478.1059, found 478.1051.

(E)-2,6-dimethyl-4-phenyl-2-styryl-1-((trifluoromethyl)sulfonyl)-1,2-dihydroquinoline

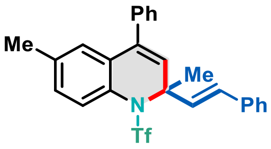

7f, 90 %

76.3 mg, 90%, yellowish liquid.

^{1}**H NMR** (400 MHz, $CDCl_3$) δ 7.55 – 7.38 (m, 6H), 7.33 – 7.20 (m, 5H), 7.08 (dd, J = 8.2, 1.3 Hz, 1H), 6.89 (d, J = 1.1 Hz, 1H), 6.53 (d, J = 16.0 Hz, 1H), 6.22 (d, J = 16.0 Hz, 1H), 6.15 (s, 1H), 2.24 (s, 3H), 1.93 (s, 3H).

$^{13}C\{^1H\}$ **NMR** (100 MHz, $CDCl_3$) δ 139.66, 137.81, 137.59, 136.09, 132.47, 131.24, 131.20, 131.11, 129.72, 129.12, 128.70, 128.59, 128.44, 128.18, 128.03, 126.67, 126.46, 120.45 (q, J = 326.1 Hz), 64.24, 26.71, 21.17.

HRMS (ESI-TOF) m/z $[M + Na]^+$ calcd for $C_{26}H_{22}F_3NNaO_2S$ 492.1216, found 492.1214.

(E)-6-methoxy-2-methyl-4-phenyl-2-styryl-1-((trifluoromethyl)sulfonyl)-1,2-dihydroquinoline

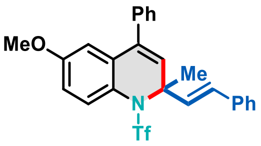

7g, 85%

74.5 mg, 85%, white solid.

^{1}**H NMR** (400 MHz, $CDCl_3$) δ 7.55 (d, J = 8.9 Hz, 1H), 7.49 – 7.44 (m, 5H), 7.33 – 7.24 (m, 5H), 6.81 (dd, J = 8.9, 2.9 Hz, 1H), 6.63 (d, J = 2.9 Hz, 1H), 6.54 (d, J = 16.0 Hz, 1H), 6.23 (d, J = 16.0 Hz, 1H), 6.20 (s, 1H), 3.70 (s, 3H), 1.95 (s, 3H).

$^{13}C\{^1H\}$ **NMR** (100 MHz, $CDCl_3$) δ 158.56, 139.62, 137.56, 136.07, 132.61, 131.77, 131.12, 129.77, 129.56, 128.74, 128.60, 128.56, 128.30, 128.07, 127.85, 126.68, 120.45 (q, J = 326.3 Hz), 113.00, 111.80, 64.27, 55.38.

HRMS (ESI-TOF) *m/z* [M + Na]+ calcd for $C_{26}H_{23}F_3NO_3S$ 486.1345, found 486.1340.

(E)-6-fluoro-2-methyl-4-phenyl-2-styryl-1-((trifluoromethyl)sulfonyl)-1,2-dihydroquinoline

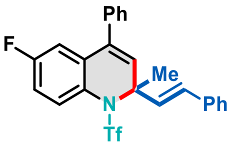

7h, 83%

71 mg, 83%, solid, m.p. 123-126 °C.

^{1}H NMR (400 MHz, $CDCl_3$) δ 7.58 (dd, *J* = 8.9, 5.1 Hz, 1H), 7.52 – 7.45 (m, 3H), 7.44 – 7.38 (m, 2H), 7.30 – 7.20 (m, 5H), 7.01 – 6.90 (m, 1H), 6.80 (dt, *J* = 12.7, 6.4 Hz, 1H), 6.50 (d, *J* = 16.0 Hz, 1H), 6.22 (s, *J* = 7.4 Hz, 1H), 6.17 (d, *J* = 16.0 Hz, 1H), 1.95 (s, 3H).

^{13}C{^{1}H} NMR (100 MHz, $CDCl_3$) δ 161.34 (d, *J* = 247.8 Hz), 139.07 (s), 136.92 (s), 135.77 (s), 133.42 (d, *J* = 8.5 Hz), 132.40 (s), 130.85 (d, *J* = 3.1 Hz), 130.48 (s), 130.25 (s, *J* = 8.7 Hz), 130.14 (d, *J* = 4.9 Hz), 128.89 (s), 128.81 (s), 128.63 (s), 128.44 (s), 128.22 (s), 126.65 (s), 120.45 (q, *J* = 326.0 Hz), 115.16 (d, *J* = 23.1 Hz), 112.75 (d, *J* = 24.5 Hz), 64.42 (s), 24.47.

HRMS (ESI-TOF) *m/z* [M + H]+ calcd for $C_{25}H_{20}F_4NO_2S$ 474.1145, found 474.1141.

(E)-6-chloro-2-methyl-4-phenyl-2-styryl-1-((trifluoromethyl)sulfonyl)-1,2-dihydroquinoline

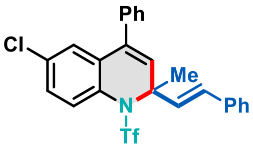

7i, 79%

69.8 mg, 79%, colorless liquid.

^{1}H NMR (500 MHz, $CDCl_3$) δ 7.56 (d, *J* = 8.6 Hz, 1H), 7.50 (m, 3H), 7.44 – 7.36 (m, 2H), 7.33 – 7.28 (m, 3H), 7.28 – 7.24 (m, 3H), 7.09 (d, *J* = 2.1 Hz, 1H), 6.52 (d, *J* = 15.9 Hz, 1H), 6.21 (s, 1H), 6.18 (d, *J* = 16.0 Hz, 1H), 1.96 (s, 3H).

^{13}C{^{1}H} NMR (125 MHz, $CDCl_3$) δ 138.85, 136.86, 135.72, 133.49, 133.47, 132.99, 132.51, 130.34, 130.23, 129.67, 128.91, 128.81, 128.63, 128.45, 128.32, 128.25, 126.67, 125.80, 120.45 (q, *J* = 325.9 Hz), 64.44, 26.58.

HRMS (ESI-TOF) *m/z* [M + Na]+ calcd for $C_{25}H_{19}ClF_3NNaO_2S$ 512.0669, found 512.0661.

(E)-6-bromo-2-methyl-4-phenyl-2-styryl-1-((trifluoromethyl)sulfonyl)-1,2-dihydroquinoline

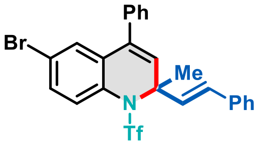

7j, 81%

78 mg, 81%, brown oil.

^{1}H NMR (400 MHz, $CDCl_3$) δ 7.53 – 7.44 (m, 4H), 7.44 – 7.36 (m, 3H), 7.33 – 7.21 (m, 6H), 6.51 (d, *J* = 16.0 Hz, 1H), 6.23 – 6.13 (m, 2H), 1.94 (s, 3H).

^{13}C{^{1}H} NMR (100 MHz, $CDCl_3$) δ 138.76, 136.85, 135.71, 134.02, 133.30, 132.56, 131.31, 130.32, 130.25, 129.96, 128.95, 128.84, 128.72, 128.66, 128.47, 128.28, 126.70, 121.94, 120.45(q, *J* = 325.8 Hz), 64.42, 26.61.

HRMS (ESI-TOF) *m/z* $[M + H]^+$ calcd for $C_{25}H_{20}BrF_3NO_2S$ 534.0345, found 534.0331.

(E)-6-bromo-2,4-dimethyl-2-styryl-1-((trifluoromethyl)sulfonyl)-1,2-dihydroquinoline

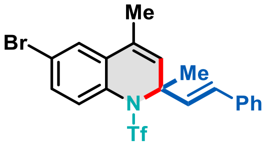

7k, 81%

69.8 mg, 82%, sticky solid.

^{1}H NMR (400 MHz, $CDCl_3$) δ 7.45 – 7.32 (m, 3H), 7.31 – 7.18 (m, 5H), 6.38 (d, *J* = 15.9 Hz, 1H), 6.10 (d, *J* = 15.9 Hz, 1H), 5.97 (s, 1H), 2.19 (s, 3H), 1.84 (s, 3H).

^{13}C{^{1}H} NMR (100 MHz, $CDCl_3$) δ 135.85, 133.88, 133.31, 132.03, 131.98, 130.76, 129.93, 129.52, 128.64, 128.59, 128.12, 126.61, 126.19, 121.56, 120.45(q, *J* = 325.8 Hz), 64.37, 26.50, 17.84.

HRMS (ESI-TOF) *m/z* $[M + K]^+$ calcd for $C_{20}H_{17}BrF_3KNO_2S$ 509.9747 found 509.9747.

(E)-methyl 2-methyl-4-phenyl-2-styryl-1-((trifluoromethyl)sulfonyl)-1,2-dihydroquinoline-6 carboxylate

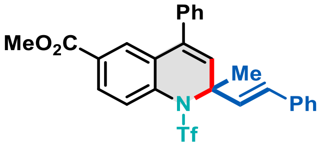

7l, 81%

81%, 75.2 mg, white solid.

^{1}H NMR (500 MHz, CDCl$_3$) δ 7.96 (d, *J* = 8.5 Hz, 1H), 7.82 (s, 1H), 7.71 (d, *J* = 8.5 Hz, 1H), 7.53 – 7.48 (m, 3H), 7.44 (m, 2H), 7.27 (m, 5H), 6.51 (d, *J* = 15.9 Hz, 1H), 6.23 (s, 1H), 6.17 (d, *J* = 15.9 Hz, 1H), 3.85 (s, 3H), 1.98 (s, 3H).

^{13}C{^{1}H} NMR (100 MHz, CDCl$_3$) δ 166.02, 139.28, 138.98, 137.01, 135.63, 132.03, 131.67, 130.34, 130.19, 129.39, 129.32, 128.93, 128.80, 128.60, 128.44, 128.24, 127.14, 126.66, 120.45 (q, j = 326.50 Hz), 64.68, 52.30, 26.59.

HRMS (ESI-TOF) *m/z* [M + H]$^+$ calcd for $C_{27}H_{23}F_3NO_4S$ 514.1294, found 514.1286.

(E)-8-fluoro-2-methyl-4-phenyl-2-styryl-1-((trifluoromethyl)sulfonyl)-1,2-dihydroquinoline

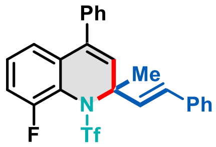

7m, 89%

89%, 76.1 mg, colorless oil.

^{1}H NMR (500 MHz, CDCl$_3$) δ 7.55 – 7.43 (m, 6H), 7.31 – 7.20 (m, 5H), 7.08 (t, *J* = 8.8 Hz, 1H), 6.93 (d, *J* = 7.6 Hz, 1H), 6.54 (d, *J* = 15.9 Hz, 1H), 6.21 (s, 1H), 6.09 (d, *J* = 15.9 Hz, 1H), 2.07 (s, 3H).

^{13}C{^{1}H} NMR (125 MHz, CDCl$_3$) δ 158.95-157.14 (d, *J* = 259.92 Hz), 139.70, 137.06, 135.71, 134.04, 132.73, 130.17, 130.11, 128.97, 128.90, 128.78, 128.73, 128.59, 128.47, 128.16, 126.64, 120.45 (q, *J* = 125.8), 116.72-116.47(q, *J*= 21.14Hz), 64.55, 25.97.

HRMS (ESI-TOF) *m/z* [M + H]$^+$ calcd for $C_{25}H_{20}F_4NO_2S$ 474.1145, found 474.1142.

(E)-2,6,8-trimethyl-4-phenyl-2-styryl-1-((trifluoromethyl)sulfonyl)-1,2-dihydroquinoline

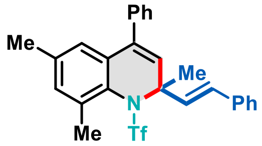

7n, 85%

74.2 mg, 85%, colorless oil.

^{1}H NMR (500 MHz, CDCl$_3$) δ 7.49 (s, *J* = 26.3 Hz, 5H), 7.31 – 7.23 (m, 3H), 7.18 (d, *J* = 7.2 Hz, 2H), 6.97 (s, *J* = 27.2 Hz, 1H), 6.72 (s, *J* = 28.5 Hz, 1H), 6.43 (d, *J* = 15.9 Hz, 1H), 6.12 (s, 1H), 6.09 (d, *J* = 16.0 Hz, 1H), 2.50 (s, 3H), 2.20 (s, 3H), 2.04 (s, 3H).

$^{13}C\{^1H\}$ NMR (125 MHz, $CDCl_3$) δ 140.74, 137.76, 137.64, 137.53, 135.99, 132.35, 132.05, 131.60, 131.10, 129.73, 128.62, 128.56, 128.39, 127.99, 126.59, 124.37, 120.45 (q, J = 327.3 Hz) , 64.13, 26.11, 21.12, 19.42.

HRMS (ESI-TOF) m/z $[M + H]^+$ calcd for $C_{27}H_{25}F_3NO_2S$ 484.1553 found 484.1542.

(E)-6,8-dimethyl-6-styryl-5-((trifluoromethyl)sulfonyl)-5,6-dihydro-[1,3]dioxolo[4,5-g]quinoline

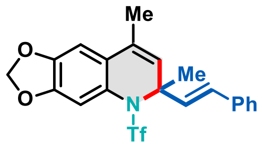

7o, 78%

61.6 mg, 78%, sticky liquid.

^1H NMR (500 MHz, $CDCl_3$) δ 7.32 – 7.23 (m, 1H), 7.04 (s, J = 16.6 Hz, 1H), 6.77 (s, 1H), 6.41 (d, J = 15.9 Hz, 1H), 6.14 (d, J = 15.9 Hz, 1H), 5.99 (dd, J = 10.3, 1.2 Hz, 1H), 5.83 (s, 1H), 2.16 (s, J = 1.2 Hz, 3H), 1.84 (s, 3H).

$^{13}C\{^1H\}$ NMR (125 MHz, $CDCl_3$) δ 147.09, 146.59, 136.13, 132.76, 131.60, 129.36, 128.79, 128.55, 128.39, 127.93, 127.07, 126.62, 120.45 (q, J = 125.3 Hz), 109.59, 102.84, 101.80, 64.38, 26.54, 18.25.

HRMS (ESI-TOF) m/z $[M + K]^+$ calcd for $C_{21}H_{18}F_3KNO_4S$ 476.0540 found 476.0537.

(E)-2,4-dimethyl-2-(4-methylstyryl)-1-((trifluoromethyl)sulfonyl)-1,2-dihydroquinoline

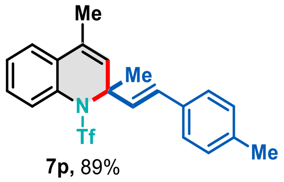

7p, 89%

64.5 mg, 89%, colorless liquid.

^1H NMR (400 MHz, $CDCl_3$) δ 7.52 (d, J = 8.1 Hz, 1H), 7.35 – 7.24 (m, 3H), 7.20 – 7.16 (m, 1H), 7.13 – 7.03 (m, 3H), 6.57 (d, J = 15.8 Hz, 1H), 5.91 (s, 1H), 5.88 (d, J = 15.8 Hz, 1H), 2.22 (s, 3H), 2.02 (s, 3H), 1.89 (s, 3H).

$^{13}C\{^1H\}$ NMR (100 MHz, $CDCl_3$) δ 135.59, 134.47, 132.99, 132.38, 132.28, 130.70, 130.03, 128.17, 127.88, 127.76, 127.65, 126.06, 123.04, 121.97(q, J = 325.1), 64.49, 26.51, 19.47, 17.89.

HRMS (ESI-TOF) m/z $[M + H]^+$ calcd for $C_{21}H_{20}F_3NNO_2S$ 408.1240 found 408.1240.

(E)-2-(4-chlorostyryl)-2,4-dimethyl-1-((trifluoromethyl)sulfonyl)-1,2-dihydroquinoline

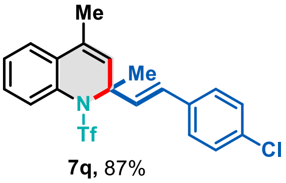

7q, 87%

66.8 mg, 87%, yellowish liquid;

^{1}H NMR (500 MHz, $CDCl_3$) δ 7.55 (d, *J* = 7.6 Hz, 1H), 7.39 (d, *J* = 7.8 Hz, 2H), 7.34 (d, *J* = 7.4 Hz, 1H), 7.33 – 7.25 (m, 2H), 7.10 (d, *J* = 7.9 Hz, 2H), 6.37 (d, *J* = 15.9 Hz, 1H), 6.15 (d, *J* = 15.9 Hz, 1H), 5.95 (s, 1H), 2.23 (s, 3H), 1.85 (s, 3H).

^{13}C{^{1}H} NMR (125 MHz, $CDCl_3$) δ 135.06, 134.18, 133.03, 132.08, 131.99, 131.65, 130.40, 128.48, 128.11, 128.03, 127.96, 127.79, 123.13, 121.78, 125.45(q, *J* = 326), 64.14, 26.43, 17.95.

HRMS (ESI-TOF) *m/z* $[M + Na]^+$ calcd for $C_{20}H_{17}ClF_3NNaO_2S$ 450.0513, found 450.0519.

(E)-2-(4-bromostyryl)-2,4-dimethyl-1-((trifluoromethyl)sulfonyl)-1,2-dihydroquinoline

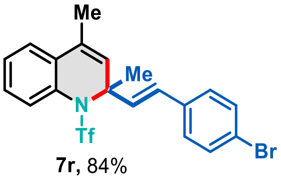

7r, 84%

71.21 mg, 84%, colorless liquid.

^{1}H NMR (500 MHz, $CDCl_3$) δ 7.56 (dd, *J* = 7.8, 1.3 Hz, 1H), 7.37 – 7.33 (m, 1H), 7.32 – 7.26 (m, 2H), 7.24 (d, *J* = 8.5 Hz, 2H), 7.17 (d, *J* = 8.5 Hz, 2H), 6.39 (d, *J* = 16.0 Hz, 1H), 6.14 (d, *J* = 15.9 Hz, 1H), 5.96 (s, 1H), 2.24 (d, *J* = 1.2 Hz, 3H), 1.86 (s, 3H).

^{13}C{^{1}H} NMR (125 MHz, $CDCl_3$) δ 134.63, 134.19, 133.62, 133.01, 132.10, 131.87, 130.44, 128.70, 128.43, 128.03, 127.95, 127.81, 123.13, 120.25 (q, *J* = 325.8), 64.16, 26.46, 17.94.

HRMS (ESI-TOF) *m/z* $[M + H]^+$ calcd for $C_{20}H_{18}BrF_3NO_2S$ 472.0188, found 472.0189.

(E)-2-(4-fluorostyryl)-2,4-dimethyl-1-((trifluoromethyl)sulfonyl)-1,2-dihydroquinoline

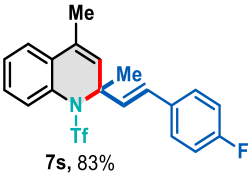

7s, 83%

61.27 mg, 83%, colorless liquid.

^{1}**H NMR** (500 MHz, $CDCl_3$) δ 7.55 (dd, *J* = 7.7, 1.3 Hz, 1H), 7.36 – 7.33 (m, 1H), 7.33 – 7.25 (m, 2H), 7.24 – 7.17 (m, 2H), 6.96 (t, *J* = 8.7 Hz, 2H), 6.39 (d, *J* = 16.0 Hz, 1H), 6.08 (d, *J* = 15.9 Hz, 1H), 5.95 (s, 1H), 2.23 (d, *J* = 1.3 Hz, 3H), 1.85 (s, 3H).

^{13}**C{^{1}H} NMR** (125 MHz, $CDCl_3$) δ 162.50 (d, *J* = 247.5 Hz), 134.21 (s), 132.91 (s), 132.28 (d, *J* = 3.3 Hz), 132.12 (s), 130.96 (s), 130.55 (s), 128.47 (s), 128.17 (s), 128.11 (s), 128.04 (s), 127.92 (s), 127.75 (s), 123.10 (s), 120.25 (q, *J* = 325.8 Hz), 64.18 (s), 26.52 (s), 17.94 (s).

HRMS (ESI-TOF) *m/z* $[M + H]^+$ calcd for $C_{20}H_{18}F_4NO_2S$ 412.0989 found 412.0995.

(E)-4-(2-(2,4-dimethyl-1-((trifluoromethyl)sulfonyl)-1,2-dihydroquinolin-2-yl)vinyl)benzonitrile

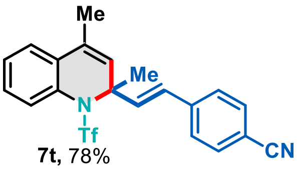

7t, 78%

58.68 mg, 78%, sticky solid.

^{1}**H NMR** (500 MHz, $CDCl_3$) δ 7.55 (d, *J* = 8.0 Hz, 3H), 7.37 – 7.26 (m, 5H), 6.44 (d, *J* = 16.0 Hz, 1H), 6.28 (d, *J* = 16.0 Hz, 1H), 5.95 (s, 1H), 2.23 (s, 3H), 1.85 (s, 3H).

^{13}**C{^{1}H} NMR** (125 MHz, $CDCl_3$) δ 140.61 (s), 135.18 (s), 134.08 (s), 133.33 (s), 132.36 (s), 131.93 (s), 129.95 (s), 128.10 (s), 127.99 (s), 127.96 (s), 127.90 (s), 127.08 (s), 123.22 (s), 120.25 (q, *J* = 325.8 Hz), 118.75 (s), 111.21 (s), 63.90 (s), 26.34 (s), 17.97 (s).

HRMS (ESI-TOF) *m/z* $[M + H]^+$ calcd for $C_{21}H_{18}F_3N_2O_2S$ 419.1036 found 419.1030.

(E)-2,4-dimethyl-2-(4-(trifluoromethyl)styryl)-1-((trifluoromethyl)sulfonyl)-1,2-dihydroquinoline

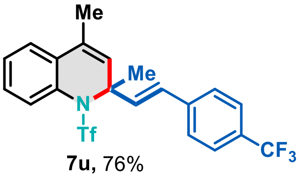

7u, 76%

63.0 mg, 76%, brown oil.

^{1}**H NMR** (400 MHz, $CDCl_3$) δ 7.55 – 7.45 (m, 3H), 7.35 – 7.21 (m, 5H), 6.42 (d, *J* = 16.0 Hz, 1H), 6.22 (d, *J* = 16.0 Hz, 1H), 5.93 (s, 1H), 2.21 (d, *J* = 1.3 Hz, 3H), 1.83 (s, 3H).

^{13}**C{^{1}H} NMR** (100 MHz, $CDCl_3$) δ 140.08, 134.32 (s), 134.08 (s), 133.37 (s), 132.19 (s), 130.36 (s), 129.90, 128.44 (s), 128.19 (d, *J* = 1.5 Hz), 128.00 (s), 126.91 (s), 125.66 (q, *J* = 3.9 Hz), 123.32 (s), 120.45 (q, *J* = 125.2 Hz), 64.18 (s), 26.57 (s), 18.12 (s).

HRMS (ESI-TOF) *m/z* [M + K]$^+$ calcd for $C_{21}H_{17}F_6KNO_2S$ 500.0516, found 500.0508.

(E)-2-(3-fluorostyryl)-2,4-dimethyl-1-((trifluoromethyl)sulfonyl)-1,2-dihydroquinoline

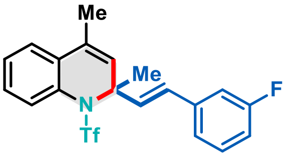

7v, 82%

60.53 mg, 82%, colorless liquid.

^{1}H NMR (400 MHz, $CDCl_3$) δ 7.56 – 7.47 (m, 1H), 7.34 – 7.29 (m, 1H), 7.29 – 7.22 (m, 2H), 7.22 – 7.16 (m, 1H), 6.97 (d, *J* = 7.7 Hz, 1H), 6.94 – 6.85 (m, *J* = 8.8 Hz, 2H), 6.37 (d, *J* = 15.9 Hz, 1H), 6.14 (d, *J* = 15.9 Hz, 1H), 5.92 (d, *J* = 0.9 Hz, 1H), 2.21 (d, *J* = 1.4 Hz, 3H), 1.83 (s, 3H).

^{13}C{^{1}H} NMR (100 MHz, $CDCl_3$) δ 162.95 (d, *J* = 245.6 Hz), 138.45 (d, *J* = 7.8 Hz), 134.18 (s), 133.06 (s), 132.65 (s), 132.06 (s), 130.37 (s), 129.99 (d, *J* = 8.4 Hz), 128.53 (d, *J* = 2.4 Hz), 128.02 (s), 127.97 (s), 127.79 (s), 123.13 (s), 122.44 (d, *J* = 2.7 Hz), 120.45 (q, *J* = 325.8 Hz), 114.73 (d, *J* = 21.4 Hz), 113.08 (d, *J* = 21.8 Hz), 64.07 (s), 26.45 (s), 17.94 (s).

HRMS (ESI-TOF) *m/z* [M + H]$^+$ calcd for $C_{20}H_{18}F_4NO_2S$ 412.0989, found 412.0986.

(E)-2-(3-chlorostyryl)-2,4-dimethyl-1-((trifluoromethyl)sulfonyl)-1,2-dihydroquinoline

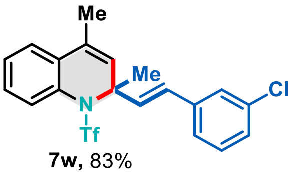

7w, 83%

63.8 mg, 83%, colorless liquid.

^{1}H NMR (400 MHz, $CDCl_3$) δ 7.51 (m, 1H), 7.33 – 7.22 (m, 1H), 7.20 – 7.14 (m, 3.65H), 7.11 – 7.01 (m, 3H), 6.33 (d, *J* = 15.9 Hz, 1H), 6.12 (d, *J* = 15.9 Hz, 1H), 5.90 (d, *J* = 1.1 Hz, 1H), 2.20 (d, *J* = 1.5 Hz, 3H), 1.82 (s, 3H).

^{13}C{^{1}H} NMR (100 MHz, $CDCl_3$) δ 137.96 (s), 134.48 (s), 134.18 (s), 133.09 (s), 132.76 (s), 132.03 (s), 130.32 (s), 129.74 (s), 128.31 (s), 128.01 (s), 127.99 (s), 127.85 (s), 127.78 (s), 126.52 (s), 124.71 (s), 123.12 (s), 120.45 (q, *J* = 325.8 Hz), 64.06 (s), 26.48 (s), 17.93 (s).

HRMS (ESI-TOF) *m/z* [M + Na]$^+$ calcd for $C_{20}H_{17}ClF_3NNaO_2S$ 450.0513 found 450.0507.

(E)-2-(2-chloro-6-fluorostyryl)-2,4-dimethyl-1-((trifluoromethyl)sulfonyl)-1,2-dihydroquinoline

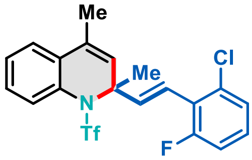

7x, 79%

63.0 mg, 79%, colorless liquid.

^{1}H NMR (500 MHz, $CDCl_3$) δ 7.58 – 7.51 (m, 1H), 7.36 – 7.24 (m, 3H), 7.13 – 7.03 (m, 2H), 6.91 (m,1H), 6.47 (d, J = 16.3 Hz, 1H), 6.27 (d, J = 16.3 Hz, 1H), 5.92 (d, J = 0.9 Hz, 1H), 2.23 (d, J = 1.4 Hz, 3H), 1.93 (s, 3H).

^{13}C{^{1}H} NMR (125 MHz, $CDCl_3$) δ 160.81 (d, J = 252.2 Hz), 138.71 (d, J = 10.5 Hz), 134.43 (d, J = 5.3 Hz), 134.37 (s), 133.56 (s), 132.16 (s), 130.09 (s), 128.46 (d, J = 10.0 Hz), 128.21 (s), 127.88 (s), 127.68 (s), 125.33 (d, J = 3.5 Hz), 123.15 (s), 123.04 (s), 120.45 (q, J = 325.6 Hz), 120.26 (s), 114.44 (d, J = 23.4 Hz), 64.65 (s), 26.31 (s), 17.90 (s).

HRMS (ESI-TOF) *m/z* $[M + H]^+$ calcd for $C_{20}H_{17}ClF_4NO_2S$ 446.0599 found 446.0597.

(E)-2-(2,4-dichlorostyryl)-2,4-dimethyl-1-((trifluoromethyl)sulfonyl)-1,2-dihydroquinoline

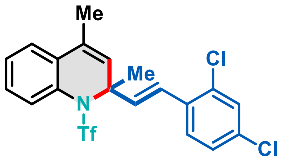

7y, 81%

67.2 mg, 81%, sticky solid.

^{1}H NMR (400 MHz, $CDCl_3$) δ 7.59 – 7.45 (m, 1H), 7.36 – 7.24 (m, 4H), 7.19 (dd, J = 7.8, 1.2 Hz, 1H), 7.08 (t, J = 7.9 Hz, 1H), 6.78 (d, J = 15.9 Hz, 1H), 6.06 (d, J = 15.9 Hz, 1H), 5.94 (s, 1H), 2.22 (d, J = 1.4 Hz, 3H), 1.84 (s, 3H).

^{13}C{^{1}H} NMR (100 MHz, $CDCl_3$) δ 137.02 (s), 135.00 (s), 134.16 (s), 133.36 (s), 133.20 (s), 132.08 (s), 131.38 (s), 130.01 (s), 129.51 (s), 128.09 (s), 128.00 (s), 127.84 (s), 127.19 (s), 126.61 (s), 125.46 (s), 123.27 (s), 120.45 (q, J = 325.7 Hz), 64.10 (s), 26.29 (s), 17.94 (s).

HRMS (ESI-TOF) *m/z* $[M + H]^+$ calcd for $C_{20}H_{17}Cl_2F_3NO_2S$ 462.0304 found 462.0294.

(E)-2-(4-bromostyryl)-2-methyl-4-phenyl-1-((trifluoromethyl)sulfonyl)-1,2-dihydroquinoline

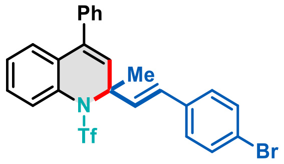

7z, 84%

83.6 mg, 87%, white solid.

^{1}H NMR (500 MHz, $CDCl_3$) δ 7.64 (dd, *J* = 8.1, 1.1 Hz, 1H), 7.53 – 7.44 (m, 5H), 7.43 – 7.39 (m, 2H), 7.31 (td, *J* = 7.8, 1.6 Hz, 1H), 7.23 (td, *J* = 7.6, 1.2 Hz, 1H), 7.16 – 7.08 (m, 3H), 6.49 (d, *J* = 16.0 Hz, 1H), 6.23 (d, *J* = 16.0 Hz, 1H), 6.19 (s, 1H), 1.96 (s, 3H).

^{13}C{^{1}H} NMR (125 MHz, $CDCl_3$) δ 139.75, 137.52, 134.99, 134.95, 131.71, 131.56, 131.50, 130.95, 128.82, 128.74, 128.58, 128.55, 128.46, 128.43, 128.16, 127.70, 126.03, 121.96, 120.45 (q, *J* = 326 Hz), 64.22, 26.51.

HRMS (ESI-TOF) *m/z* $[M + Na]^+$ calcd for $C_{25}H_{19}BrF_3NNaO_2S$ 556.0164 found 556.0151.

(E)-2-(4-chlorostyryl)-2,6-dimethyl-4-phenyl-1-((trifluoromethyl)sulfonyl)-1,2-dihydroquinoline

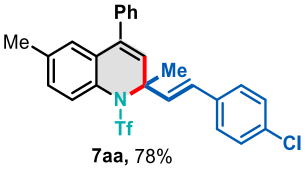

7aa, 78%

70 mg, 78%, viscous liquid.

^{1}H NMR (500 MHz, $CDCl_3$) δ 7.53 – 7.46 (m, 4H), 7.45 – 7.40 (m, 2H), 7.25 (d, *J* = 8.5 Hz, 2H), 7.18 (d, *J* = 8.5 Hz, 2H), 7.09 (d, *J* = 9.7 Hz, 1H), 6.89 (s, 1H), 6.48 (d, *J* = 16.0 Hz, 1H), 6.19 (d, *J* = 16.0 Hz, 1H), 6.13 (s, 1H), 2.25 (s, 3H), 1.91 (s, 3H), 1.61 (s, 3H).

13C NMR (125 MHz, CDC13) δ 139.74, 137.68, 137.63, 134.54, 133.74, 132.39, 131.67, 131.11, 130.94, 129.15, 128.73, 128.68, 128.54, 128.46, 128.14, 127.84, 126.46, 121.69, 119.09(q, *J* = 326.0 Hz), 64.08, 26.57, 21.15.

HRMS (ESI-TOF) *m/z* $[M + Na]^+$ calcd for $C_{27}H_{25}ClF_3NNaO_2S$ 542.1139, found 542.1145.

(E)-2,4-dimethyl-2-(2-(naphthalen-2-yl)vinyl)-1-((trifluoromethyl)sulfonyl)-1,2-dihydroquinoline

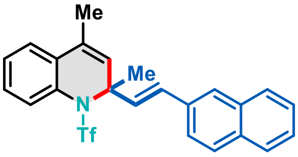

7ab, 84%

67.0 mg, 84%, yellowish liquid.

^{1}H NMR (400 MHz, $CDCl_3$) δ 7.80 (d, *J* = 8.1 Hz, 1H), 7.74 (d, *J* = 8.0 Hz, 1H), 7.62 – 7.57 (m, 1H), 7.48 – 7.44 (m, 1H), 7.43 – 7.39 (m, 3H), 7.39 – 7.33 (m, 2H), 7.33 – 7.25 (m, 2H), 7.07 (d, *J* = 15.7 Hz, 1H), 6.04 (d, *J* = 15.7 Hz, 1H), 6.01 (s, *J* = 1.0 Hz, 1H), 2.28 (d, *J* = 1.4 Hz, 3H), 1.97 (s, 3H).

$^{13}C\{^1H\}$ **NMR** (100 MHz, $CDCl_3$) δ 134.58 (s), 134.34 (s), 134.25 (s), 133.37 (s), 133.25 (s), 132.43 (s), 131.14 (s), 130.73 (s), 128.44 (s), 128.29 (s), 128.14 (s), 127.98 (d, J = 6.4 Hz), 127.94 (s), 127.74 (s), 125.96 (s), 125.84 (s), 125.54 (s), 124.26 (s), 123.76 (s), 123.17 (s), 120.45 (q, J = 325.8 Hz), 64.54 (s), 26.43 (s), 17.97 (s).

HRMS (ESI-TOF) *m/z* $[M + H]^+$ calcd for $C_{24}H_{21}F_3NO_2S$ 444.1240 found 444.1235.

(E)-2-(2-([1,1'-biphenyl]-4-yl)vinyl)-2,4-dimethyl-1-((trifluoromethyl)sulfonyl)-1,2-dihydroquinoline

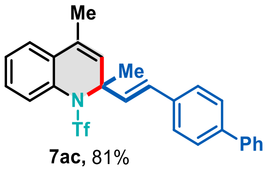

7ac, 81%

CCDC: 2074148

68.3 mg, 81%, colorless liquid.

^{1}**H NMR** (500 MHz, $CDCl_3$) δ 7.58 (d, J = 7.5 Hz, 3H), 7.52 (d, J = 8.1 Hz, 2H), 7.45 (t, J = 7.6 Hz, 2H), 7.39 – 7.34 (m, 2H), 7.34 – 7.26 (m, 4H), 6.47 (d, J = 15.9 Hz, 1H), 6.21 (d, J = 15.9 Hz, 1H), 5.98 (s, 1H), 2.25 (s, 3H), 1.89 (s, 3H).

$^{13}C\{^1H\}$ **NMR** (125 MHz, $CDCl_3$) δ 140.80 (s), 140.56 (s), 135.16 (s), 134.30 (s), 132.90 (s), 132.17 (s), 131.31 (s), 130.68 (s), 129.17 (s), 128.81 (s), 128.07 (s), 127.92 (s), 127.73 (s), 127.42 (s), 127.24 (s), 127.01 (s), 126.94 (s), 123.09 (s), 120.45 (q, J = 326.0 Hz), 64.32 (s), 26.58 (s), 17.95 (s).

HRMS (ESI-TOF) *m/z* $[M + H]^+$ calcd for $C_{26}H_{22}F_3NO_2S$ 470.1396 found 470.1389.

(1R,2R,5R)-2-isopropyl-5-methylcyclohexyl 4-((E)-2-(2,4-dimethyl-1-((trifluoromethyl)sulfonyl)-1,2-dihydroquinolin-2-yl)vinyl)benzoate

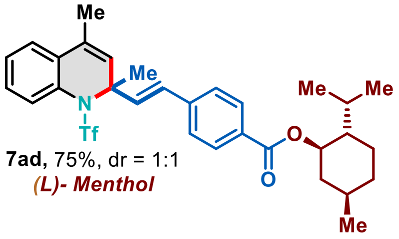

7ad, 75%, dr = 1:1
(L)- Menthol

86 mg, 75%, sticky white solid.

^{1}**H NMR** (400 MHz, $CDCl_3$) δ 7.92 (d, J = 8.3 Hz, 2H), 7.58 – 7.46 (m, 1H), 7.34 – 7.28 (m, 1H), 7.28 – 7.21 (m, 4H), 6.43 (d, J = 16.0 Hz, 1H), 6.32 – 6.15 (m, 1H), 5.93 (s, 1H), 4.90 (td, J = 10.8, 4.3 Hz, 1H), 2.21 (d, J = 1.4 Hz, 3H), 2.14 – 2.05 (m, 1H), 1.95 – 1.88 (m, 1H), 1.84 (s, 3H), 1.77 – 1.67 (m, 2H), 1.59 – 1.47 (m, 2H), 1.18 – 1.05 (m, 2H), 0.98 – 0.85 (m, 7H), 0.77 (d, J = 6.9 Hz, 3H).

$^{13}C\{^1H\}$ **NMR** (100 MHz, $CDCl_3$) δ 165.70, 140.39, 134.18, 133.73, 133.67, 133.15, 132.06, 130.30, 130.07, 129.84, 128.78, 128.74, 128.02, 127.98, 127.80, 126.40, 123.13, 120.45 (q, J = 325.8 Hz),

74.85, 64.11, 47.27, 40.96, 34.31, 31.44, 26.56, 26.53, 26.39, 23.68, 23.65, 22.05, 20.74, 17.94, 16.57, 16.54.

HRMS (ESI-TOF) *m/z* [M + K]$^+$ calcd for $C_{31}H_{36}F_3KNO_4S$ 614.1949 found 614.1944.

(E)-2-isopropyl-5-methylphenyl4-(2-(2,4-dimethyl-1-((trifluoromethyl)sulfonyl)-1,2 dihydroquinolin-2-yl)vinyl)benzoate

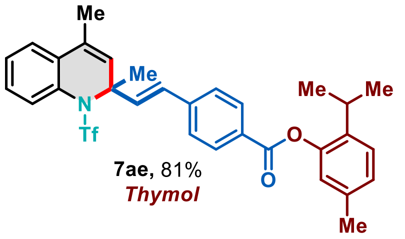

81%, 92 mg, colorless liquid.

^{1}H NMR (500 MHz, $CDCl_3$) δ 8.11 (d, *J* = 8.3 Hz, 1H), 7.57 (d, *J* = 7.5 Hz, 1H), 7.39 – 7.33 (m, 3H), 7.33 – 7.27 (m, 2H), 7.27 – 7.24 (m, 1H), 7.08 (d, *J* = 7.8 Hz, 1H), 6.50 (d, *J* = 16.0 Hz, 1H), 6.31 (d, *J* = 15.9 Hz, 1H), 5.97 (s, 1H), 3.07 – 2.97 (m, 1H), 2.36 (s, *J* = 7.2 Hz, 3H), 2.25 (d, *J* = 1.0 Hz, 3H), 1.88 (s, 3H), 1.21 (d, *J* = 6.9 Hz, 6H).

^{13}C{^{1}H} NMR (125 MHz, $CDCl_3$) δ 164.96, 148.10, 141.24, 137.13, 136.67, 134.33, 134.19, 133.22, 132.04, 130.45, 130.23, 129.55, 128.80, 128.61, 128.04, 127.85, 127.21, 126.69, 126.50, 123.18, 122.84, 120.45 (q, *J* = 325.7 Hz)., 64.08, 27.31, 26.41, 23.02, 20.87, 17.98.

HRMS (ESI-TOF) *m/z* [M + Na]$^+$ calcd for $C_{31}H_{30}F_3NNaO_4S$ 592.1740 found 592.1748.

(3S,8S,9S,10R,13R,14S,17R)-10,13-dimethyl-17-((S)-6-methylheptan-2-yl)-2,3,4,7,8,9,10,11,12,13,14,15,16,17-tetradecahydro-1H-cyclopenta[a]phenanthren-3-yl 4-((E)-2-(2,4-dimethyl-1-((trifluoromethyl)sulfonyl)-1,2-dihydroquinolin-2-yl)vinyl)benzoate

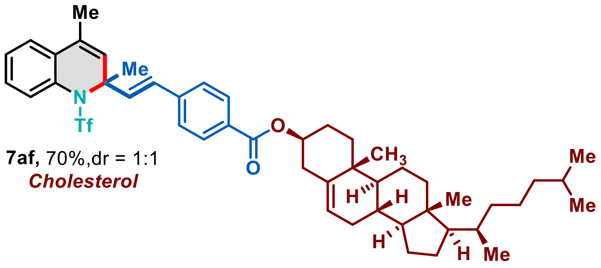

112 mg, 70%, sticky solid.

^{1}H NMR (400 MHz, $CDCl_3$) δ 7.91 (d, *J* = 8.1 Hz, 2H), 7.53 (d, *J* = 7.4 Hz, 1H), 7.35 – 7.20 (m, 5H), 6.43 (d, *J* = 16.0 Hz, 1H), 6.23 (d, *J* = 16.0 Hz, 1H), 5.93 (s, 1H), 5.41 (d, *J* = 3.7 Hz, 1H), 4.93 – 4.73 (m, 1H), 2.44 (d, *J* = 7.7 Hz, 2H), 2.21 (s, 3H), 2.08 – 1.90 (m, 4H), 1.84 (s, 3H), 1.78 – 1.66 (m, 1H), 1.65 – 1.44 (m, 7H), 1.31 (m, 5H), 1.25 – 1.08 (m, 7H), 1.06 (s, 3H), 1.03 – 0.96 (m, 2H), 0.93 (d, *J* = 6.4 Hz, 3H), 0.88 (d, *J* = 6.4 Hz, 7H), 0.69 (s, 3H).

$^{13}C\{^1H\}$ **NMR** (100 MHz, $CDCl_3$) δ 165.59, 140.38, 139.62, 134.18, 133.67, 133.13, 132.05, 130.31, 130.07, 129.82, 128.77, 128.03, 127.98, 127.80, 126.37, 123.14, 122.81, 120.45 (q, J = 325.7 Hz), 74.60, 64.11, 56.70, 56.15, 50.05, 42.33, 39.75, 39.54, 38.21, 37.03, 36.65, 36.21, 35.83, 31.95, 31.89, 28.26, 28.04, 27.88, 26.39, 24.32, 23.86, 22.86, 22.60, 21.07, 19.39, 18.75, 17.95, 11.89.

HRMS (ESI-TOF) *m/z* $[M + H]^+$ calcd for $C_{48}H_{63}F_3NO_2S$ 806.4424 found 806.4428.

(E)-3,7-dimethyloct-6-en-1-yl 4-(2-(2,4-dimethyl-1-((trifluoromethyl)sulfonyl)-1,2-dihydroquinolin-2-yl)vinyl)benzoate

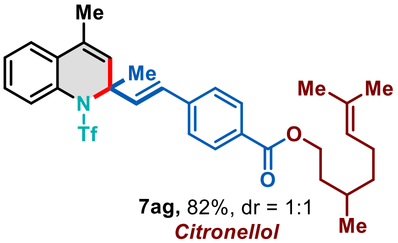

7ag, 82%, dr = 1:1
Citronellol

82%, 94 mg, colorless liquid

^1H **NMR** (500 MHz, CDCl3) δ 7.93 (d, J = 8.2 Hz, 2H), 7.55 (d, J = 7.6 Hz, 1H), 7.34 (d, J = 7.9 Hz, 1H), 7.32 – 7.24 (m, 4H), 6.46 (d, J = 16.0 Hz, 1H), 6.26 (d, J = 16.0 Hz, 1H), 5.95 (s, 1H), 5.12 (t, J = 6.8 Hz, 1H), 4.42 – 4.25 (m, 2H), 2.23 (s, 3H), 2.02 (m, 2H), 1.86 (s, 3H), 1.80 (m, 1H), 1.70 (s, 3H), 1.66 (m, 1H), 1.62 (s, 3H), 1.58 (m, 1H), 1.47 – 1.38 (m, 1H), 1.29 – 1.22 (m, 1H), 0.98 (d, J = 6.5 Hz, 3H).

$^{13}C\{^1H\}$ **NMR** (125 MHz, $CDCl_3$) δ 166.26, 140.49, 134.20, 133.78, 131.37, 130.33, 129.80, 129.76, 128.75, 128.01, 127.98, 127.78, 126.42, 124.56, 123.13, 120.25 (q, J = 325.7 Hz). 64.10, 63.52, 36.98, 35.50, 29.60, 26.37, 25.69, 25.40, 19.49, 17.91, 17.65.

HRMS (ESI-TOF) *m/z* $[M + Na]^+$ calcd for $C_{31}H_{36}F_3NNaO_4S$ 598.2209 found 598.2198.

(E)-2,4-dimethyl-2-(4-methylstyryl)-2H-chromene

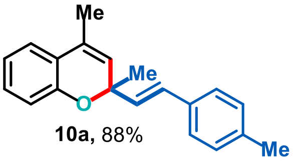

10a, 88%

44.49mg, 88%, colorless sticky liquid

^1H **NMR (500 MHz, $CDCl_3$)** δ 7.27 (d, J = 8.2 Hz, 2H), 7.21 – 7.15 (m, 2H), 7.12 (d, J = 7.8 Hz, 2H), 6.91 (m, 2H), 6.57 (d, J = 16.0 Hz, 1H), 6.27 (d, J = 16.2 Hz, 1H), 5.51 (d, J = 1.5 Hz, 1H), 2.34 (s, 3H), 2.11 (d, J = 1.5 Hz, 3H).

$^{13}C\{^1H\}$ **NMR (126 MHz, $CDCl_3$)** δ 153.00, 137.55, 134.00, 131.68, 129.30, 129.21, 128.89, 128.86, 126.64, 124.93, 123.56, 120.79, 116.39, 77.50, 27.68, 21.31, 18.21.

HRMS (ESI-TOF) *m/z* $[M + H]^+$ calcd for $C_{20}H_{21}O_2$ 277.1587, found 277.1582.

***(E)*-2-(4-chlorostyryl)-2,4-dimethyl-2*H*-chromene**

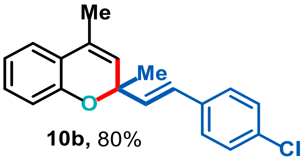

10b, 80%

47.36mg, 80%, colorless sticky liquid

^{1}H NMR (500 MHz, $CDCl_3$) δ 7.29 – 7.26 (m, 4H), 7.17 (m, 2H), 6.91 (t, *J* = 7.6 Hz, 1H), 6.89 (d, *J* = 8.1 Hz, 1H), 6.54 (d, *J* = 16.0 Hz, 1H), 6.27 (d, *J* = 16.0 Hz, 1H), 5.49 (d, *J* = 1.5 Hz, 1H), 2.10 (d, *J* = 1.5 Hz, 3H), 1.63 (s, 3H).

^{13}C{^{1}H} NMR (125 MHz, $CDCl_3$) δ 152.89, 135.35, 133.38, 133.33, 129.33, 129.14, 128.75, 127.96, 127.68, 124.53, 123.64, 122.94, 120.93, 116.37, 77.36, 27.69, 18.22.

HRMS (ESI-TOF) *m/z* $[M+H]^+$ calcd for $C_{19}H_{18}ClO_2$ 297.1041, found 297.1031.

***(E)*-2,4-dimethyl-2-(2-(naphthalen-1-yl)vinyl)-2*H*-chromene**

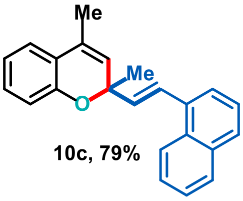

10c, 79%

49.29mg, 79%, colorless liquid

^{1}H NMR (500 MHz, $CDCl_3$) δ 7.96 – 7.89 (m, 1H), 7.85 (dd, *J* = 6.3, 3.2 Hz, 1H), 7.78 (d, *J* = 8.2 Hz, 1H), 7.57 (d, *J* = 8.1 Hz, 1H), 7.50 (m, 2H), 7.46 – 7.41 (m, 1H), 7.38 (d, *J* = 15.7 Hz, 1H), 7.24 (m, 2H), 7.02 – 6.94 (m, 2H), 6.30 (d, *J* = 15.7 Hz, 1H), 5.61 (s, 1H), 2.16 (d, *J* = 1.3 Hz, 3H), 1.75 (s, 3H).

^{13}C{^{1}H} NMR (125 MHz, $CDCl_3$) δ 153.03, 135.43, 134.68, 133.57, 131.29, 129.24, 129.22, 128.45, 127.89, 126.21, 125.97, 125.73, 125.55, 124.79, 123.84, 123.55, 123.21, 120.86, 116.42, 77.51, 27.66, 18.12.

HRMS (ESI-TOF) *m/z* $[M + H]^+$ calcd for $C_{23}H_{21}O$ 331.1587, found 331.1575.

***(E)*-2-(2-cyclohexylvinyl)-2,4-dimethyl-2*H*-chromene**

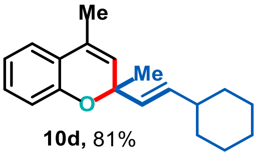

10d, 81%

42.90, 81%, colorless liquid

^{1}H NMR (400 MHz, $CDCl_3$) δ 7.15 – 7.08 (m, 2H), 6.86 (td, *J* = 7.5, 1.2 Hz, 1H), 6.80 (dd, *J* = 7.9, 0.9 Hz, 1H), 5.60 (dd, *J* = 15.7, 6.3 Hz, 1H), 5.51 (dd, *J* = 15.8, 0.9 Hz, 1H), 5.38 (d, *J* = 1.4 Hz, 1H), 2.03

(d, J = 1.4 Hz, 3H), 1.95 – 1.85 (m, 1H), 1.65 (dd, J = 14.5, 6.9 Hz, 4H), 1.48 (s, 6H), 1.29 – 0.94 (m, 6H).

^{13}C NMR (100 MHz, $CDCl_3$) δ 152.91, 136.12, 130.41, 128.85, 128.14, 125.65, 123.24, 123.10, 120.43, 116.25, 77.28, 40.19, 32.74, 27.22, 26.15, 25.99, 18.03.

HRMS (ESI-TOF) *m/z* $[M + H]^+$ calcd for $C_{19}H_{25}O$ 269.1900, found 269.1960.

(E)-2-methyl-4-phenyl-2-styryl-2H-chromene

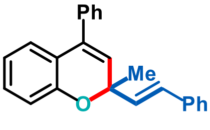

10e, 83%

53.78mg, 83%, colorless liquid

^{1}H NMR (400 MHz, $CDCl_3$) δ 7.45 – 7.33 (m, 7H), 7.29 (t, J = 7.4 Hz, 2H), 7.22 (t, J = 7.8 Hz, 1H), 7.17 (t, J = 7.7 Hz, 1H), 7.02 (d, J = 7.7 Hz, 1H), 6.95 (d, J = 8.1 Hz, 1H), 6.82 (t, J = 6.9 Hz, 1H), 6.68 (d, J = 16.1 Hz, 1H), 6.36 (d, J = 16.1 Hz, 1H), 5.69 (s, 1H), 1.71 (s, 3H).

^{13}C{^{1}H} NMR (100 MHz, $CDCl_3$) δ 153.30, 138.29, 136.58, 136.00, 131.94, 129.51, 129.43, 128.78, 128.53, 128.40, 127.87, 127.75, 126.68, 126.25, 125.79, 122.29, 120.78, 116.82, 77.27, 27.38.

HRMS (ESI-TOF) *m/z* $[M+H]^+$ calcd for $C_{24}H_{21}O$ 325.1587, found 325.1584.

(E)-2-(3,7-dimethylocta-1,6-dien-1-yl)-2,4-dimethyl-2H-chromene

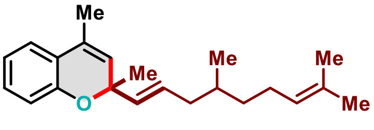

10f, 78%, dr = 1:1
Citronellal

46.18mg, 78%, colorless liquid.

^{1}H NMR (500 MHz, $CDCl_3$) δ 7.16 – 7.08 (m, 2H), 6.86 (t, J = 8.2 Hz, 1H), 6.80 (d, J = 7.9 Hz, 1H), 5.65 – 5.56 (m, 1H), 5.52 (d, J = 16.8 Hz, 1H), 5.40 (s, 1H), 5.10 – 4.96 (m, 1H), 2.04 (s, 3H), 2.01 – 1.76 (m, 4H), 1.68 (s, 3H), 1.58 (s, 3H), 1.50 (s, 3H), 1.41 (m, 1H), 1.27 – 1.15 (m, 1H), 1.10 – 0.98 (m, 1H), 0.75 (d, 6.6 Hz, 3H).

^{13}C{^{1}H} NMR (125 MHz, $CDCl_3$) δ 153.12, 134.19, 134.13, 131.18, 131.16, 129.35, 129.32, 129.02, 128.67, 128.64, 126.88, 125.63, 125.01, 125.00, 123.42, 123.40, 123.38, 120.69, 120.65, 116.44, 77.16, 39.64, 36.67, 36.52, 32.63, 27.52, 27.49, 25.86, 25.65, 25.62, 19.44, 19.32, 18.13, 17.77.

HRMS (ESI-TOF) *m/z* $[M + K]^+$ calcd for $C_{22}H_{30}KO$ 349.1928, found 349.1930.

(E)-3,7-dimethyloct-6-en-1-yl 4-(2-(2,4-dimethyl-2H-chromen-2-yl)vinyl)benzoate

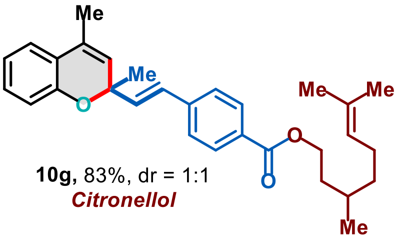

71mg, 83%, colorless liquid

^{1}H NMR (400 MHz, $CDCl_3$) δ 7.94 (d, J = 8.4 Hz, 2H), 7.39 (d, J = 8.3 Hz, 2H), 7.16 (t, J = 9.3 Hz, 2H), 6.90 (t, J = 8.9 Hz, 2H), 6.61 (d, J = 16.1 Hz, 1H), 6.38 (d, J = 16.0 Hz, 1H), 5.48 (s, 1H), 5.10 (t, J = 8.3 Hz, 1H), 4.40 – 4.28 (m, 2H), 2.08 (d, J = 1.4 Hz, 3H), 2.06 – 1.91 (m, 2H), 1.86 – 1.76 (m, 1H), 1.70 – 1.55 (m, 11H), 1.46 – 1.36 (m, 1H), 1.25 (m, J = 16.4, 6.7 Hz, 1H), 0.97 (d, J = 6.5 Hz, 3H).

^{13}C{^{1}H} NMR (100 MHz, $CDCl_3$) δ 166.46, 152.74, 141.16, 135.15, 131.40, 129.79, 129.37, 129.26, 129.13, 127.78, 126.47, 124.58, 124.20, 123.54, 122.76, 120.86, 116.24, 77.26, 63.50, 37.00, 35.51, 29.58, 27.57, 25.74, 25.41, 19.53, 18.09, 17.69.

HRMS (ESI-TOF) *m/z* $[M + H]^+$ calcd for $C_{30}H_{37}O_3$ 445.2737, found 445.2732.

(E)-2-(hept-1-en-1-yl)-4-methyl-2-(4-methylbenzyl)-2H-chromene (10 h)

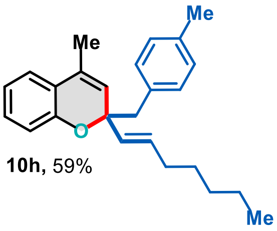

40.8 mg, 59 %, colorless liquid.

^{1}H NMR (500 MHz, $CDCl_3$) δ 7.21 – 7.03 (m, 6H), 6.88 (dd, J = 12.2, 7.6 Hz, 2H), 5.67 – 5.56 (m, 1H), 5.53 – 5.41 (m, 2H), 3.04 (d, J = 13.5 Hz, 1H), 2.99 (d, J = 13.5 Hz, 1H), 2.34 (s, 3H), 2.04 (s, 3H), 1.99 (dd, J = 14.2, 7.1 Hz, 2H), 1.32 – 1.25 (m, 5H), 1.17 (m, 1H), 0.87 (t, J = 7.2 Hz, 3H).

^{13}C{^{1}H} NMR (125 MHz, $CDCl_3$) δ 152.89, 135.69, 133.23, 131.84, 131.64, 130.83, 128.93, 128.73, 128.41, 124.06, 123.30, 123.24, 120.43, 116.32, 79.62, 46.39, 32.25, 31.22, 28.72, 22.49, 21.07, 18.09, 14.02.

HRMS (ESI-TOF) *m/z* $[M + H]^+$ calcd for $C_{25}H_{31}O$ 347.2369, found 347.2379.

(E)-2-isobutyl-4-methyl-2-(4-methylstyryl)-2H-chromene(10i)

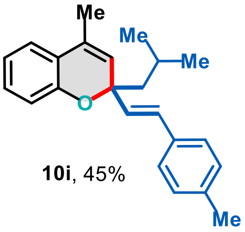

10i, 45%

28.62 mg, 45 %, colorless liquid.

^{1}H NMR (500 MHz, CDCl$_3$) δ 7.25 (d, J = 8.1 Hz, 2H), 7.16 (d, J = 7.7 Hz, 2H), 7.10 (d, J = 8.0 Hz, 2H), 6.91 – 6.86 (m, 2H), 6.55 (d, J = 16.0 Hz, 1H), 6.14 (d, J = 16.0 Hz, 1H), 5.47 (s, 1H), 2.33 (s, 3H), 2.09 (s, 3H), 2.01 (m, 1H), 1.75 (d, J = 6.1 Hz, 2H), 1.00 (d, J = 6.7 Hz, 3H), 0.97 (d, J = 6.7 Hz, 3H).

^{13}C{^{1}H} NMR (125 MHz, CDCl$_3$) δ 152.96, 142.61, 137.21, 134.13, 131.60, 129.14, 129.02, 128.49, 128.45, 126.41, 124.29, 123.36, 120.39, 116.13, 80.35, 49.23, 24.43, 24.37, 24.20, 21.14, 18.13.

HRMS (ESI-TOF) *m/z* [M + H]$^+$ calcd for $C_{23}H_{27}O$ 319.2056, found 319.2068.

Functionalization:

To a solution of **7g** (50 mg, 0.10mmol) in MeOH (2 mL) was added Pd/C (10 mol%) and H_2 gas was bubbled from a balloon at room temperature. After 10 h, mixture was diluted with DCM and filtered through a pad of celite bad. The combined organic phase was dried with Na_2SO_4. After removal of the solvent under reduced pressure, the residue was purified by column chromatography (silica gel mesh100-200; hexane: ethyl acetate; 80:20) to give the product **8** (41 mg, 82% yield).

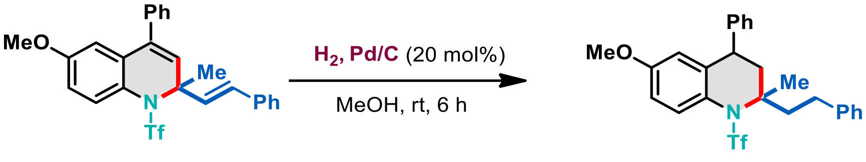

^{1}H NMR (400 MHz, CDCl$_3$) δ 7.39 (d, J = 8.9 Hz, 1H), 7.33 (t, J = 7.4 Hz, 2H), 7.28 – 7.23 (m, 2H), 7.22 – 7.15 (m, 4H), 6.92 (d, J = 7.4 Hz, 2H), 6.82 (dd, J = 9.0, 2.8 Hz, 1H), 6.45 (d, J = 2.6 Hz, 1H), 4.13 (t, J = 9.8 Hz, 1H), 3.69 (s, 3H), 2.76 (td, J = 12.9, 5.1 Hz, 1H), 2.58 (td, J = 12.8, 3.8 Hz, 1H), 2.49 – 2.38 (m, 1H), 2.28 (dd, J = 14.5, 8.7 Hz, 1H), 1.97 (t, J = 15.1 Hz, 1H), 1.82 (s, 3H), 1.62 – 1.58 (m, 1H).

^{13}C{^{1}H} NMR (100 MHz, CDCl$_3$) δ 158.34, 144.47, 141.24, 136.38, 129.08, 128.97, 128.48, 128.36, 128.24, 126.86, 126.07, 121.17-119.02 (q, J = 326.0 Hz) 114.71, 112.89, 66.01, 55.38, 46.38, 42.22, 41.20, 30.02, 25.08.

HRMS (ESI-TOF) *m/z* [M + Na]$^+$ calcd for $C_{26}H_{26}F_3NNaO_3S$ 512.1478 found 512.1470.

(E)-2,4-dimethyl-2-(2-(phenylethynyl)styryl)-1-((trifluoromethyl)sulfonyl)-1,2-dihydroquinoline

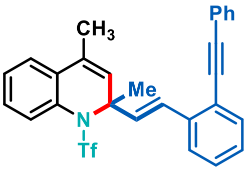

13, 75%

73.5 mg, 75 %, colorless liquid.

^{1}H NMR (400 MHz, $CDCl_3$) δ 7.55 (dd, *J* = 7.8, 1.8 Hz, 2H), 7.49 (d, *J* = 7.0 Hz, 1H), 7.43 (m, 4H), 7.37 (d, *J* = 7.9 Hz, 1H), 7.25 – 7.15 (m, 5H), 7.02 (d, *J* = 16.1 Hz, 1H), 6.20 (d, *J* = 16.1 Hz, 1H), 5.94 (s, 1H), 2.13 (s, 3H), 1.88 (s, 3H).

^{13}C{^{1}H} NMR (100 MHz, $CDCl_3$) δ 137.73, 134.35, 133.13, 132.82, 132.44, 132.21, 131.58, 130.68, 128.67, 128.64, 128.59, 128.19, 128.06, 127.98, 127.80, 127.72, 125.28, 123.39, 123.23, 122.25, 122.08(q, *J* = 325.8 Hz) 94.23, 87.62, 64.59, 26.68, 17.98.

HRMS (ESI-TOF) *m/z* $[M + K]^+$ calcd for $C_{28}H_{22}F_3KNO_2S$ 532.0955, found 532.0944.

(E)-2,4-dimethyl-2-(2-(phenylethynyl)styryl)-2H-chromene

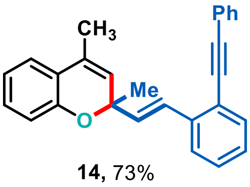

14, 73%

52.8 mg, 73 %, colorless liquid.

^{1}H NMR (500 MHz, $CDCl_3$) δ 7.55 (m, 3H), 7.51 (d, *J* = 7.8 Hz, 1H), 7.41 (m, 3H), 7.30 – 7.28 (m, 1H), 7.23 (m, 2H), 7.16 (d, *J* = 7.5 Hz, 1H), 7.10 (t, *J* = 7.7 Hz, 1H), 6.92 – 6.85 (m, 2H), 6.42 (d, *J* = 14.2 Hz, 1H), 5.55 (s, 1H), 2.08 (s, 3H), 1.69 (s, 3H).

^{13}C{^{1}H} NMR (125 MHz, $CDCl_3$) δ 153.08, 138.44, 134.37, 133.08, 132.59, 131.76, 129.37, 129.25, 128.60, 128.55, 127.50, 127.29, 125.32, 124.71, 123.68, 123.62, 123.05, 122.38, 120.92, 116.49, 94.36, 88.05, 77.73, 27.95, 18.30.

HRMS (ESI-TOF) *m/z* $[M + H]^+$ calcd for $C_{27}H_{23}O$ 363.1743, found 363.1755.

Mechanistic Study:

A sealed tube containing $[Cp^*RhCl_2]_2$ (2.5 mol %), NaOAc (30 mol%) was evacuated and purged with nitrogen gas three times. Then, *o*-alkenylanilides **1** (0.20 mmol) and allenic acetate **2** (0.30 mmol) in DCE (2 ml) were added via syringe under nitrogen atmosphere and the reaction mixture was allowed to stir at rt for 24 h. Then, the mixture diluted with CH_2Cl_2 (10 mL). The mixture was filtered through a Celite pad and washed with CH_2Cl_2 (3 × 10 mL). The filtrate was concentrated under reduced pressure.

The residue was purified by silica gel column chromatography using hexane/ethyl acetate as eluent to afford recovered substrate **1a**.

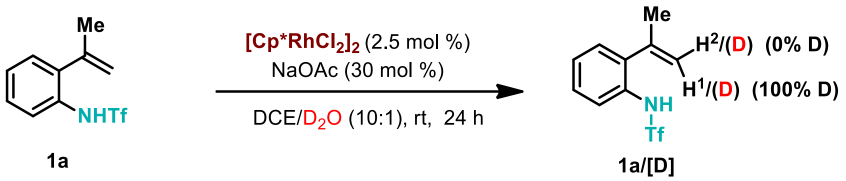

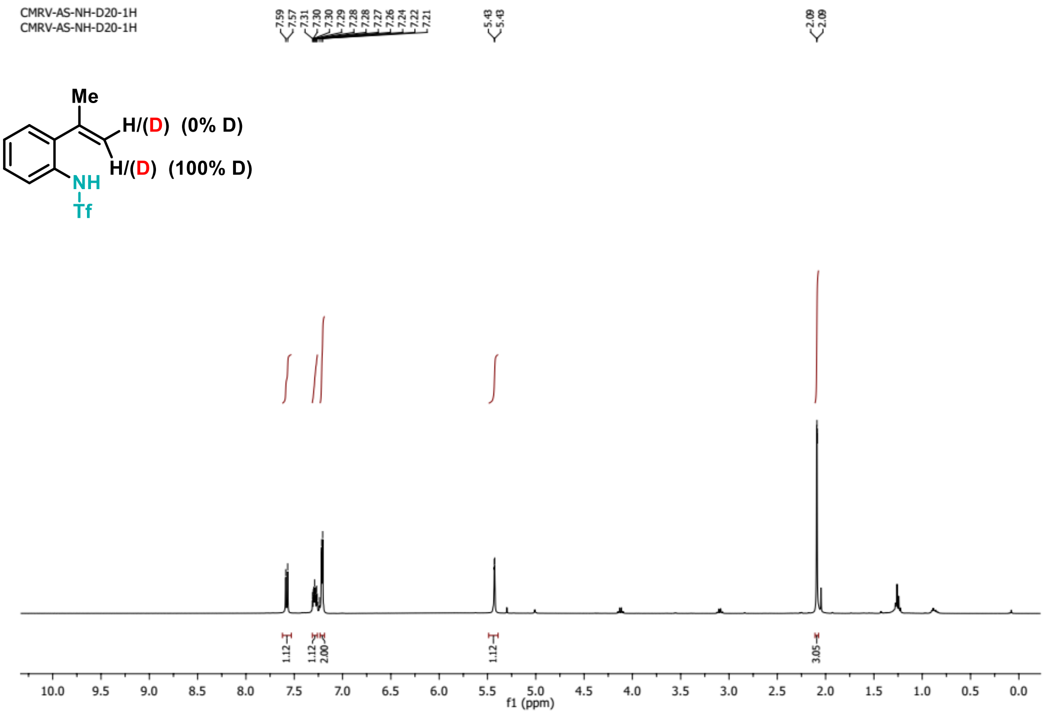

A sealed tube containing [Cp*RhCl$_2$]$_2$ (2.5 mol %), NaOAc (30 mol%) was evacuated and purged with nitrogen gas three times. Then, *o*-alkenylanilides **1** (0.20 mmol) and allenic acetate **2** (0.30 mmol) in CH$_3$CN (2 ml) were added via syringe under nitrogen atmosphere and the reaction mixture was allowed to stir at rt for 24 h. Then, the mixture diluted with CH$_2$Cl$_2$ (10 mL). The mixture was filtered through a Celite pad and washed with CH$_2$Cl$_2$ (3 × 10 mL). The filtrate was concentrated under reduced pressure. The residue was purified by silica gel column chromatography using hexane/ethyl acetate as eluent to afford annulated product **3a**.

b) H/D exchange study

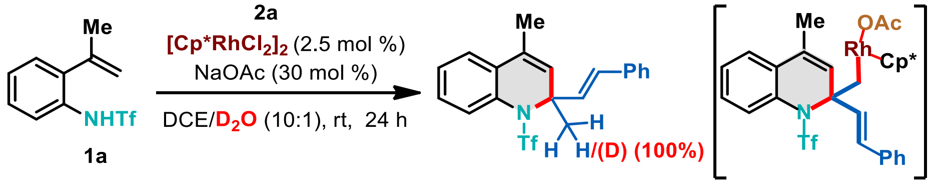

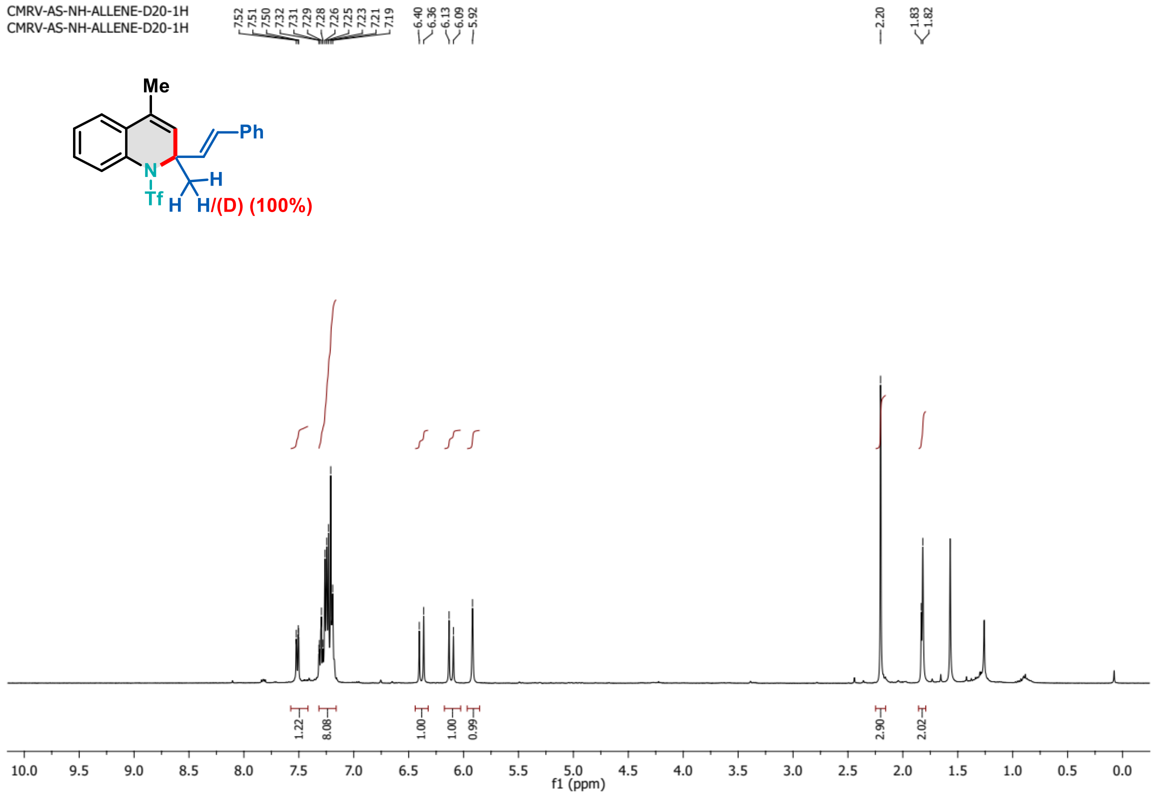

Competitive experiment: A sealed tube containing [Cp*RhCl$_2$]$_2$ (2.5 mol %), NaOAc (30 mol%) was evacuated and purged with nitrogen gas three times. Then, *o*-alkenylanilides **1g** (0.10 mmol) and **1l** (0.10 mmol) and allenic acetate **2a** (0.12 mmol) in CH$_3$CN (2 ml) were added via syringe under nitrogen atmosphere and the reaction mixture was allowed to stir at rt for 1 h. Then, the mixture diluted with CH$_2$Cl$_2$ (10 mL). The mixture was filtered through a Celite pad and washed with CH$_2$Cl$_2$ (3 × 10 mL). The filtrate was concentrated under reduced pressure and crude NMR of mixture on recorded.

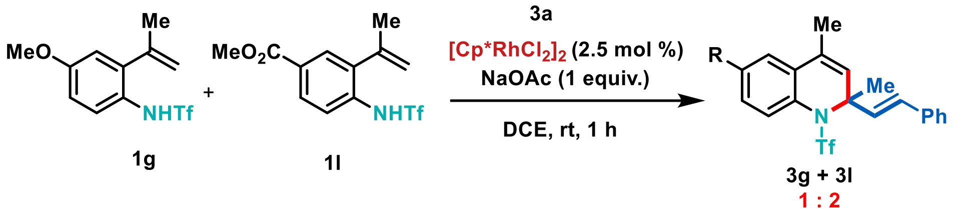

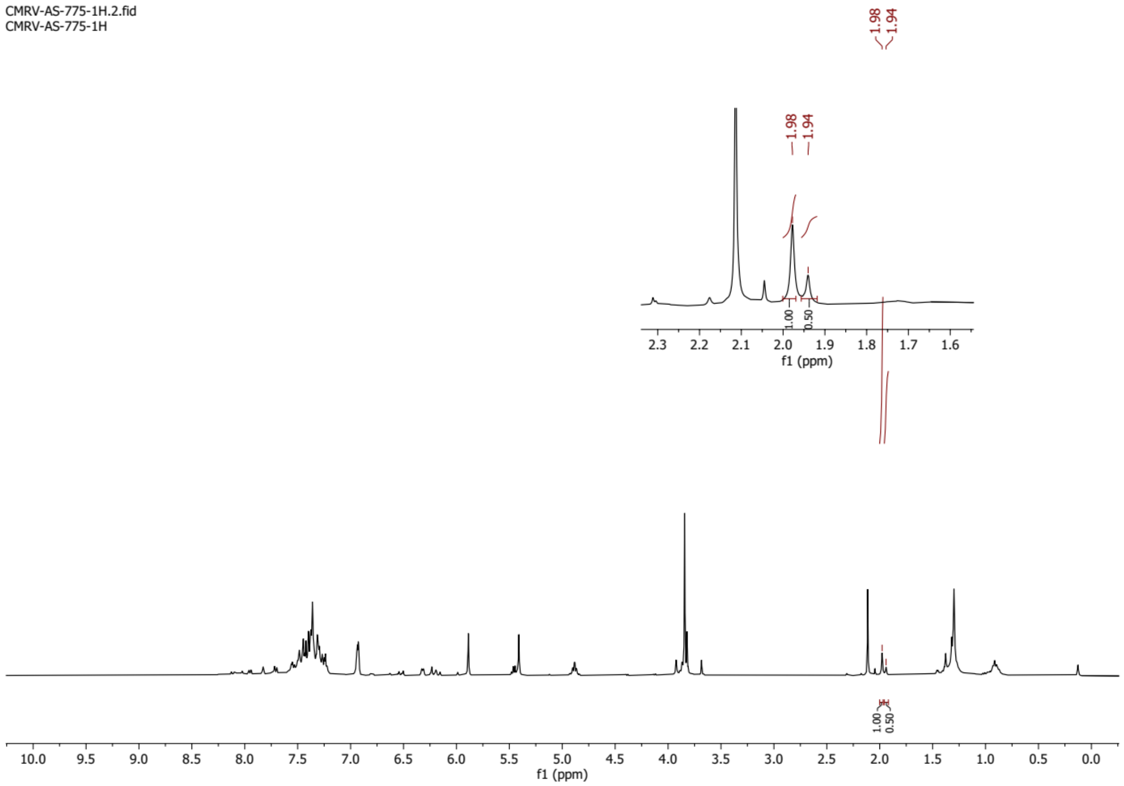

Refereneces:

1. (a) Font, M.; Cendon, B.; Seoane, A.; Mascarenas, J. L.; Gulias, M. *Angew. Chem. Int. Ed.* **2018**, *57*, 8255 –8259. (b) Andres Seoane, Cezar Comanescu, Noelia Casanova, Rebeca Garcia-Fandino, Xabier Diz, Jose L. Mascarenas, Moises Gulias. *Angew. Chem. Int. Ed.* **2019**, *58*, 1700 –1704.
2. Chen, P.; Nan, J.; Hu, Y.; Ma, Q.; Ma. Y. *Org. Lett.* **2019**, *21*, 4812-4815.
3. Shukla, R. K, Nair, A. M.; Khan, S.; Volla, C. M. R. Cobalt-Catalyzed C8-Dienylation of Quinoline-N-Oxides. *Angew.Chem.Int. Ed.* **2020**, *59*, 17042 –17048.

^{1}H and ^{13}C spectra

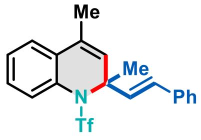

7a, 91%

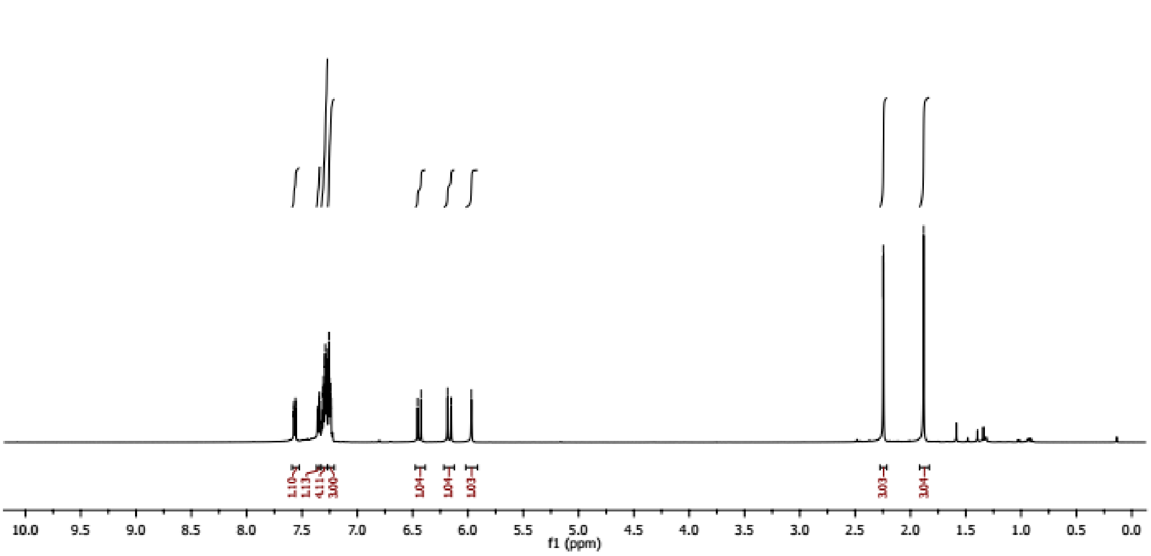
cmrv-as-412-1h
cmrv-as-412-1h
7.58 7.57 7.56 7.56 7.36 7.36 7.35 7.34 7.32 7.32 7.31 7.30 7.29 7.29 7.28 7.28 7.27 7.26 7.25 7.24 7.24 6.46 6.42 6.18 6.15 5.97
2.25 2.24
1.88
1.10 1.13 4.11 3.00 1.04 1.04 1.03 3.03 3.04
10.0 9.5 9.0 8.5 8.0 7.5 7.0 6.5 6.0 5.5 5.0 4.5 4.0 3.5 3.0 2.5 2.0 1.5 1.0 0.5 0.0
f1 (ppm)

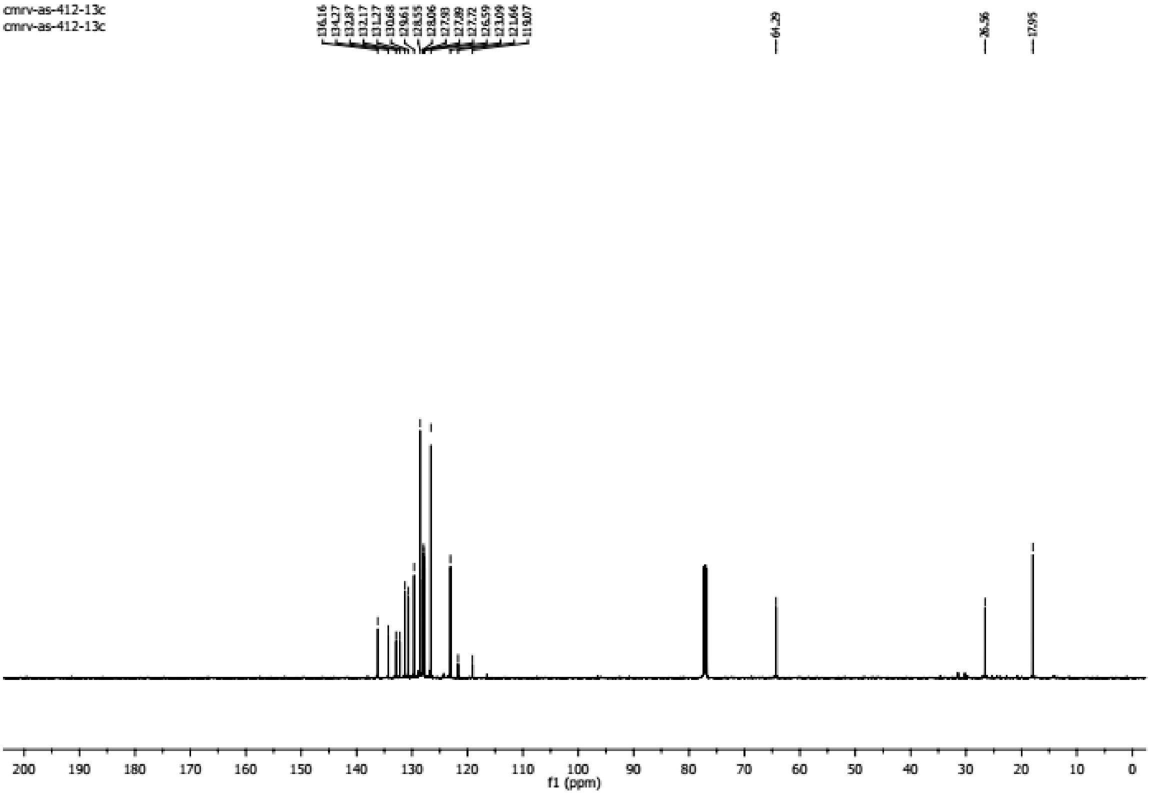
cmrv-as-412-13c
cmrv-as-412-13c
136.16 134.27 132.87 132.17 131.27 130.68 129.61 128.55 128.06 127.93 127.89 127.72 126.59 123.09 121.66 119.07
64.29
26.56
17.95
200 190 180 170 160 150 140 130 120 110 100 90 80 70 60 50 40 30 20 10 0
f1 (ppm)

CMRV-AS-493-1H
CMRV-AS-493-1H

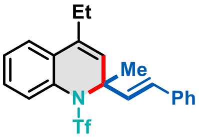

7b, 81%

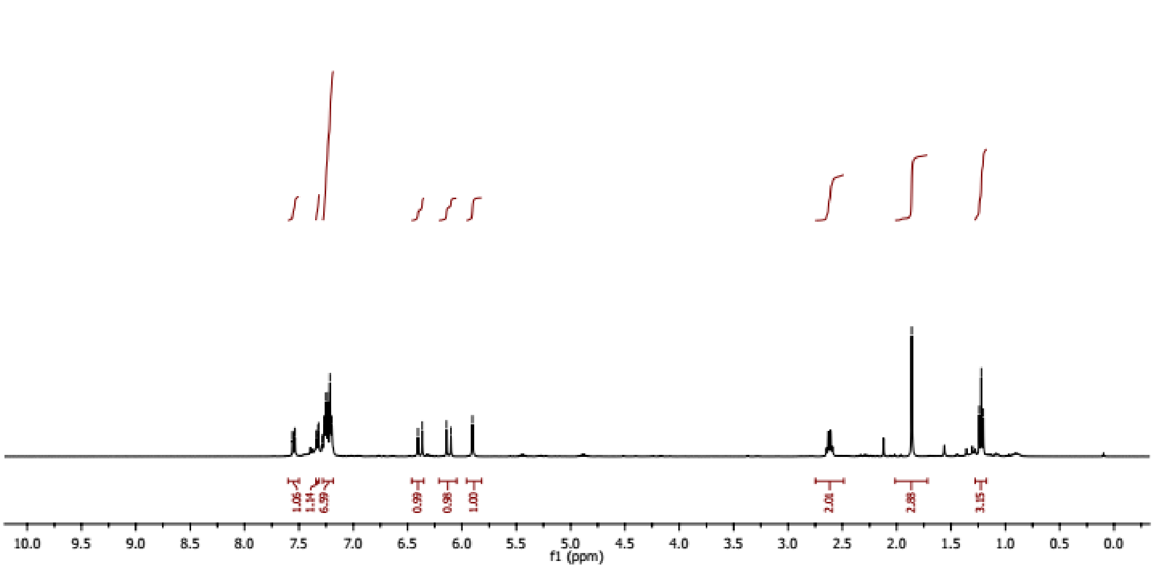

CMRV-AS-493-13C
CMRV-AS-493-13C

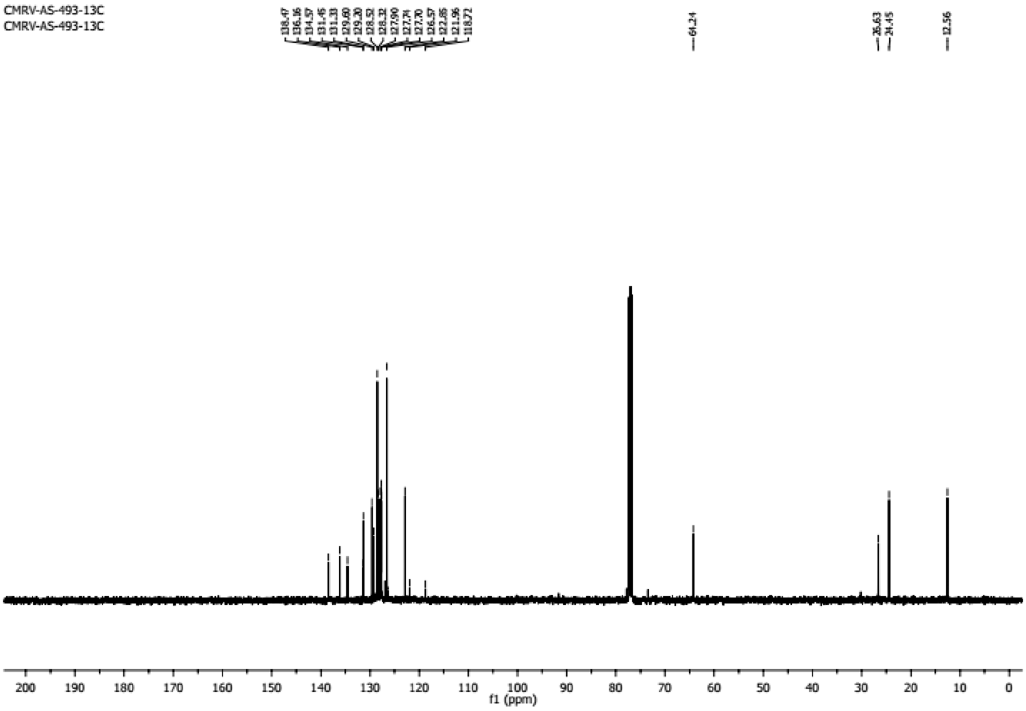

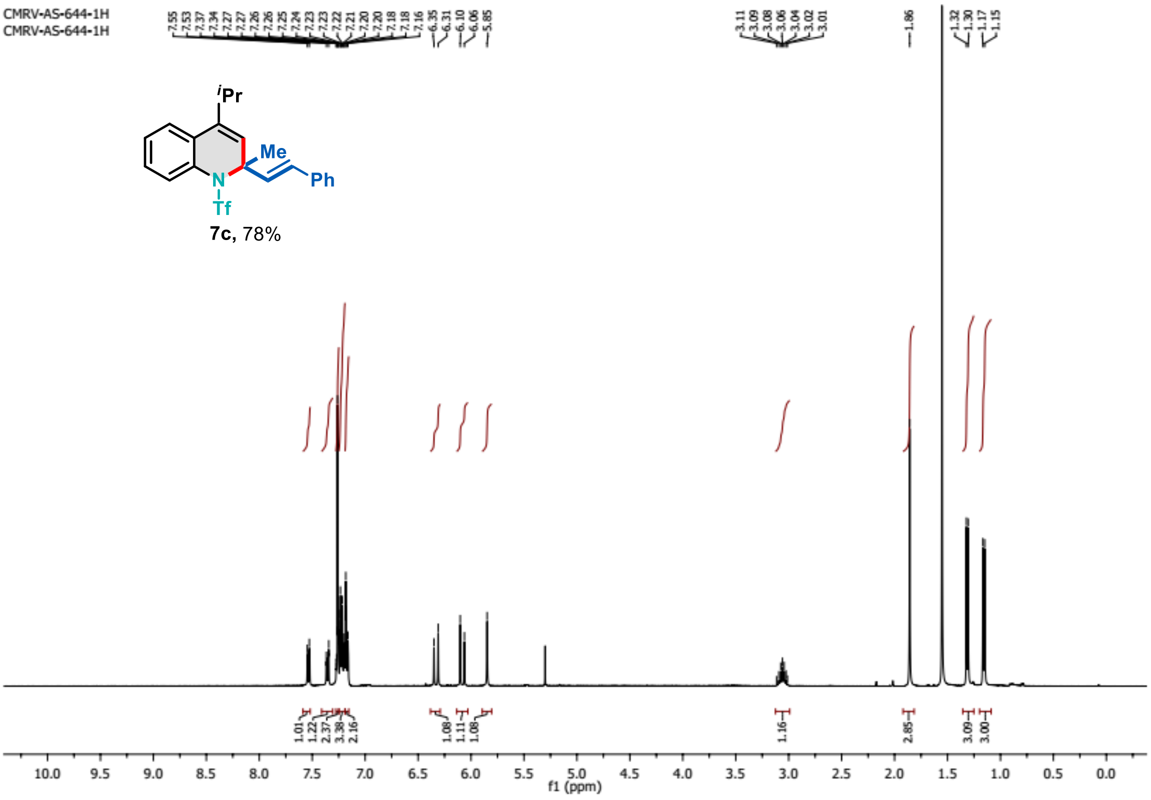
CMRV-AS-644-1H
CMRV-AS-644-1H
7.55 7.53 7.37 7.34 7.27 7.27 7.26 7.26 7.25 7.24 7.23 7.23 7.22 7.21 7.20 7.20 7.18 7.18 7.16 6.35 6.31 6.10 6.06 5.85
3.11 3.09 3.08 3.06 3.04 3.02 3.01
1.86
1.32 1.30 1.17 1.15
iPr
Me
N
Ph
Tf
7c, 78%
1.01 1.22 2.37 3.38 2.16 1.08 1.11 1.08 1.16 2.85 3.09 3.00
10.0 9.5 9.0 8.5 8.0 7.5 7.0 6.5 6.0 5.5 5.0 4.5 4.0 3.5 3.0 2.5 2.0 1.5 1.0 0.5 0.0
f1 (ppm)

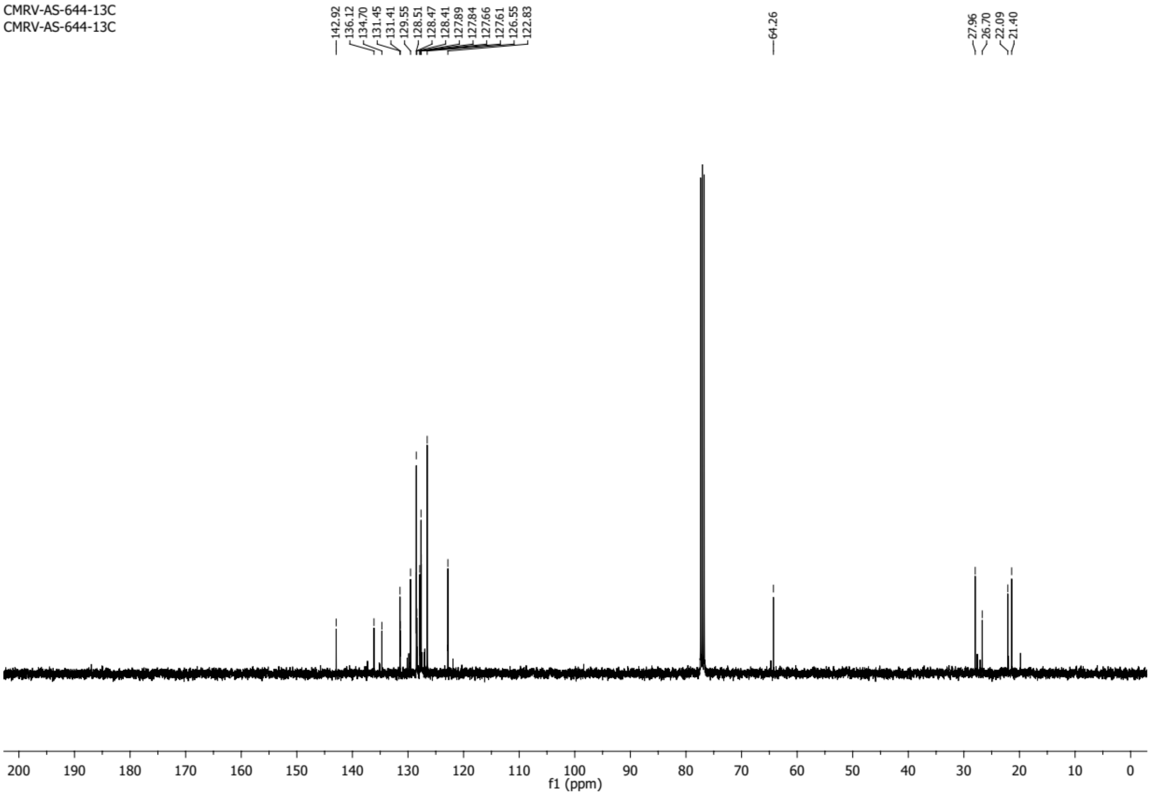
CMRV-AS-644-13C
CMRV-AS-644-13C
142.92 136.12 134.70 131.45 131.41 129.55 128.51 128.47 128.41 127.89 127.84 127.66 127.61 126.55 122.83
64.26
27.96 26.70 22.09 21.40
200 190 180 170 160 150 140 130 120 110 100 90 80 70 60 50 40 30 20 10 0
f1 (ppm)

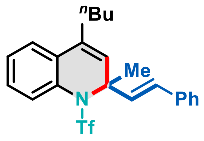

nBu
Me
Ph
N
Tf

7d, 82%

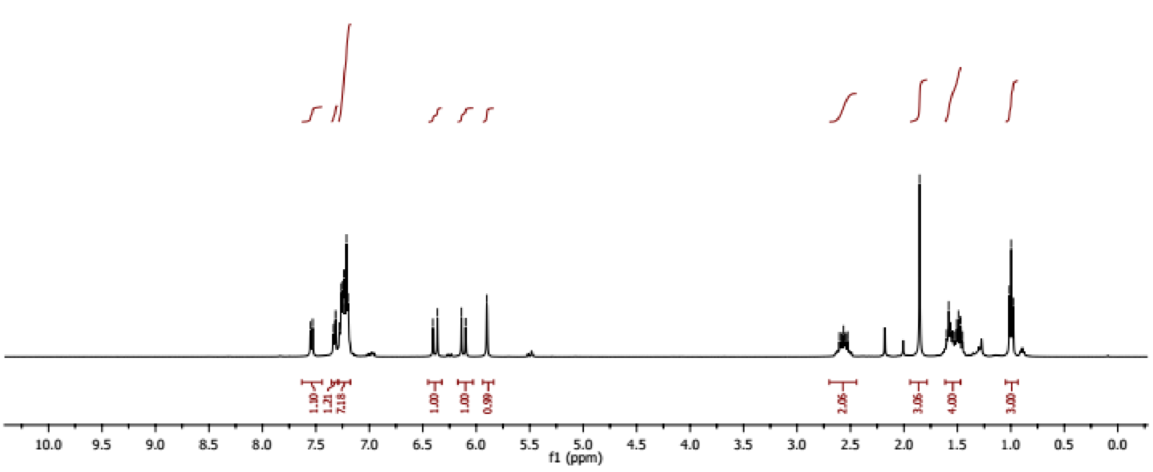

CMRV-AS-643-1H
CMRV-AS-643-1H
f1 (ppm)

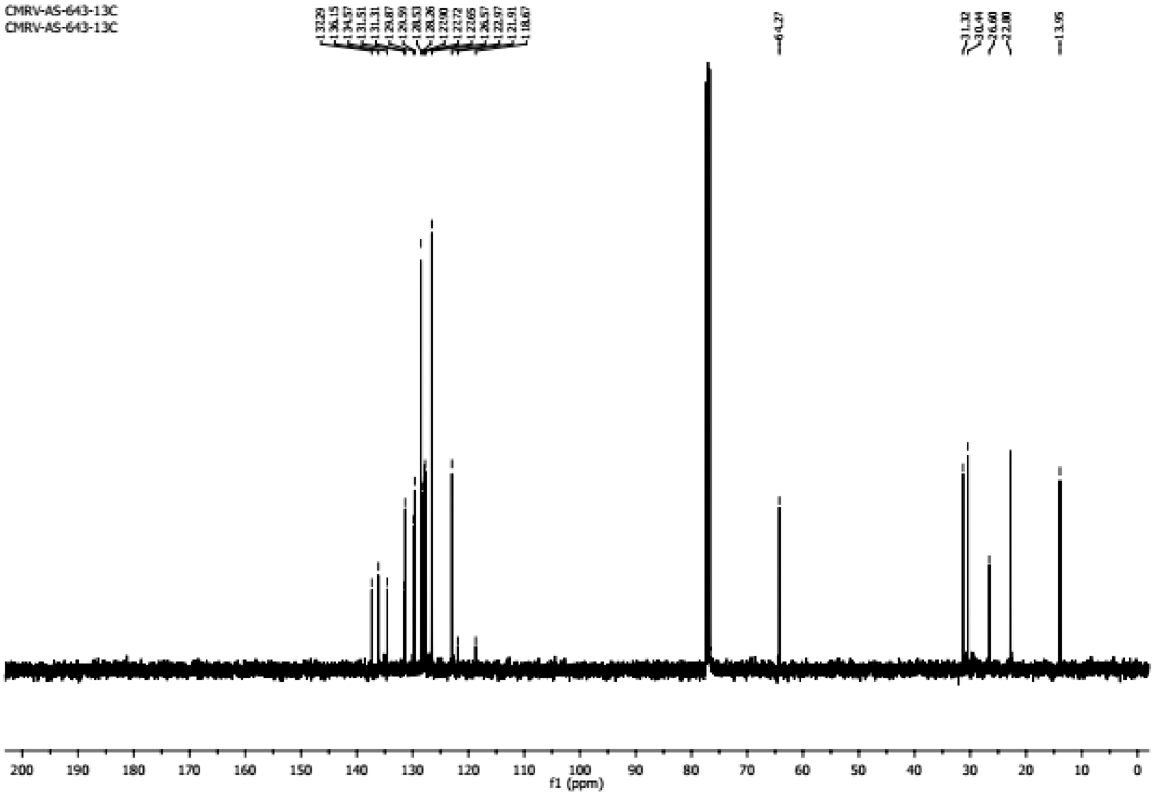

CMRV-AS-643-13C
CMRV-AS-643-13C
f1 (ppm)

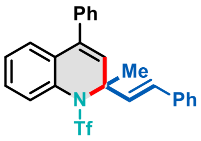

7e, 85%

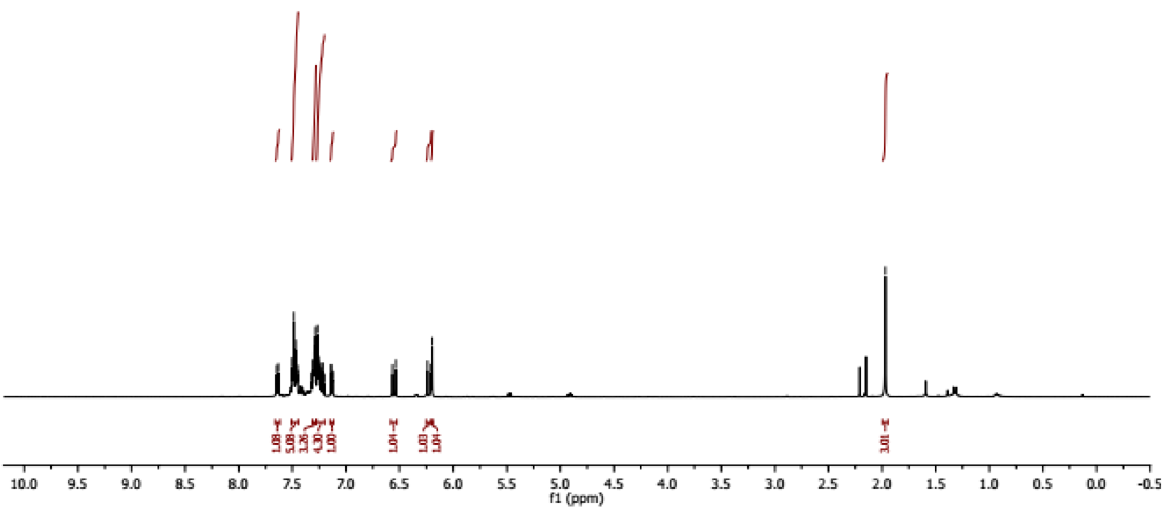

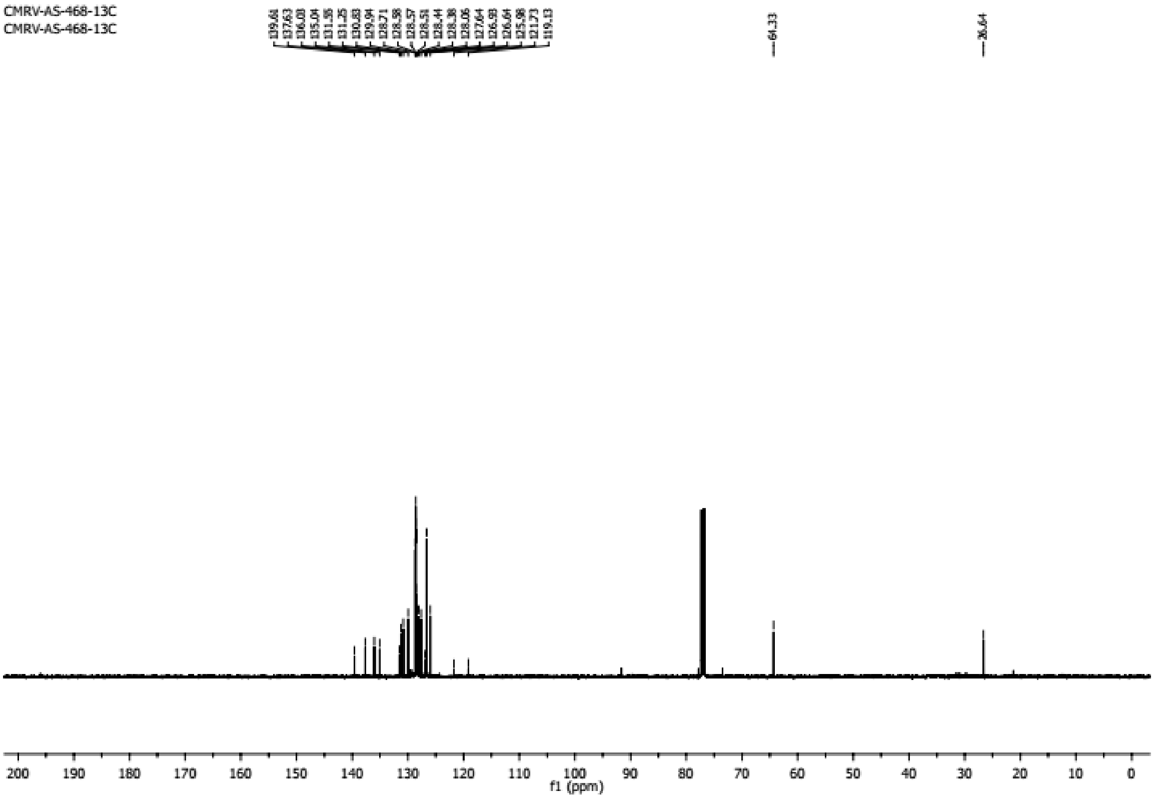

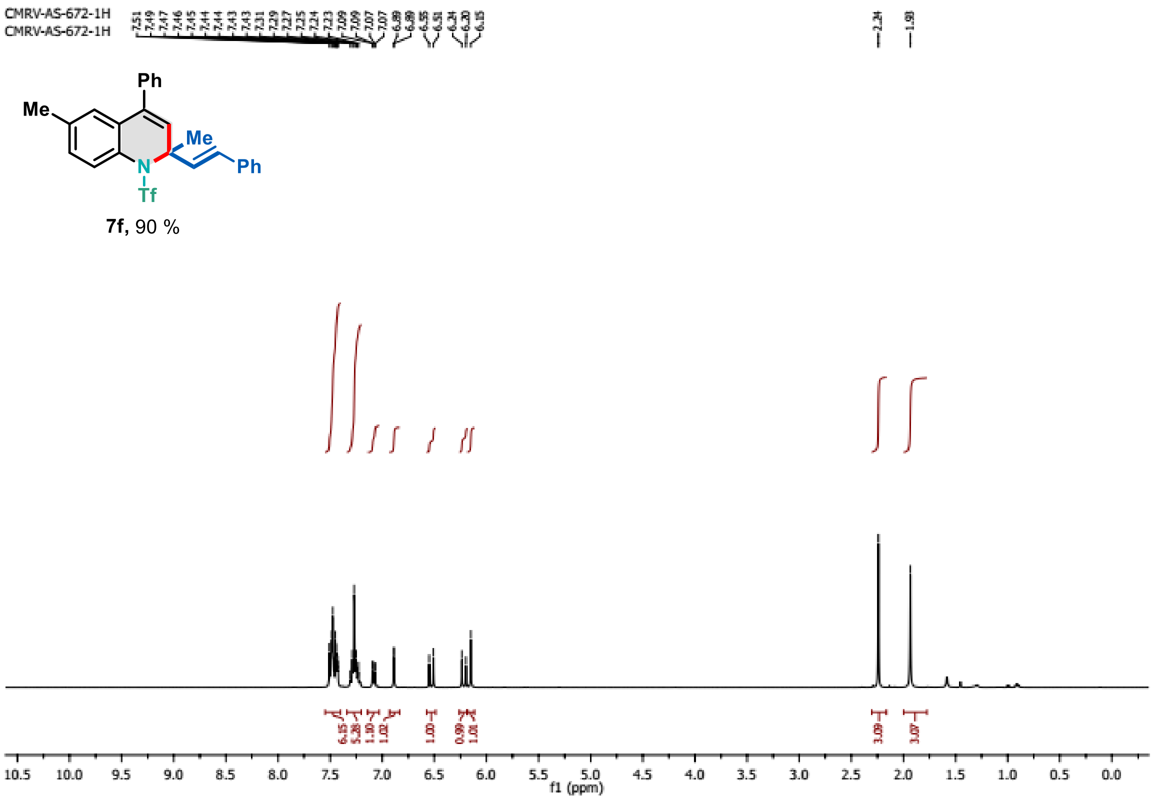
CMRV-AS-672-1H
CMRV-AS-672-1H
7.51 7.49 7.47 7.46 7.45 7.44 7.44 7.43 7.31 7.29 7.27 7.25 7.24 7.23 7.09 7.09 7.07 7.07 6.89 6.89 6.55 6.51 6.24 6.20 6.15
2.24
1.93
Ph
Me
Me
Ph
N
Tf
7f, 90 %
6.15 5.28 1.10 1.02 1.00 0.99 1.01 3.09 3.07
10.5 10.0 9.5 9.0 8.5 8.0 7.5 7.0 6.5 6.0 5.5 5.0 4.5 4.0 3.5 3.0 2.5 2.0 1.5 1.0 0.5 0.0
f1 (ppm)

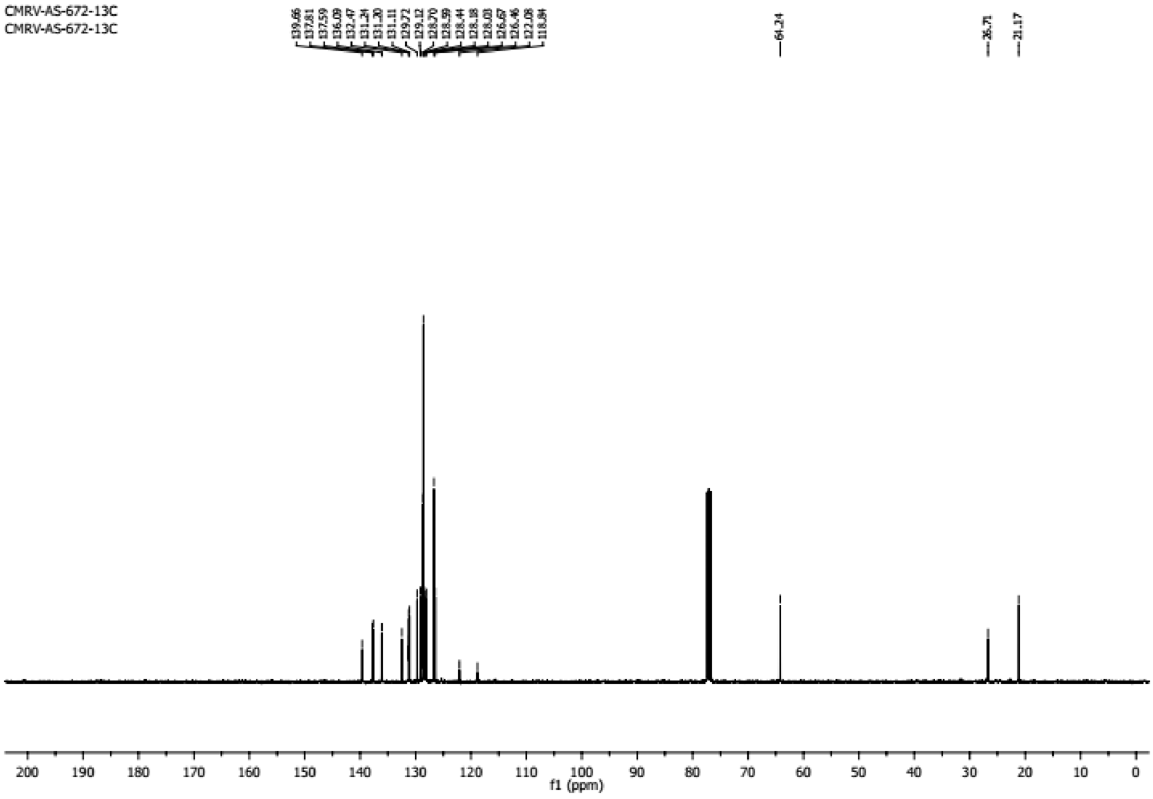
CMRV-AS-672-13C
CMRV-AS-672-13C
139.66 137.81 137.59 136.09 132.47 131.24 131.20 131.11 129.72 129.12 128.70 128.59 128.44 128.18 128.03 126.67 126.46 122.08 118.84
64.24
26.71
21.17
200 190 180 170 160 150 140 130 120 110 100 90 80 70 60 50 40 30 20 10 0
f1 (ppm)

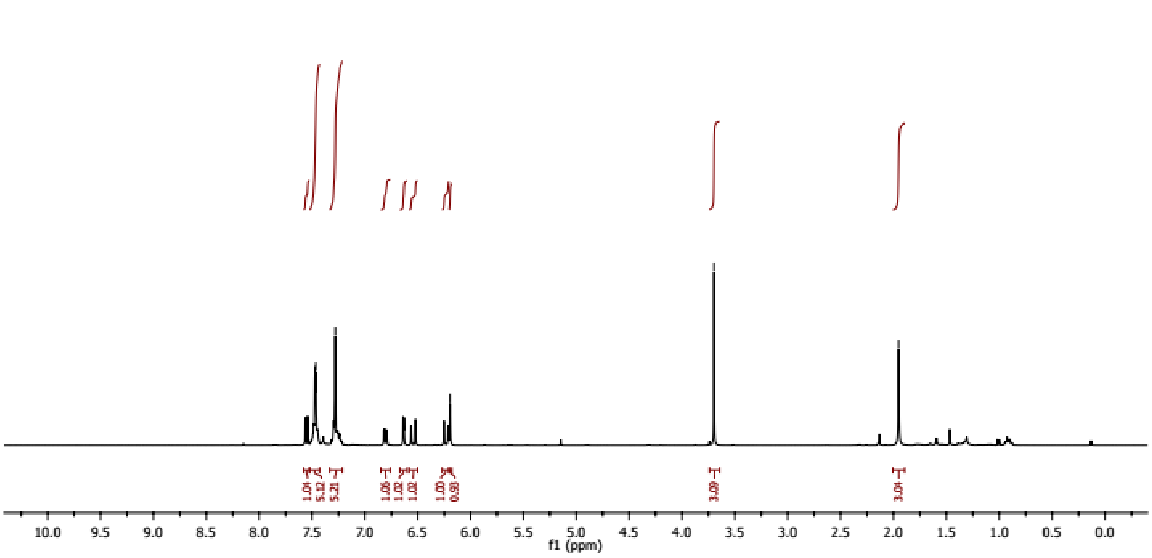
CMRV-AS-679-1H
CMRV-AS-679-1H
7.47
7.46
7.28
6.20
3.70
1.95
1.04
5.12
5.21
1.06
1.02
1.02
1.00
0.93
3.09
3.04
f1 (ppm)

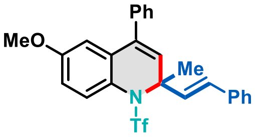
MeO
Ph
Me
N
Tf
Ph

7g, 85%

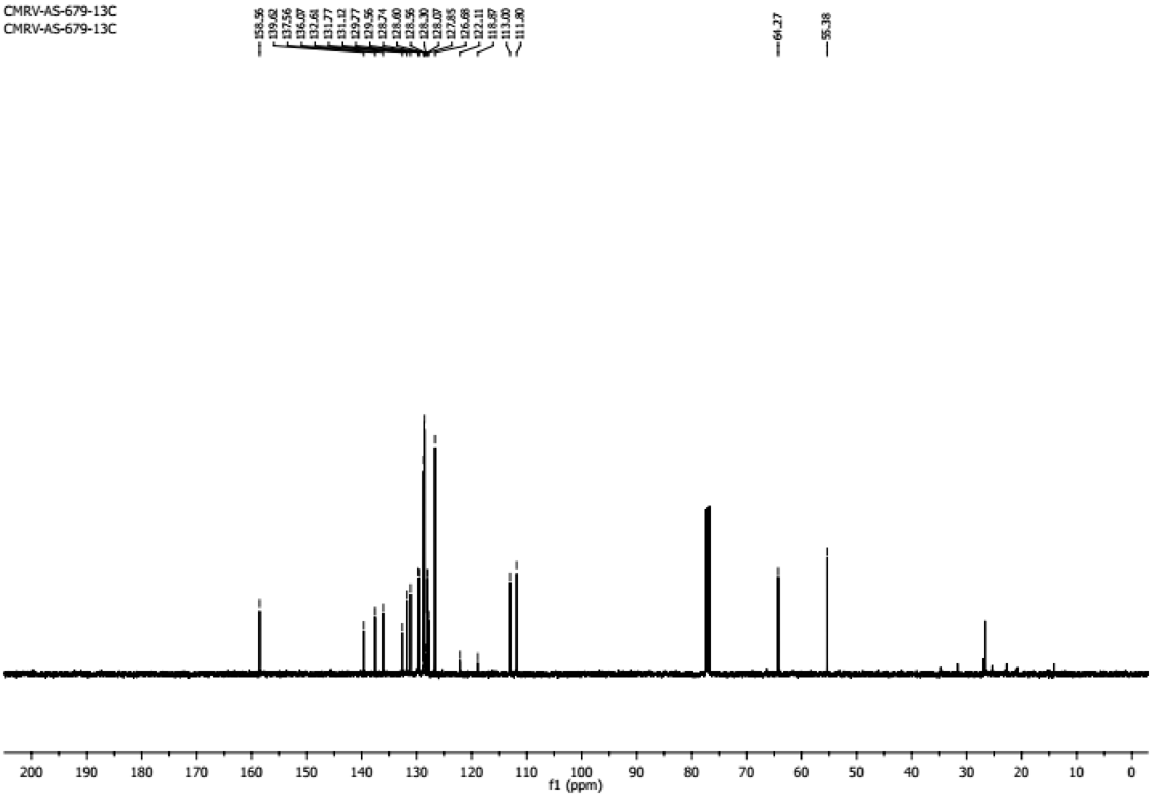
CMRV-AS-679-13C
CMRV-AS-679-13C
158.56
139.62
137.56
136.07
132.61
131.77
131.12
129.77
129.56
128.74
128.60
128.56
128.30
128.07
127.85
126.68
122.11
118.87
113.00
111.80
64.27
55.38
f1 (ppm)

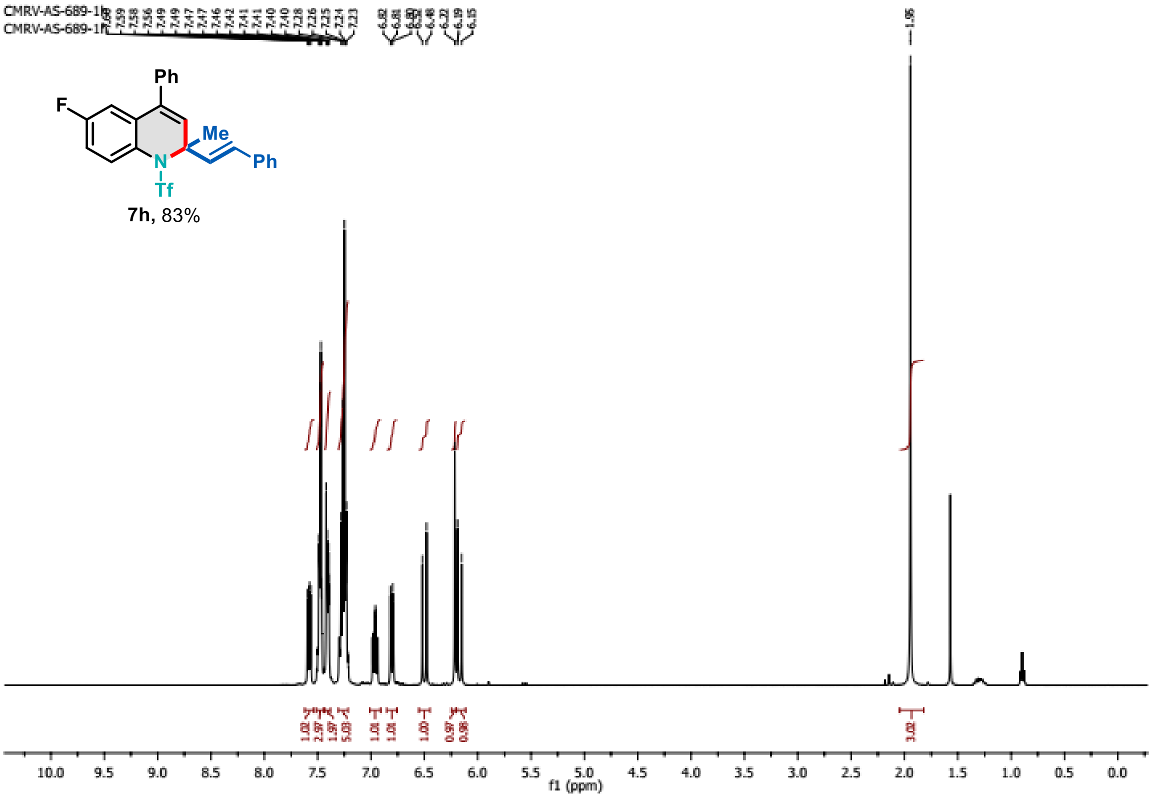
Ph
F
Me
N
Ph
Tf
7h, 83%
f1 (ppm)

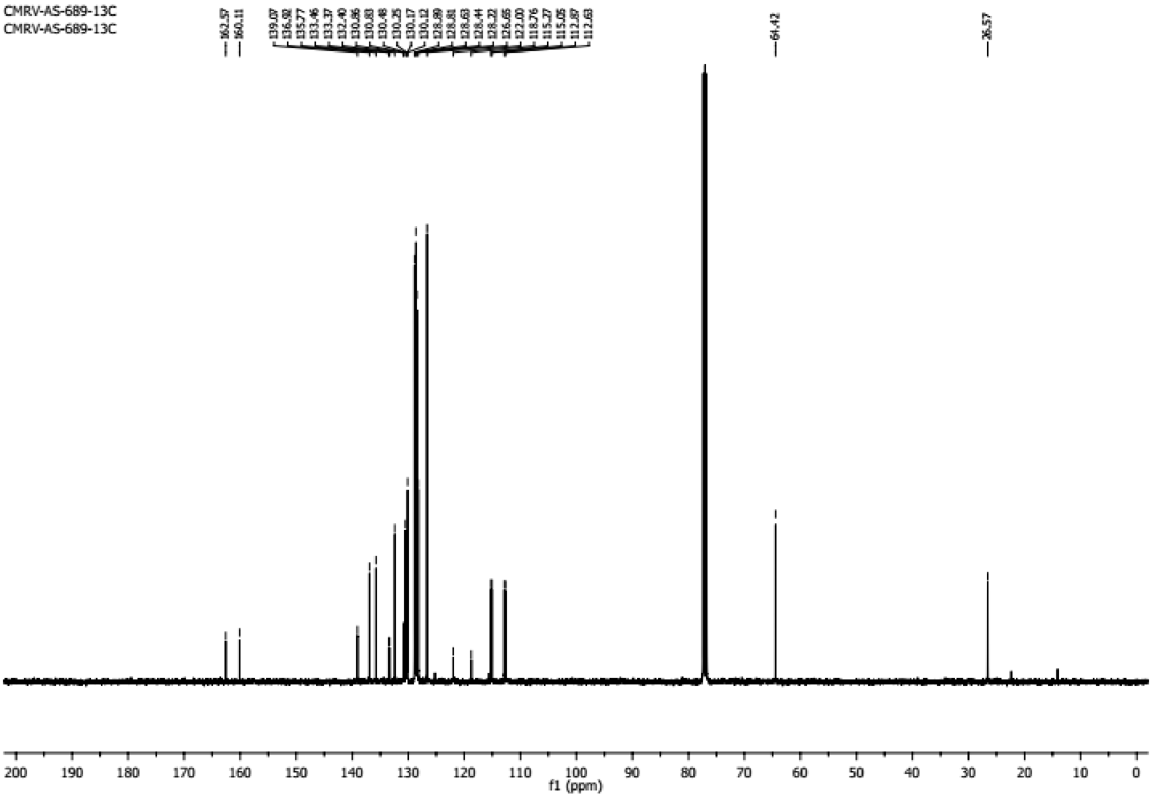
CMRV-AS-689-13C
CMRV-AS-689-13C
f1 (ppm)

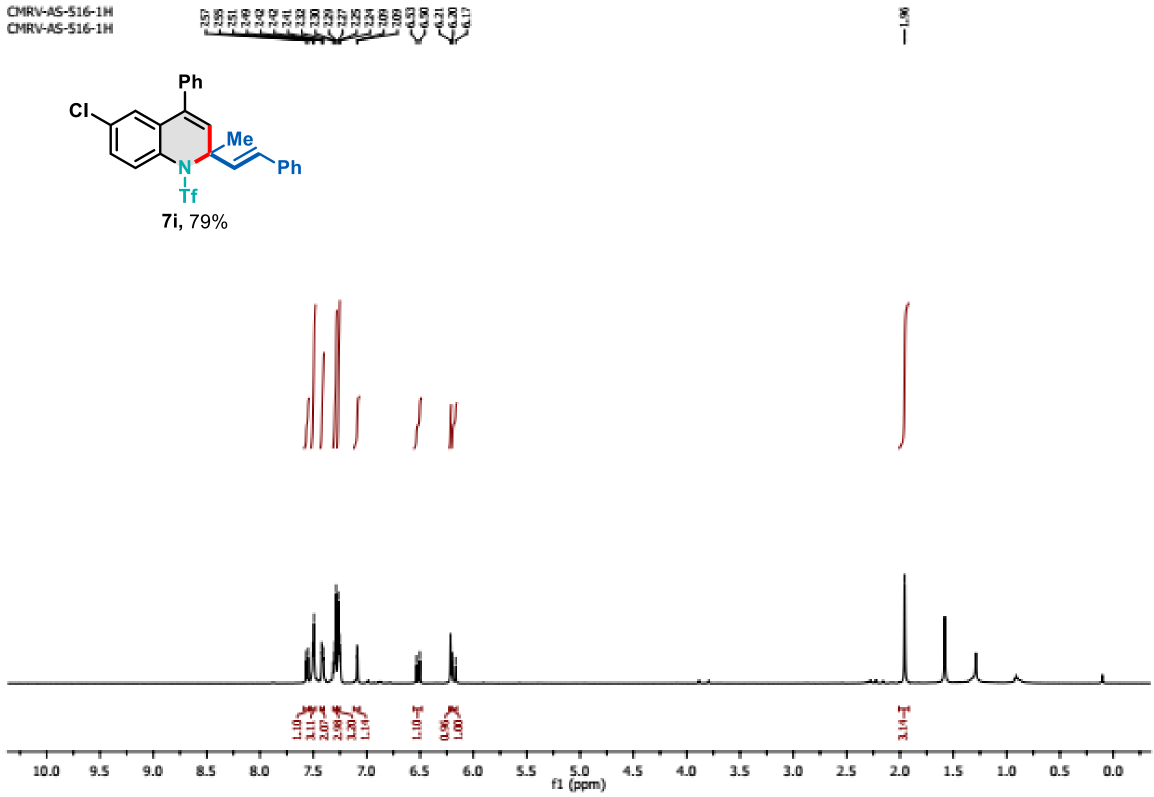
CMRV-AS-516-1H
CMRV-AS-516-1H
1.96
Ph
Cl
Me
Ph
N
Tf
7i, 79%
f1 (ppm)

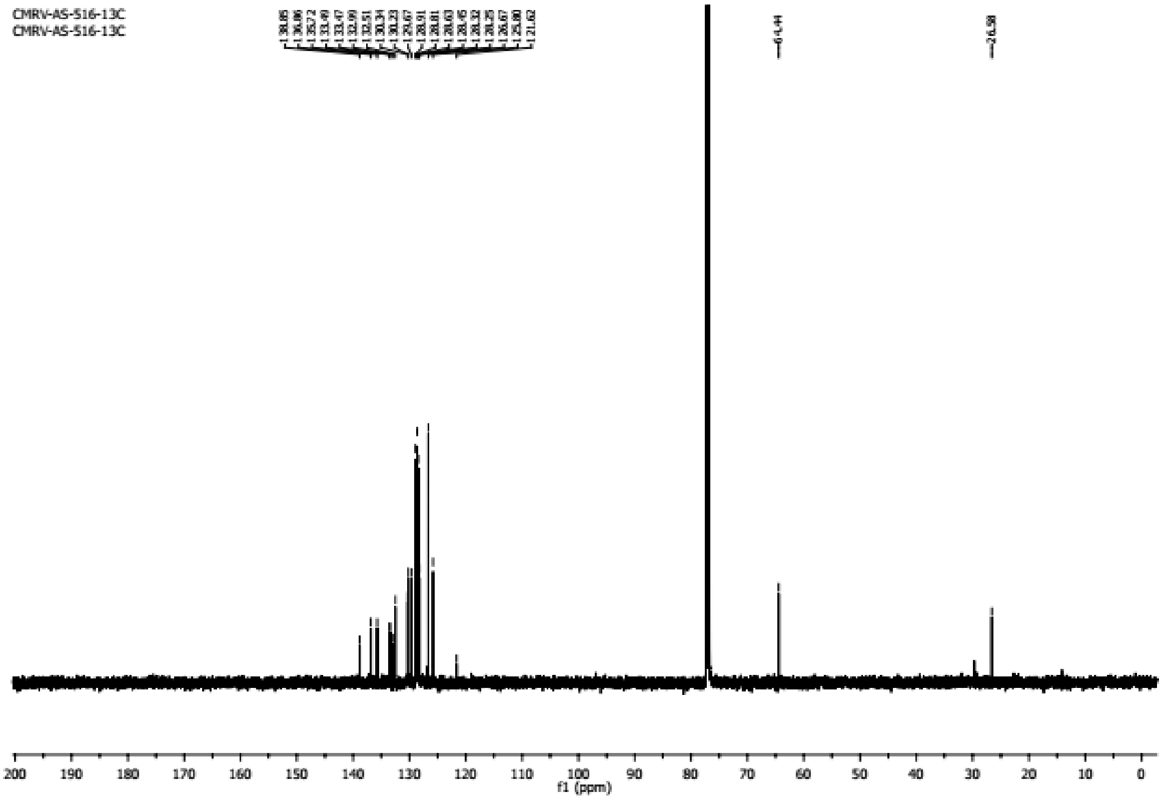
CMRV-AS-516-13C
CMRV-AS-516-13C
64.44
26.58
f1 (ppm)

CMRV-AS-647-1H
CMRV-AS-647-1H

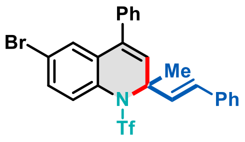

7j, 81%

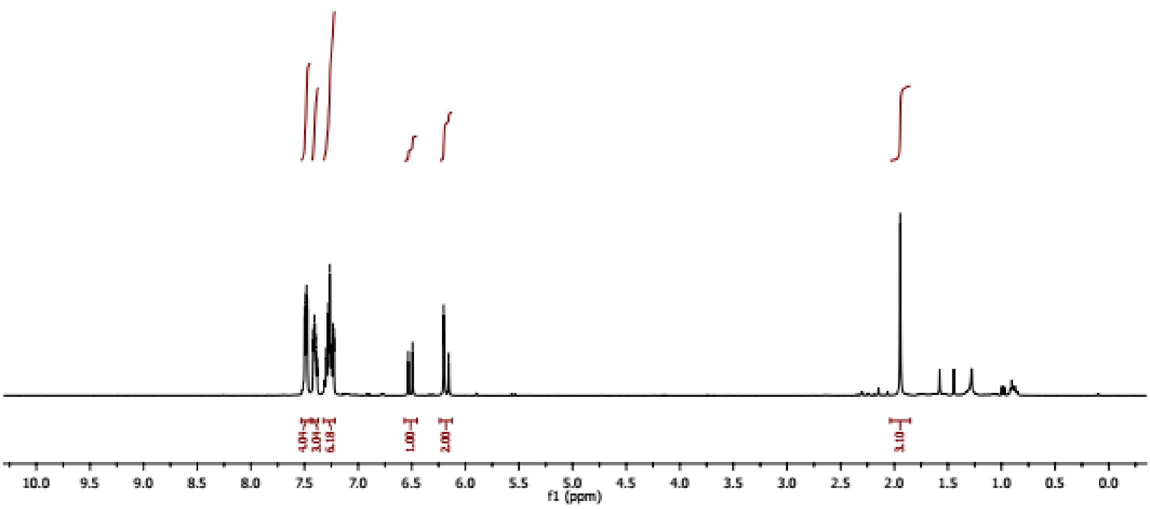

CMRV-AS-647-13C
CMRV-AS-647-13C

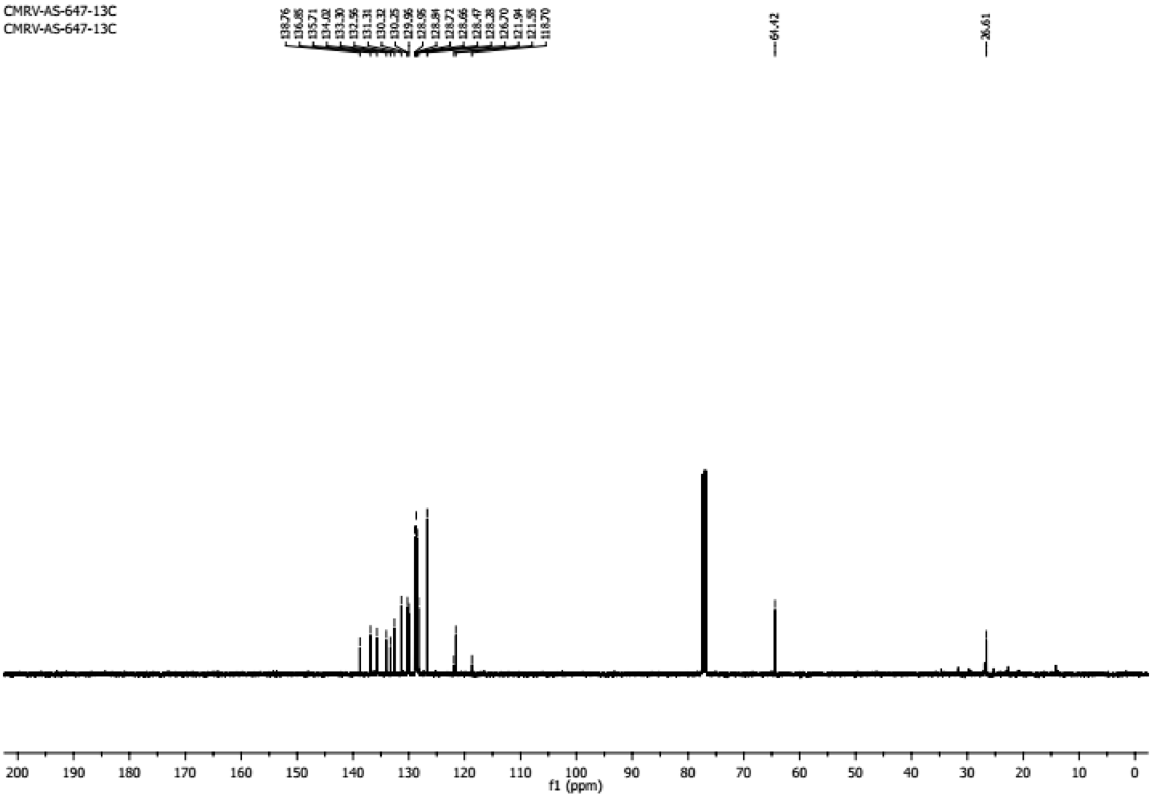

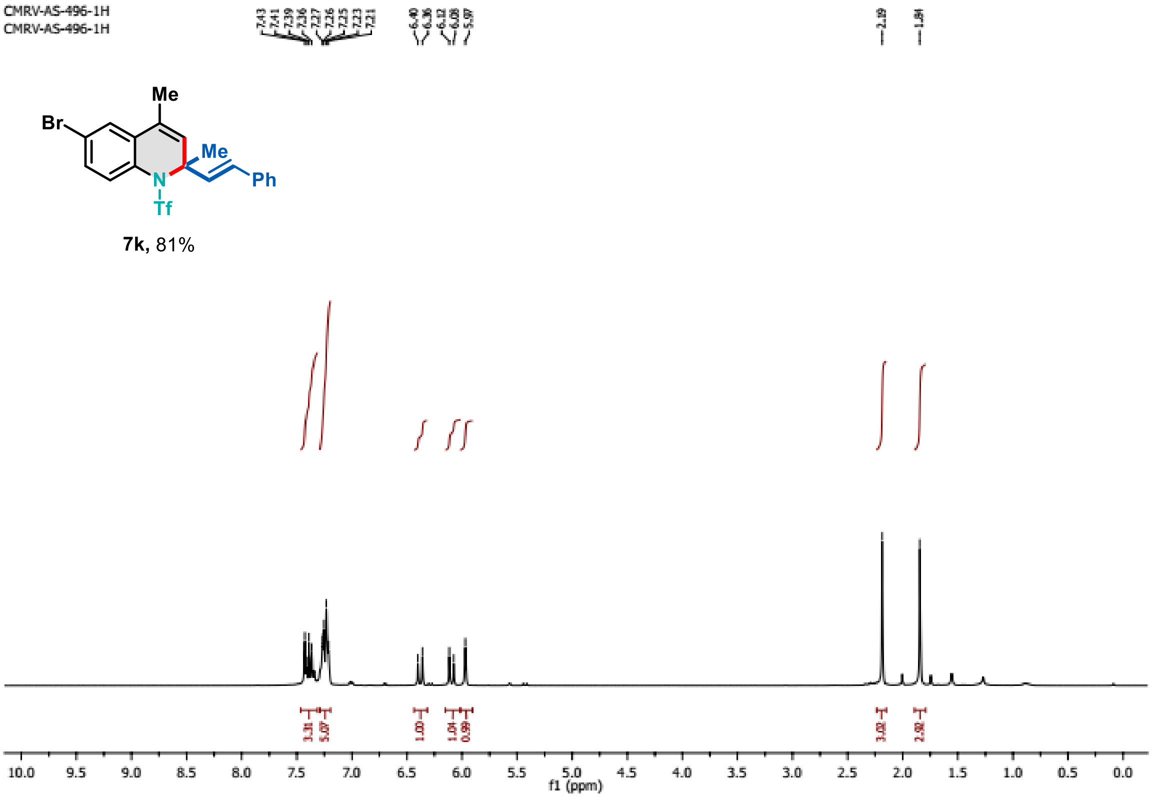
CMRV-AS-496-1H
CMRV-AS-496-1H
7.43
7.41
7.39
7.36
7.27
7.26
7.26
7.25
7.23
7.21
6.40
6.36
6.12
6.08
5.97
2.19
1.84
Me
Br
Me
N
Ph
Tf
3.31
5.07
1.00
1.04
0.99
3.02
2.92
10.0
9.5
9.0
8.5
8.0
7.5
7.0
6.5
6.0
5.5
5.0
4.5
4.0
3.5
3.0
2.5
2.0
1.5
1.0
0.5
0.0
f1 (ppm)

7k, 81%

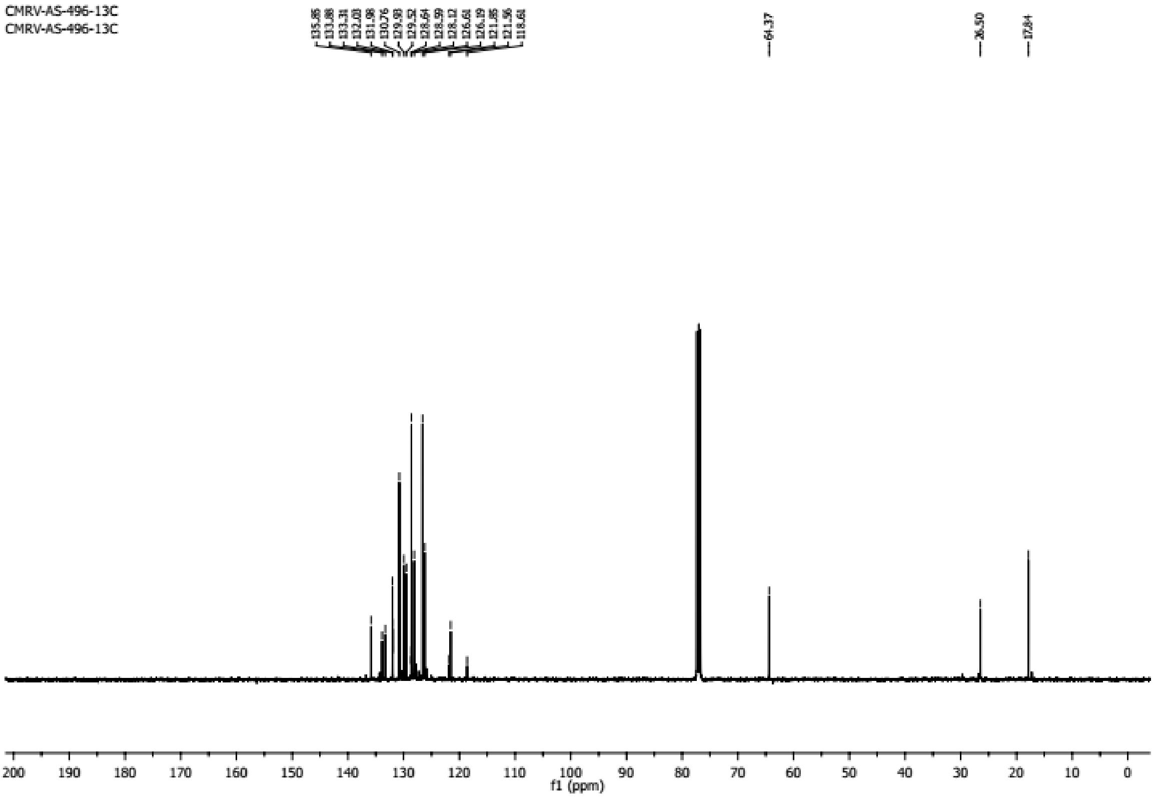
CMRV-AS-496-13C
CMRV-AS-496-13C
135.85
133.88
133.31
132.03
131.98
130.76
129.93
129.52
128.64
128.59
128.12
126.61
126.19
121.85
121.56
118.61
64.37
26.50
17.84
200
190
180
170
160
150
140
130
120
110
100
90
80
70
60
50
40
30
20
10
0
f1 (ppm)

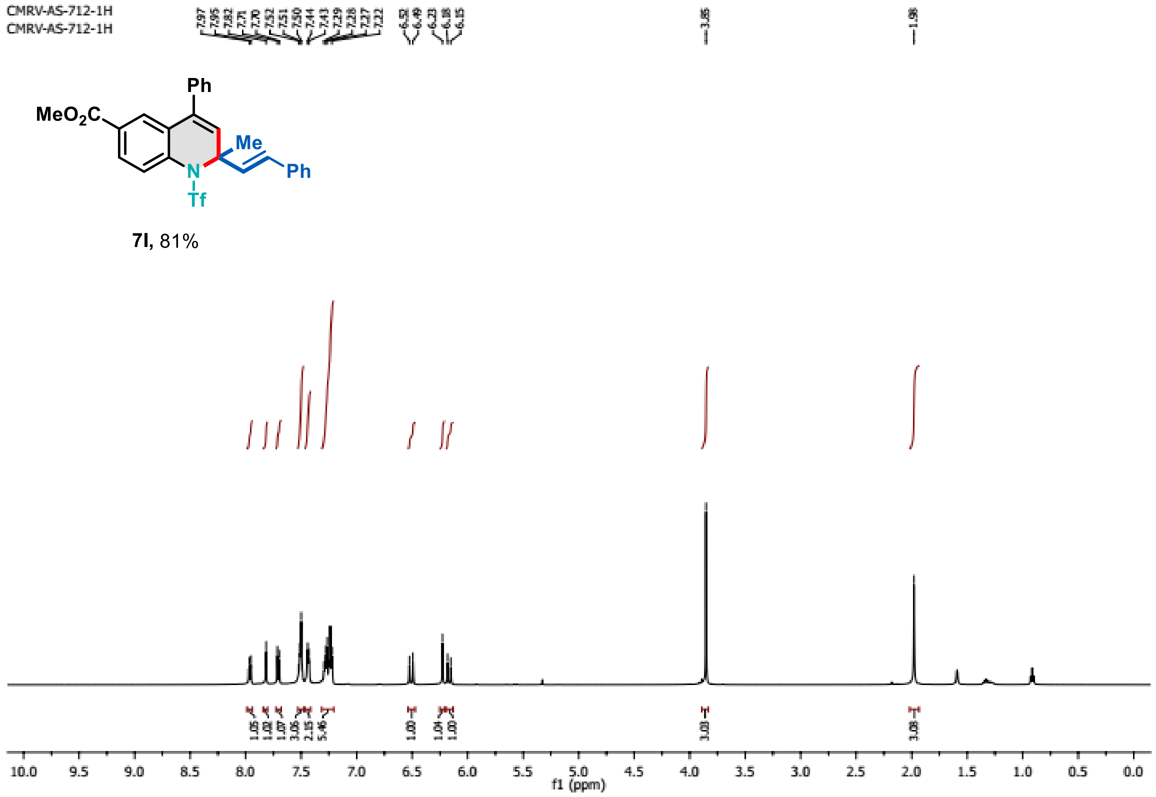
CMRV-AS-712-1H
CMRV-AS-712-1H
MeO2C
Ph
Me
Ph
N
Tf
7l, 81%
f1 (ppm)

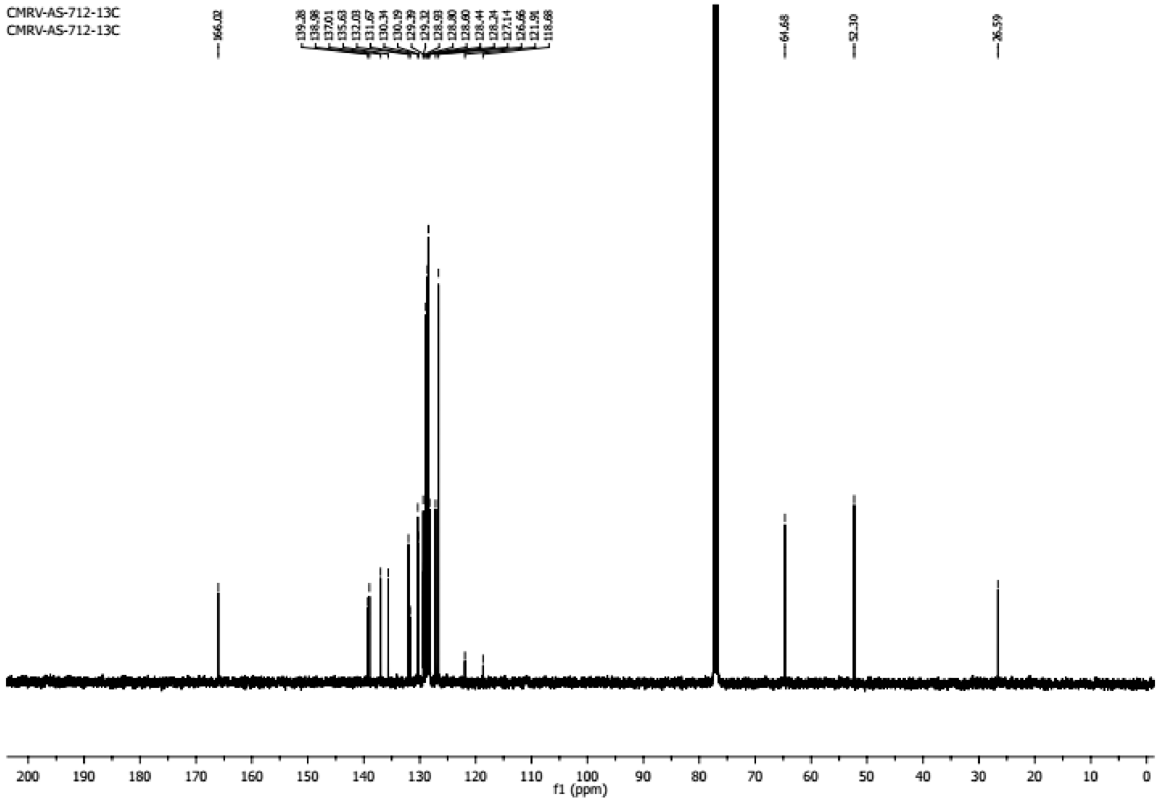
CMRV-AS-712-13C
CMRV-AS-712-13C
166.02
64.68
52.30
26.59
f1 (ppm)

CMRV-AS-722-1H
CMRV-AS-722-1H

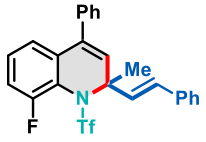

7m, 89%

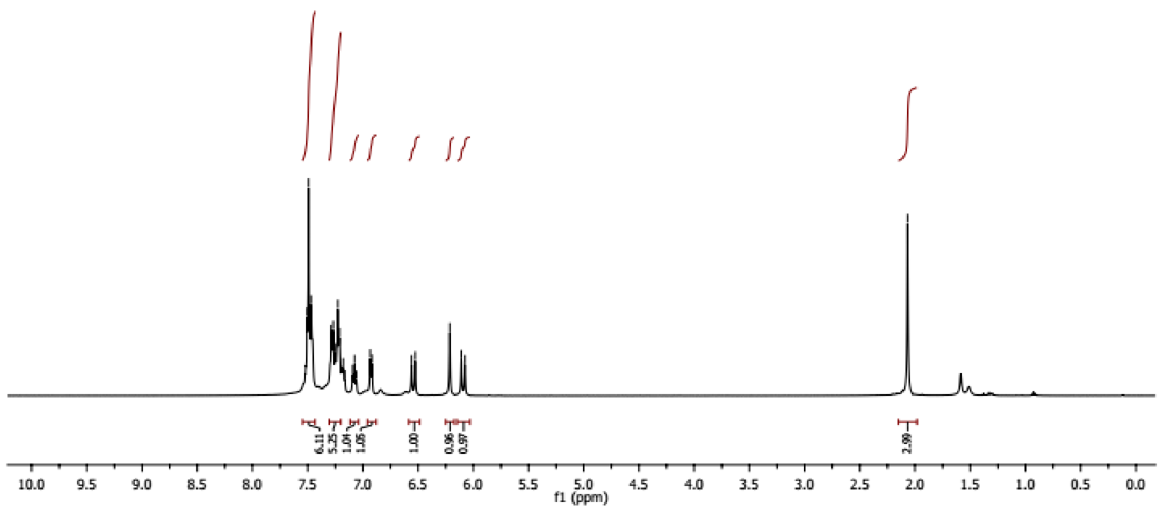

CMRV-AS-722-13C
CMRV-AS-722-13C

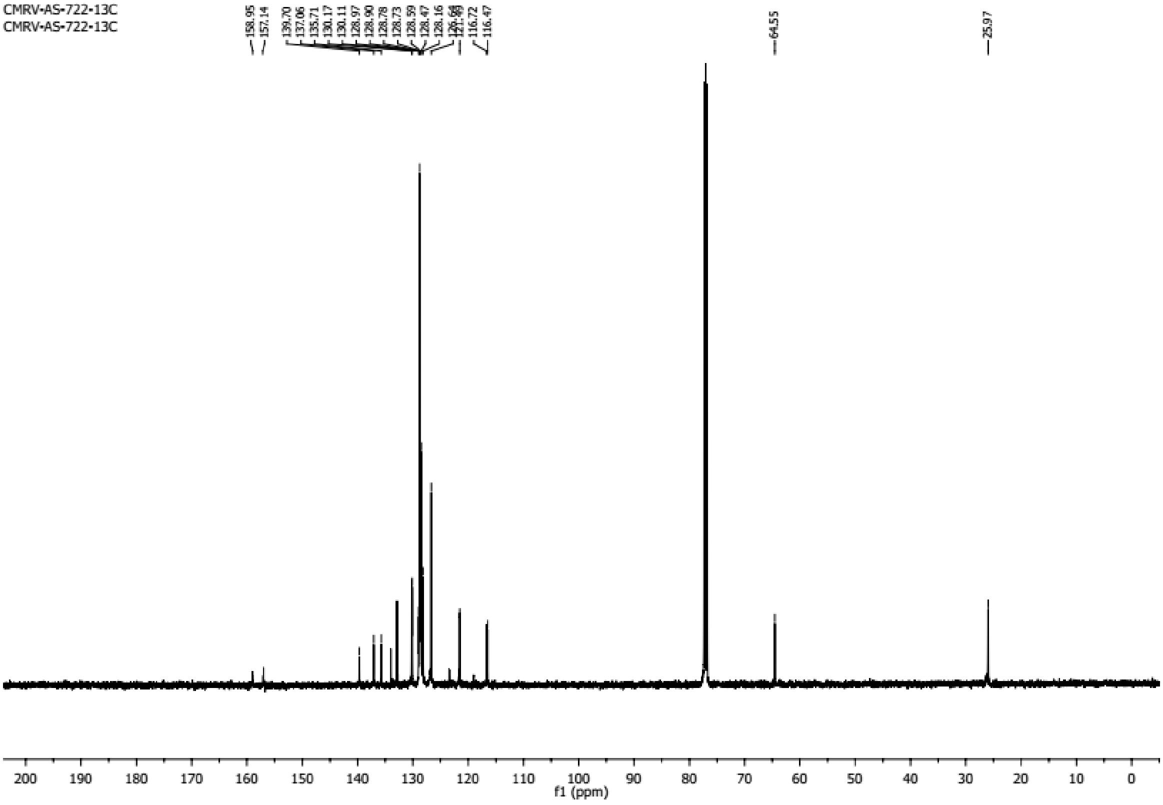

CMRV-AS-690-1H
CMRV-AS-690-1H

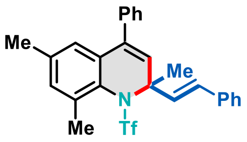

7n, 85%

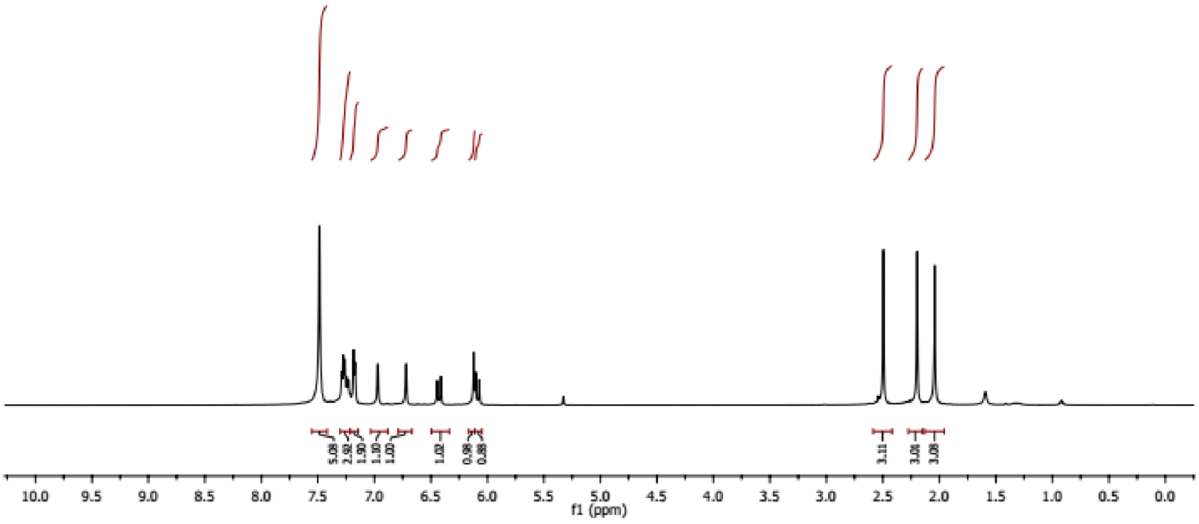

CMRV-AS-690-13C
CMRV-AS-690-13C

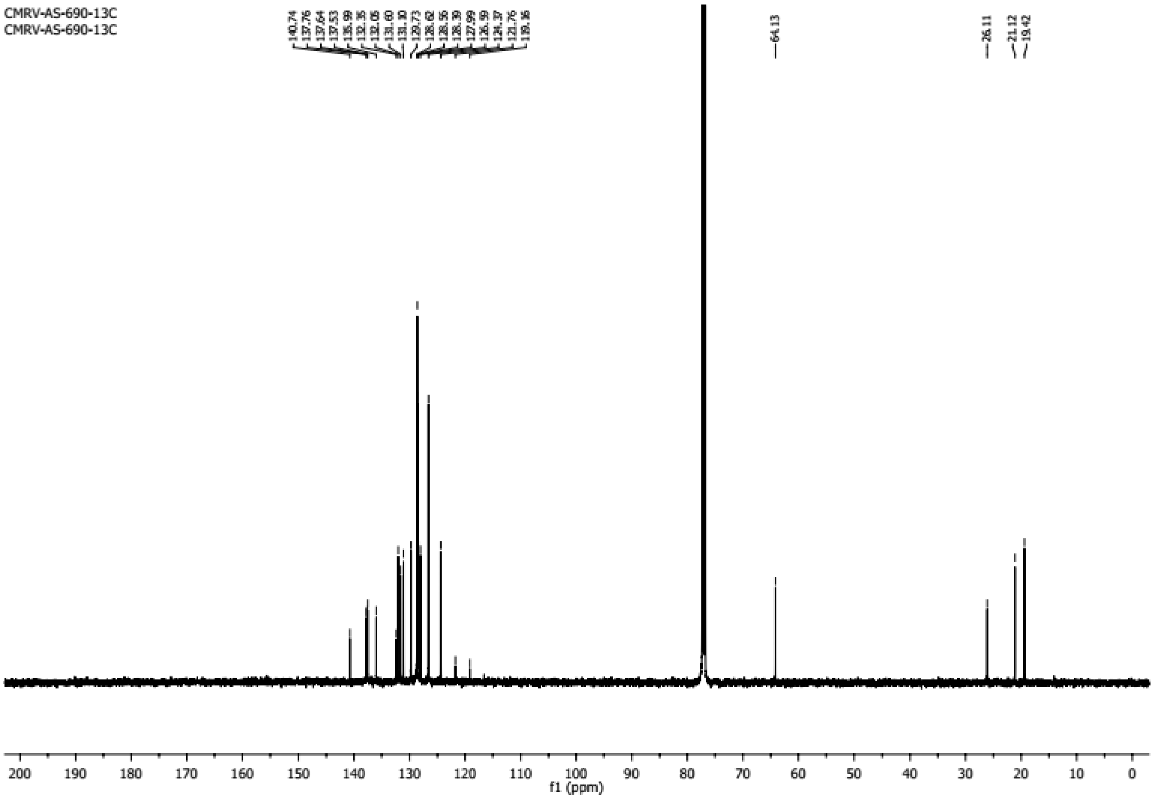

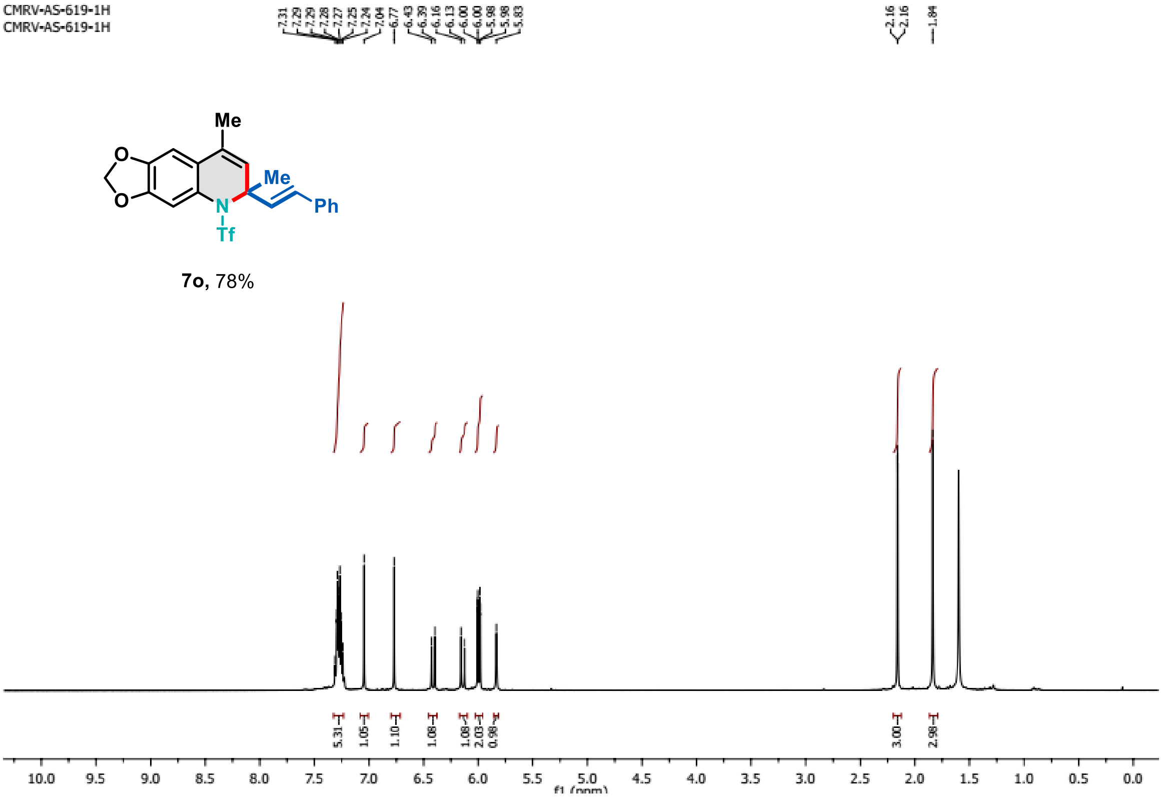
CMRV-AS-619-1H
CMRV-AS-619-1H
7.31 7.29 7.29 7.28 7.27 7.25 7.24 7.04 6.77 6.43 6.39 6.16 6.13 6.00 6.00 5.98 5.98 5.83
2.16 2.16 1.84
Me
O
O
N
Tf
Me
Ph
7o, 78%
5.31 1.05 1.10 1.08 1.08 2.03 0.98 3.00 2.98
10.0 9.5 9.0 8.5 8.0 7.5 7.0 6.5 6.0 5.5 5.0 4.5 4.0 3.5 3.0 2.5 2.0 1.5 1.0 0.5 0.0
f1 (ppm)

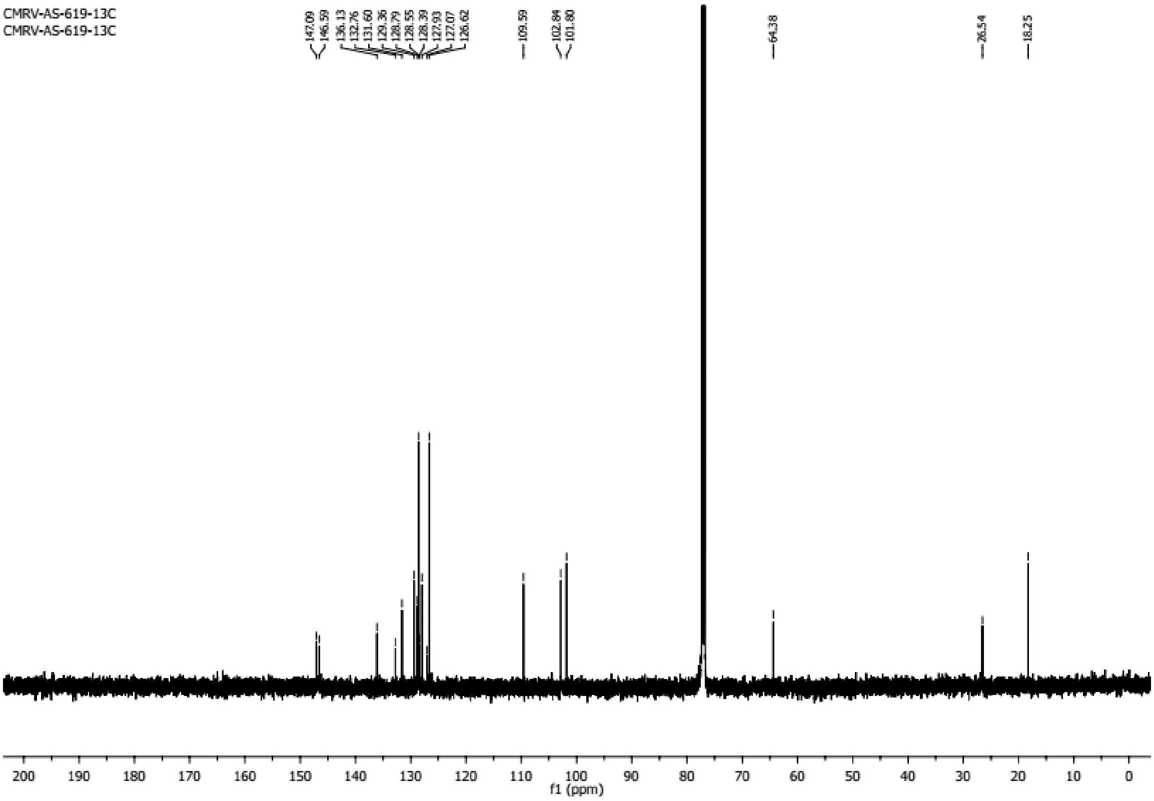
CMRV-AS-619-13C
CMRV-AS-619-13C
147.09 146.59 136.13 132.76 131.60 129.36 128.79 128.55 128.39 127.93 127.07 126.62
109.59 102.84 101.80
64.38
26.54
18.25
200 190 180 170 160 150 140 130 120 110 100 90 80 70 60 50 40 30 20 10 0
f1 (ppm)

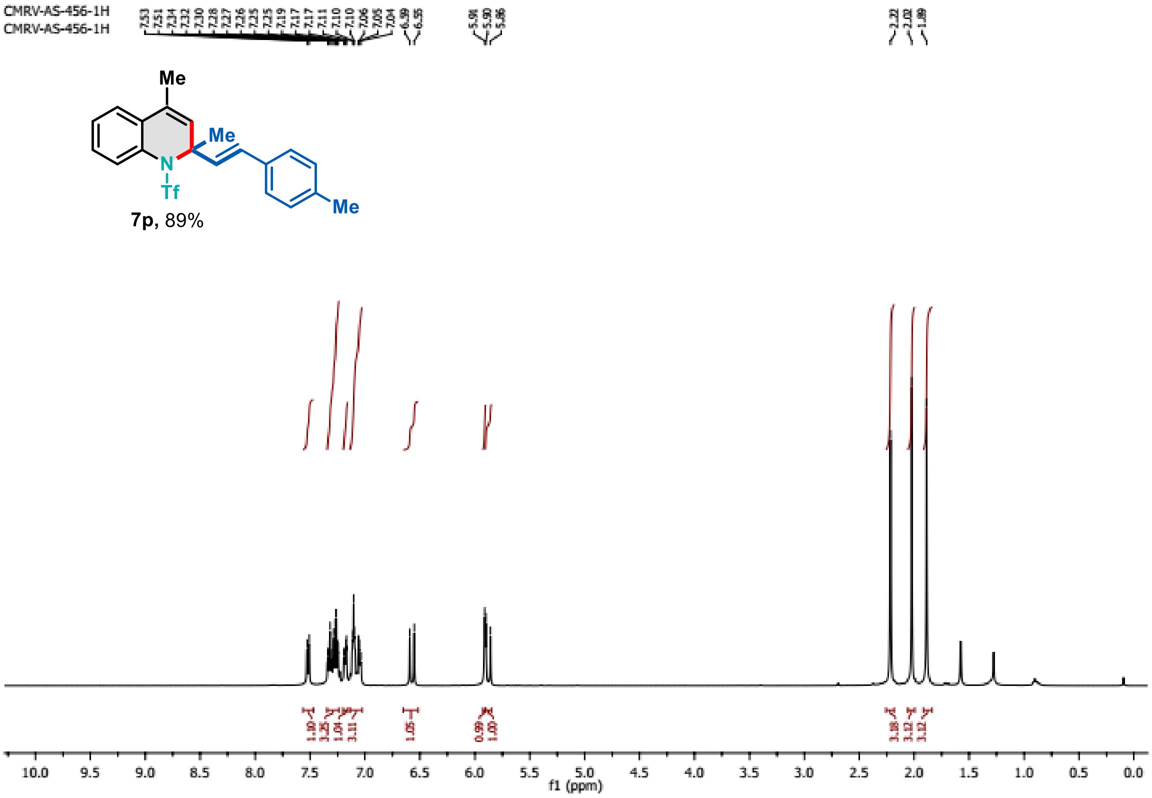
CMRV-AS-456-1H
CMRV-AS-456-1H
7.53 7.51 7.34 7.32 7.30 7.28 7.27 7.26 7.25 7.25 7.19 7.17 7.17 7.11 7.10 7.10 7.06 7.05 7.04 6.59 6.55
5.91 5.90 5.86
2.22 2.02 1.89
Me
Me
N
Tf
Me
7p, 89%
1.00 3.25 1.04 3.11 1.05 0.99 1.00 3.18 3.12 3.12
10.0 9.5 9.0 8.5 8.0 7.5 7.0 6.5 6.0 5.5 5.0 4.5 4.0 3.5 3.0 2.5 2.0 1.5 1.0 0.5 0.0
f1 (ppm)

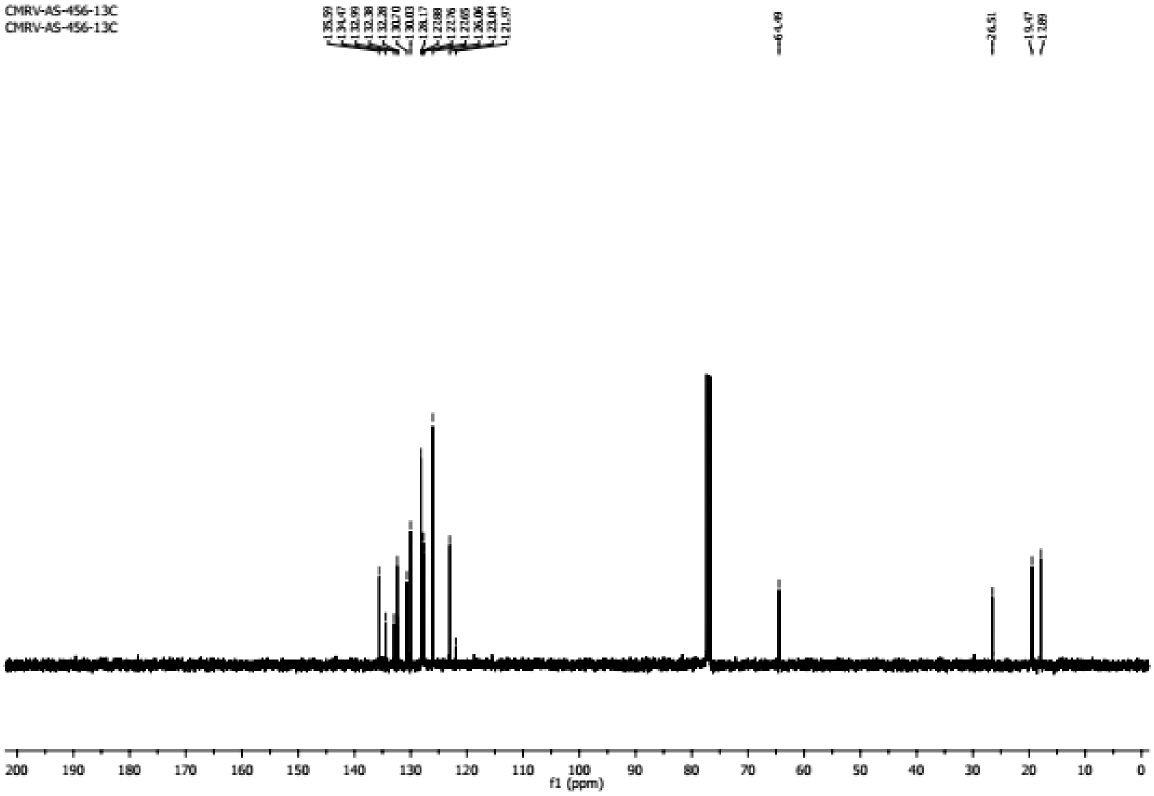
CMRV-AS-456-13C
CMRV-AS-456-13C
135.59 134.47 132.99 132.38 132.28 130.70 130.03 128.17 127.88 127.76 127.65 126.06 123.04 121.97
64.49
26.51 19.47 17.89
200 190 180 170 160 150 140 130 120 110 100 90 80 70 60 50 40 30 20 10 0
f1 (ppm)

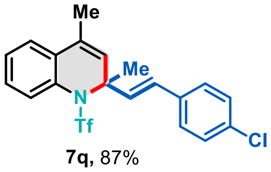
Me
Me
N
Tf
Cl

7q, 87%

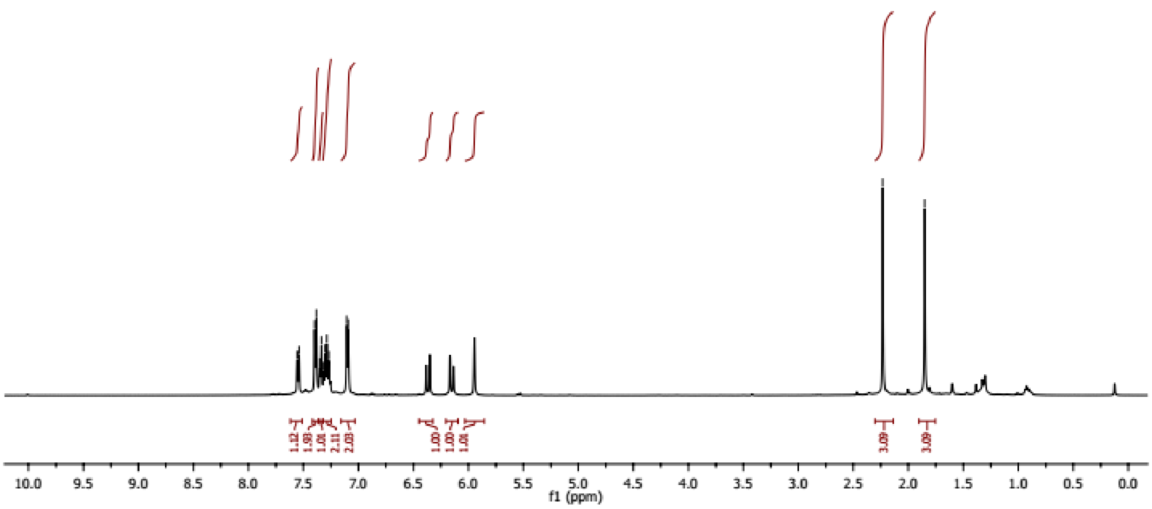
CMRV-AS-453A-1H
CMRV-AS-453A-1H
7.56
7.54
7.40
7.38
7.35
7.34
7.32
7.30
7.29
7.27
7.11
7.09
2.23
1.85
1.02
1.98
1.01
1.01
2.11
2.03
1.00
1.00
1.01
3.09
3.09
f1 (ppm)

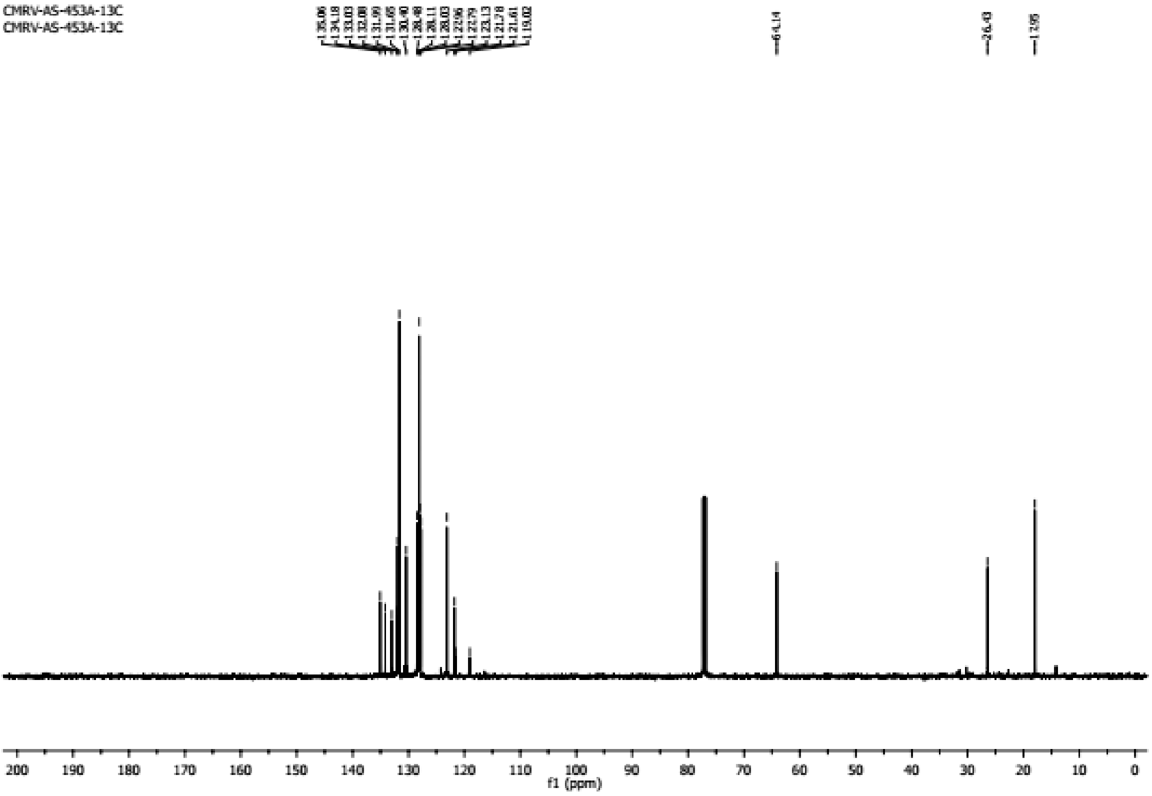
CMRV-AS-453A-13C
CMRV-AS-453A-13C
135.06
134.18
133.03
132.08
131.99
131.65
130.40
128.48
128.11
128.03
122.96
122.79
123.13
121.78
121.61
119.02
64.14
26.43
17.95
f1 (ppm)

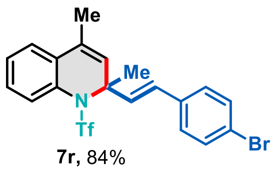

7r, 84%

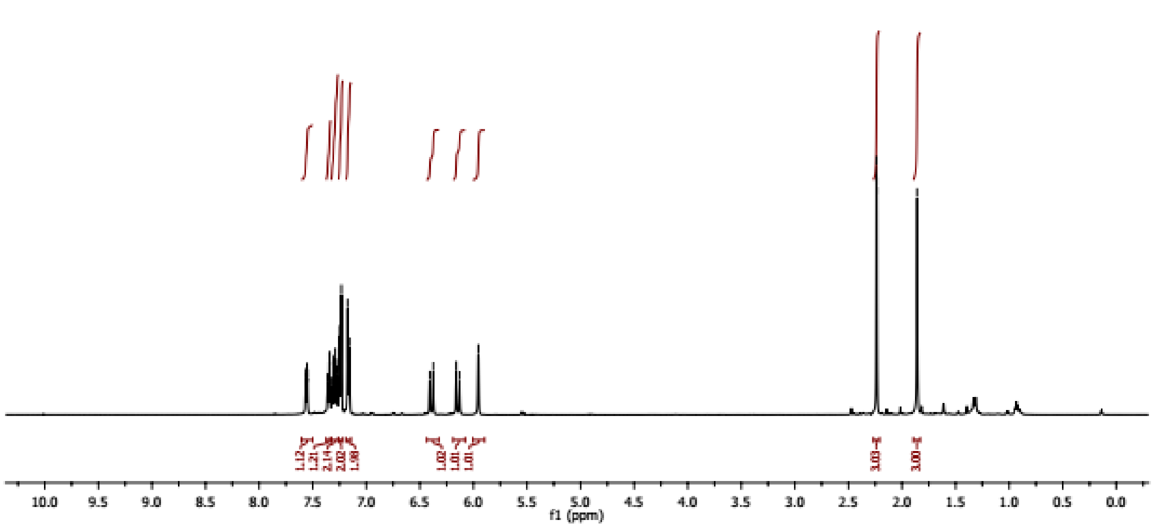

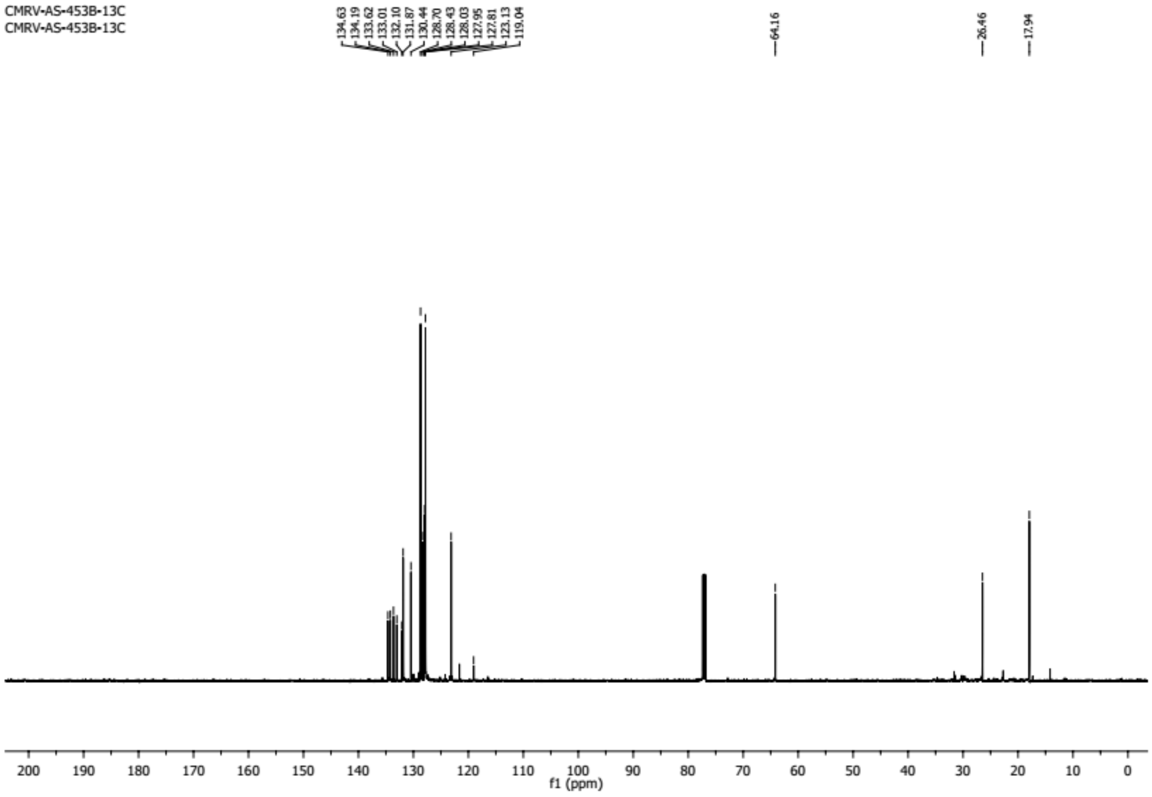

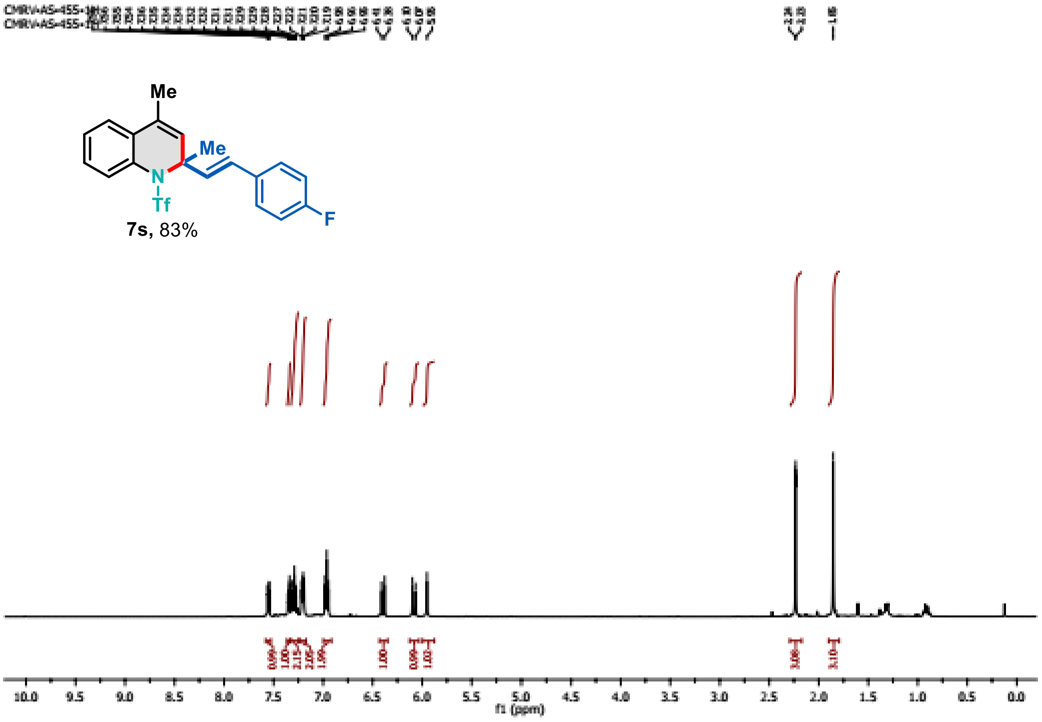
Me
Me
N
Tf
F
7s, 83%
10.0 9.5 9.0 8.5 8.0 7.5 7.0 6.5 6.0 5.5 5.0 4.5 4.0 3.5 3.0 2.5 2.0 1.5 1.0 0.5 0.0
f1 (ppm)

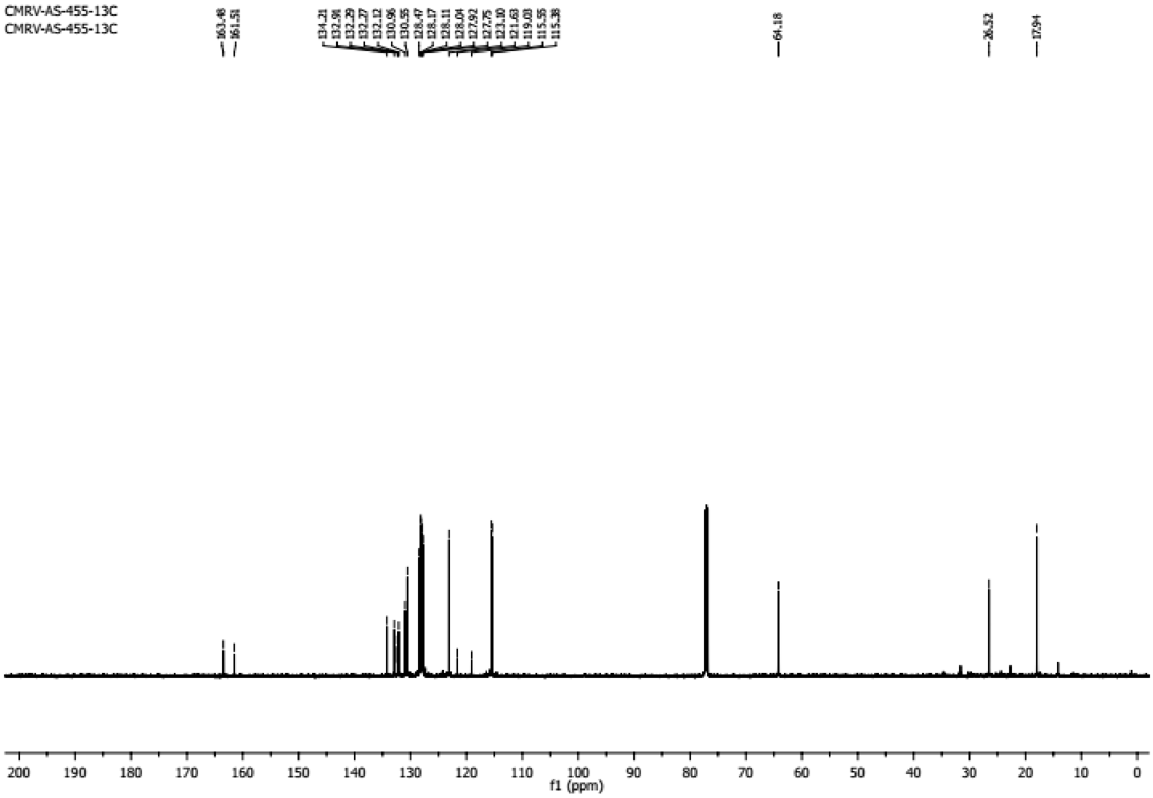
CMRV-AS-455-13C
CMRV-AS-455-13C
163.48
161.51
134.21
132.96
132.29
132.27
132.12
130.96
130.55
128.47
128.17
128.11
128.04
127.92
127.75
123.10
121.63
119.03
115.55
115.38
64.18
26.52
17.94
200 190 180 170 160 150 140 130 120 110 100 90 80 70 60 50 40 30 20 10 0
f1 (ppm)

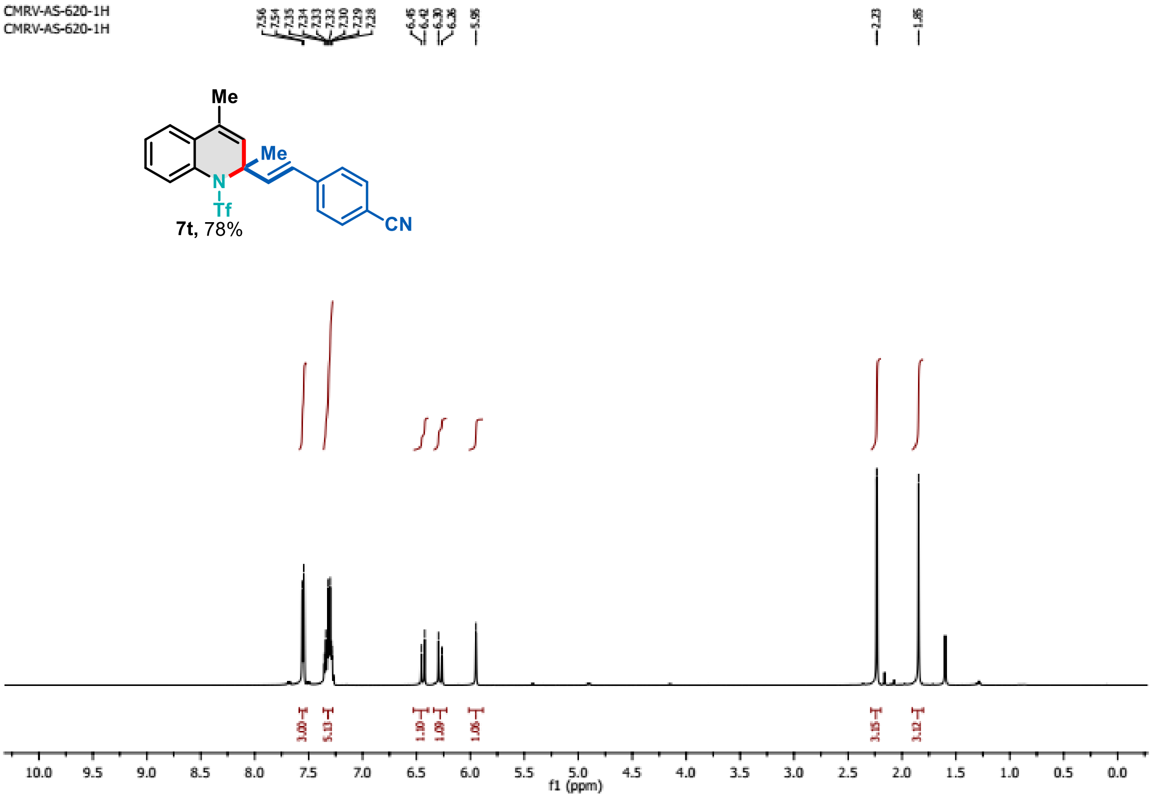
CMRV-AS-620-1H
CMRV-AS-620-1H
7.56
7.54
7.35
7.34
7.33
7.32
7.30
7.29
7.28
6.45
6.42
6.30
6.26
5.96
2.23
1.85
Me
Me
N
Tf
CN
7t, 78%
3.00
5.13
1.00
1.09
1.06
3.15
3.12
f1 (ppm)

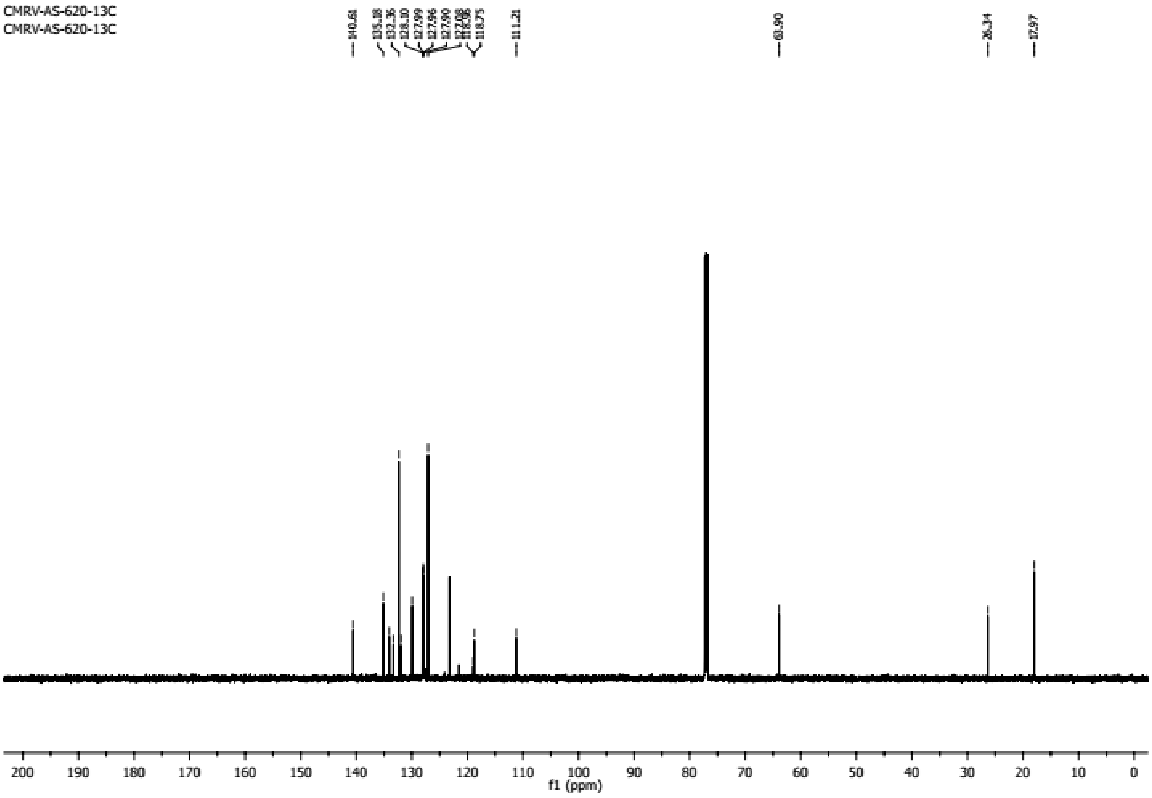
CMRV-AS-620-13C
CMRV-AS-620-13C
140.61
135.18
132.36
128.10
127.99
127.96
127.90
118.75
111.21
63.90
26.34
17.97
f1 (ppm)

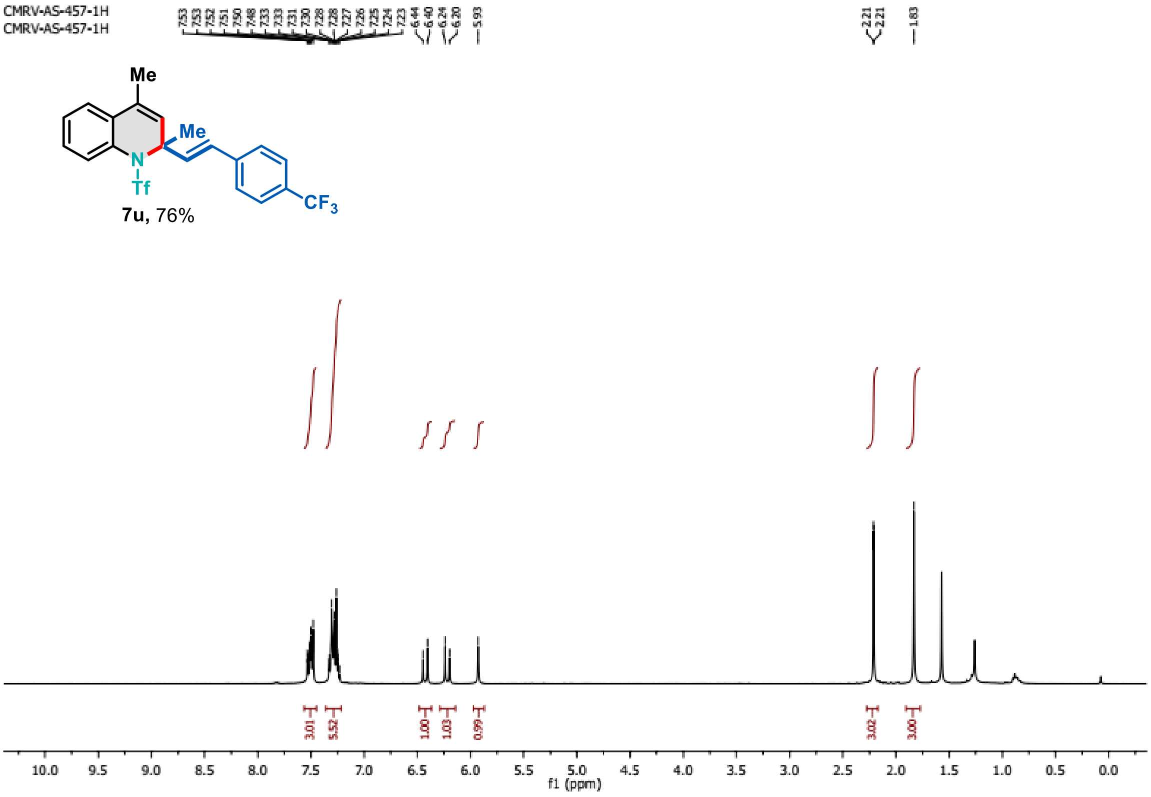
CMRV-AS-457-1H
CMRV-AS-457-1H
7.53 7.53 7.52 7.51 7.50 7.48 7.33 7.33 7.33 7.31 7.30 7.28 7.28 7.27 7.26 7.25 7.24 7.23 6.44 6.40 6.24 6.20 5.93
2.21 2.21 1.83
Me
Me
N
Tf
CF3
7u, 76%
3.01 5.52 1.00 1.03 0.99 3.02 3.00
10.0 9.5 9.0 8.5 8.0 7.5 7.0 6.5 6.0 5.5 5.0 4.5 4.0 3.5 3.0 2.5 2.0 1.5 1.0 0.5 0.0
f1 (ppm)

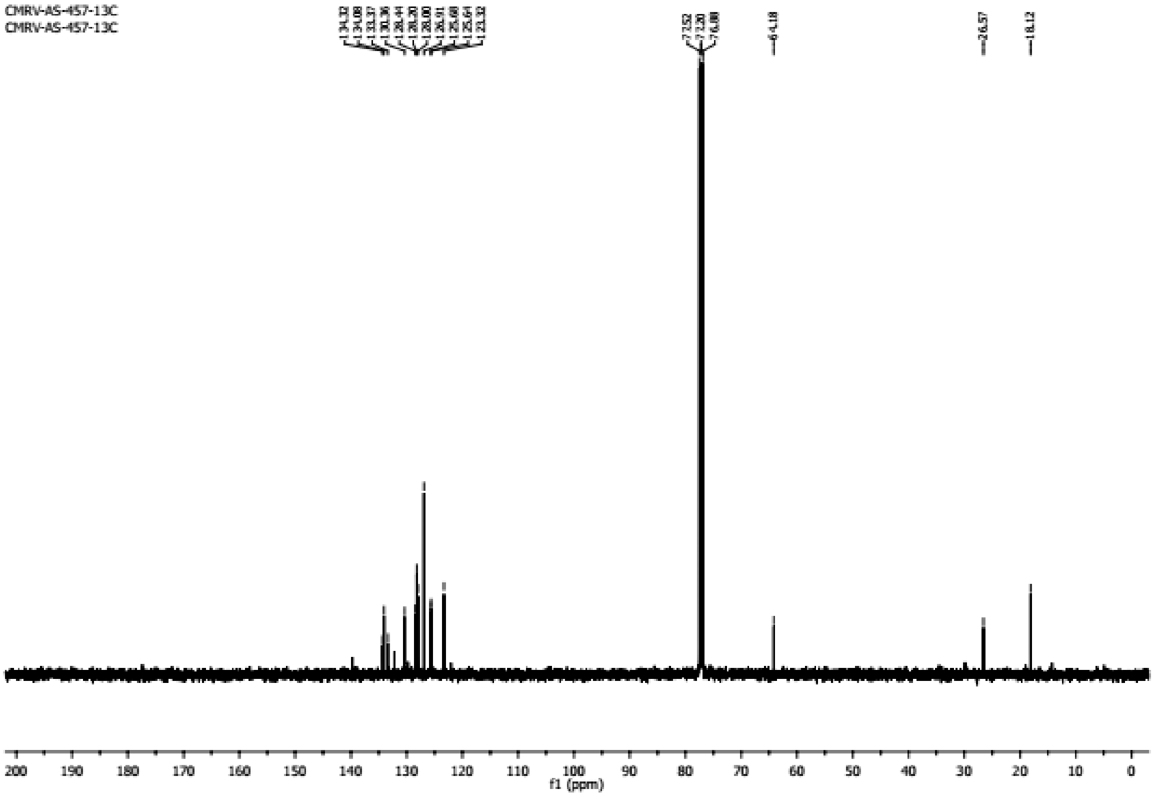
CMRV-AS-457-13C
CMRV-AS-457-13C
134.32 134.08 133.37 130.36 128.44 128.20 128.00 126.91 125.68 125.64 123.32
77.52 77.20 76.88
64.18
26.57
18.12
200 190 180 170 160 150 140 130 120 110 100 90 80 70 60 50 40 30 20 10 0
f1 (ppm)

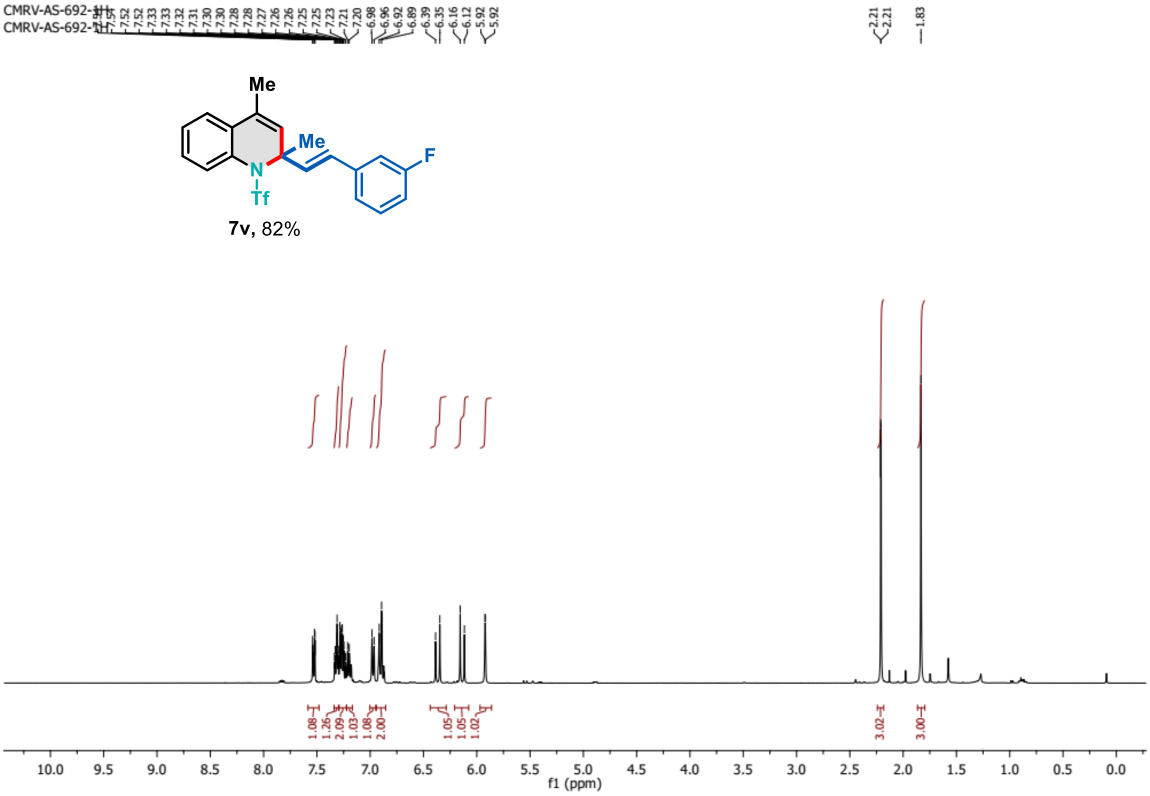
CMRV-AS-692-1H
Me
Me
N
Tf
F
7v, 82%
2.21
2.21
1.83
f1 (ppm)

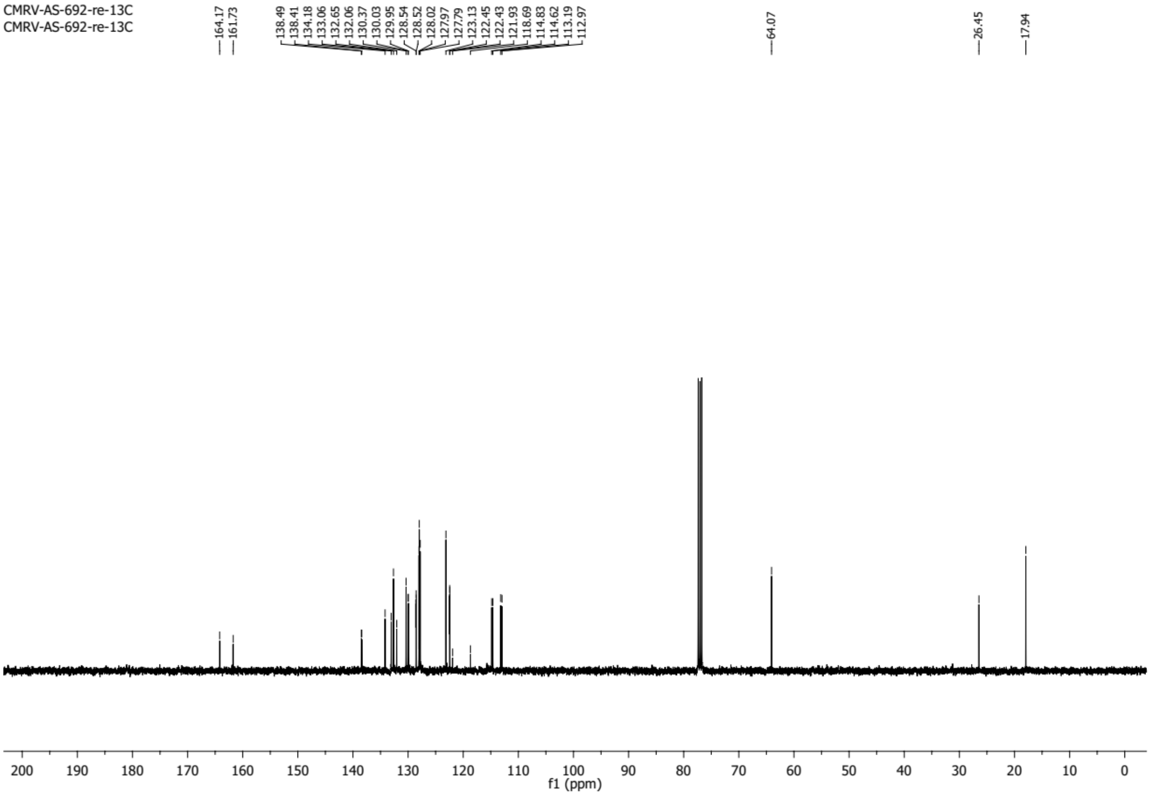
CMRV-AS-692-re-13C
CMRV-AS-692-re-13C
164.17
161.73
138.49
138.41
134.18
133.06
132.65
132.06
130.37
130.03
129.95
128.54
128.52
128.02
127.97
127.79
123.13
122.45
122.43
121.93
118.69
114.83
114.62
113.19
112.97
64.07
26.45
17.94
f1 (ppm)

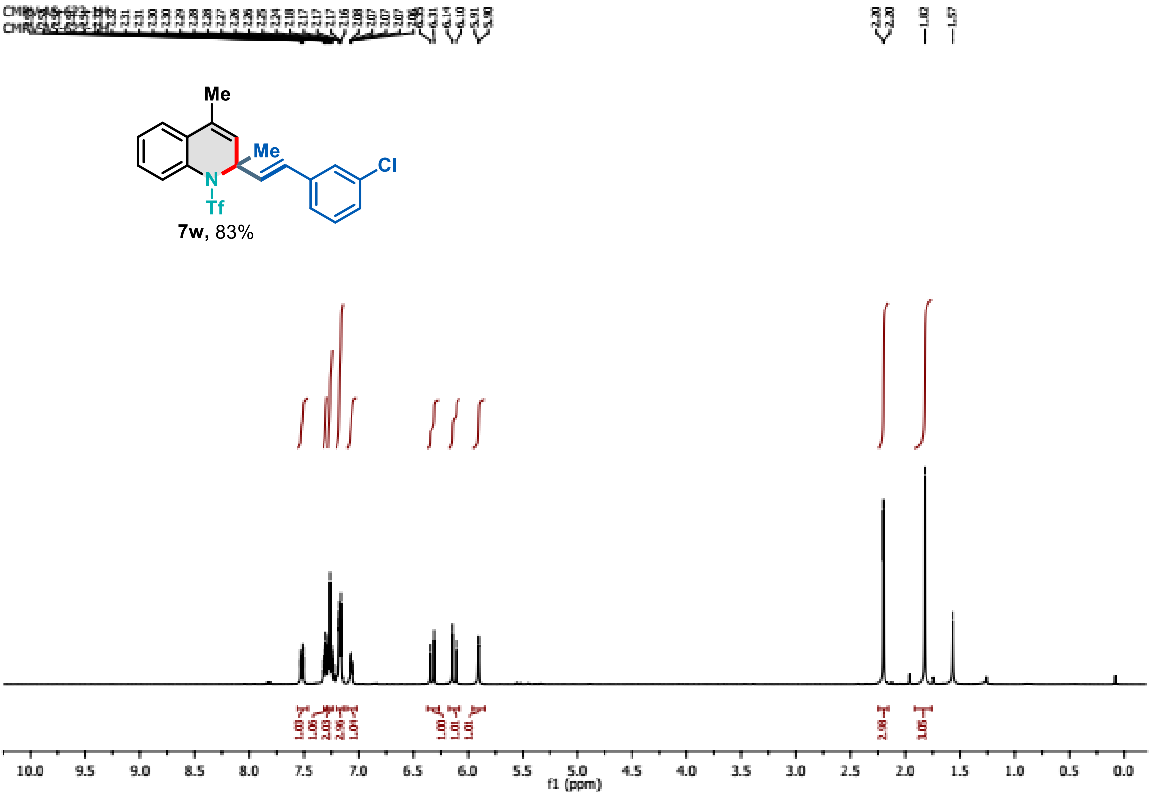
Me
Me
N
Tf
Cl
7w, 83%
2.20
2.20
1.82
1.57
f1 (ppm)

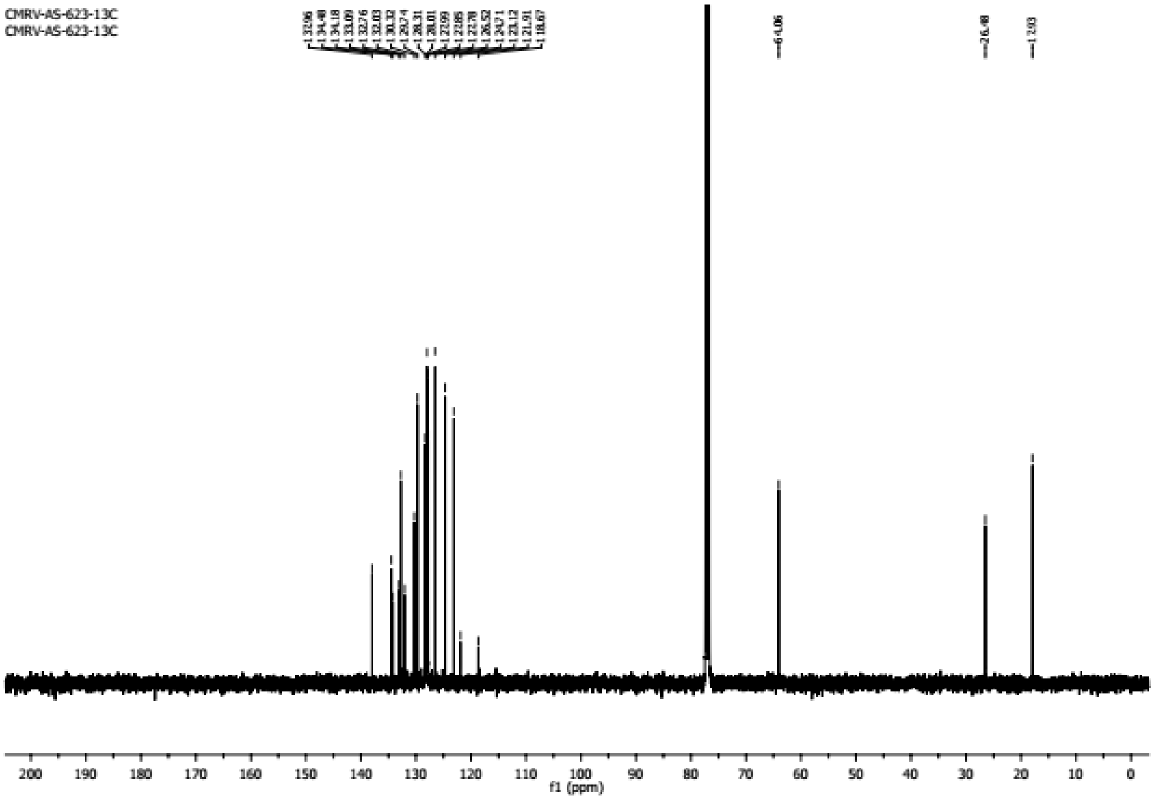
CMRV-AS-623-13C
CMRV-AS-623-13C
64.06
26.48
f1 (ppm)

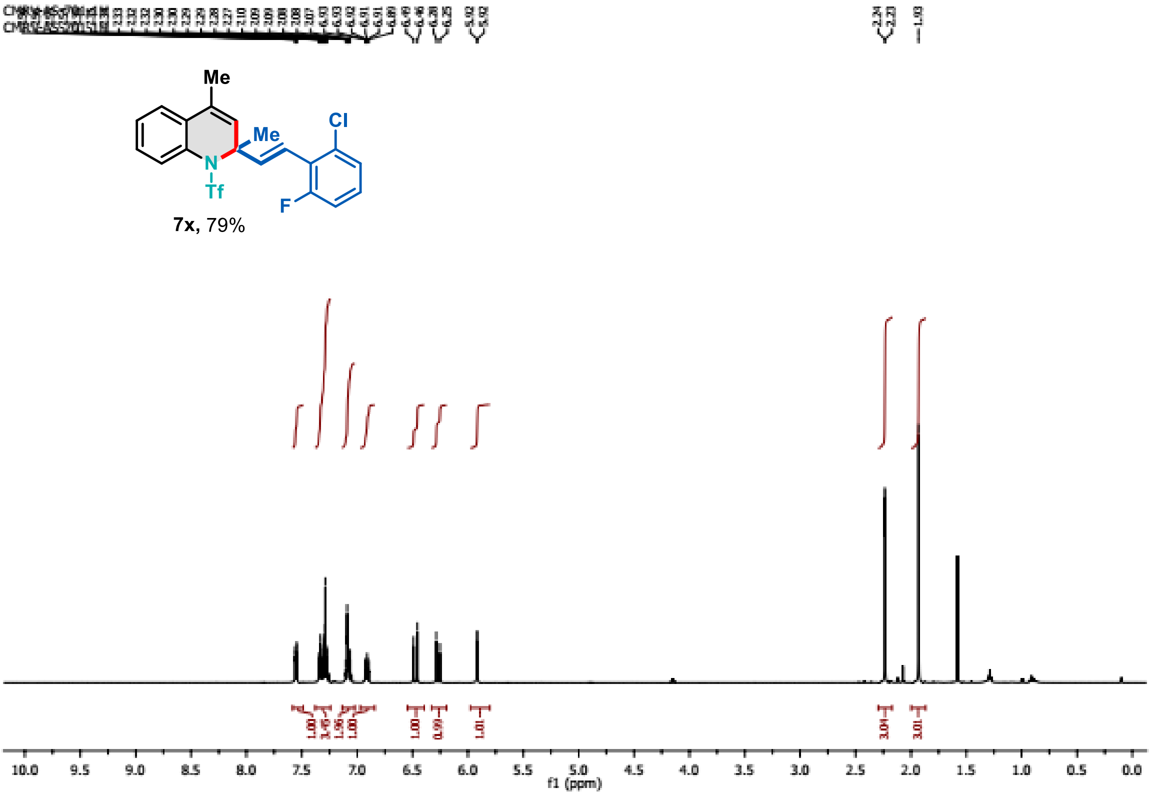
Me
Me
Cl
N
Tf
F
7x, 79%
f1 (ppm)

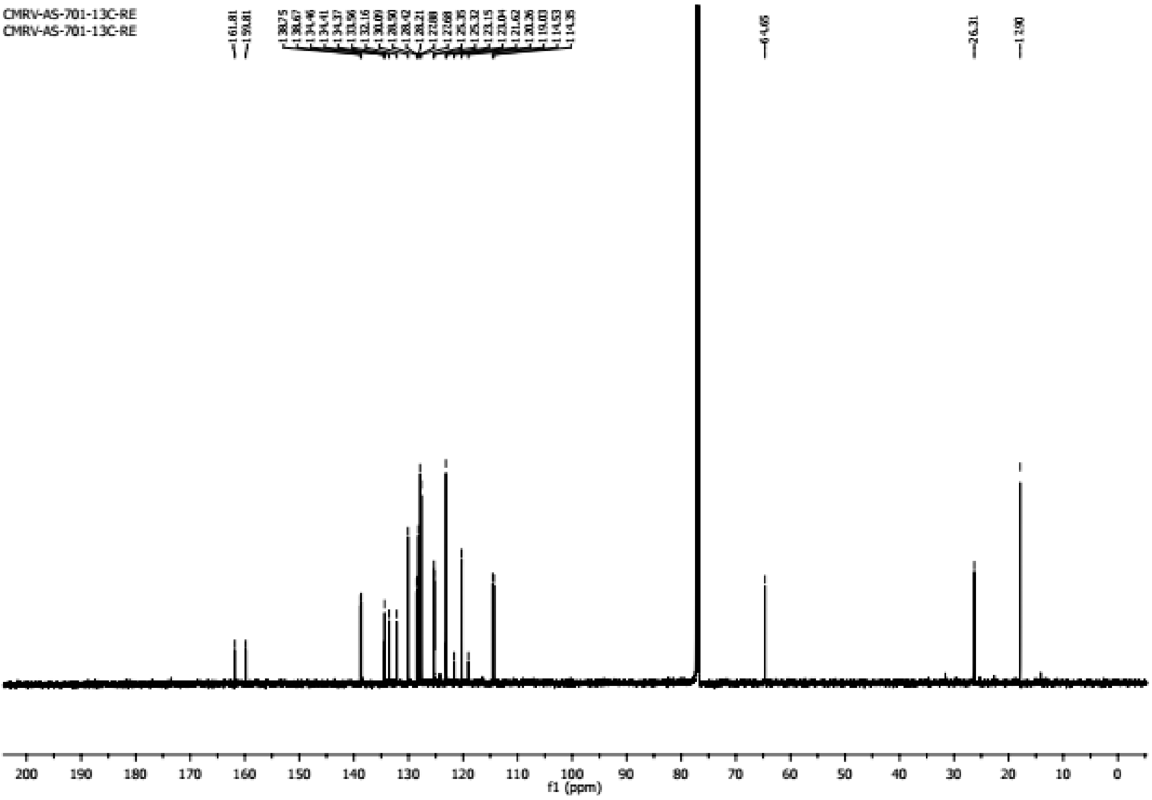
CMRV-AS-701-13C-RE
CMRV-AS-701-13C-RE
f1 (ppm)

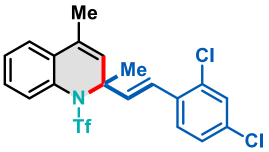
Me
Me
Cl
N
Tf
Cl

7y, 81%

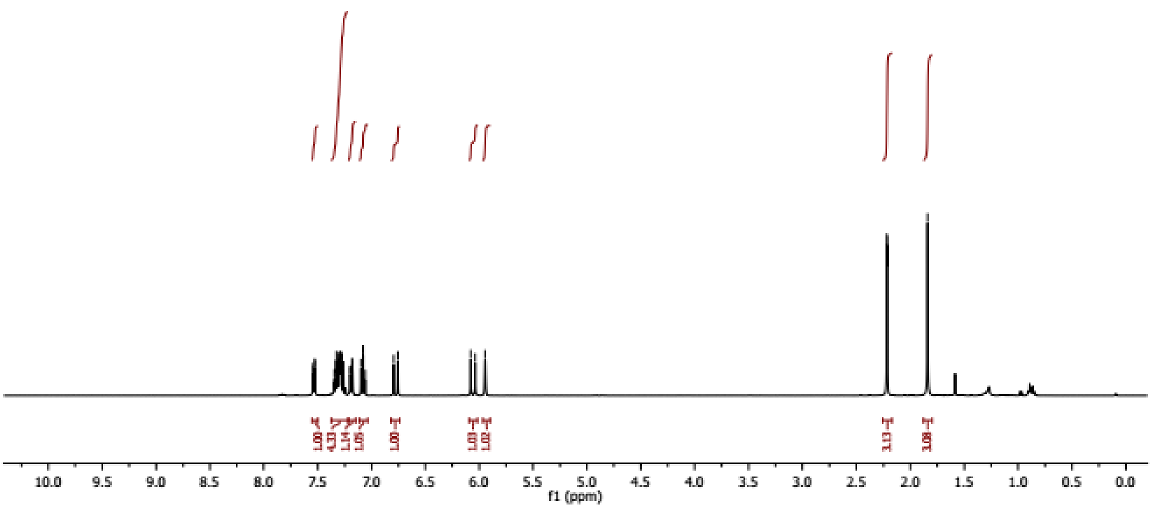
6.08
6.04
5.94
2.22
2.22
1.84
1.00
1.00
1.03
1.02
3.13
3.08
10.0
9.5
9.0
8.5
8.0
7.5
7.0
6.5
6.0
5.5
5.0
4.5
4.0
3.5
3.0
2.5
2.0
1.5
1.0
0.5
0.0
f1 (ppm)

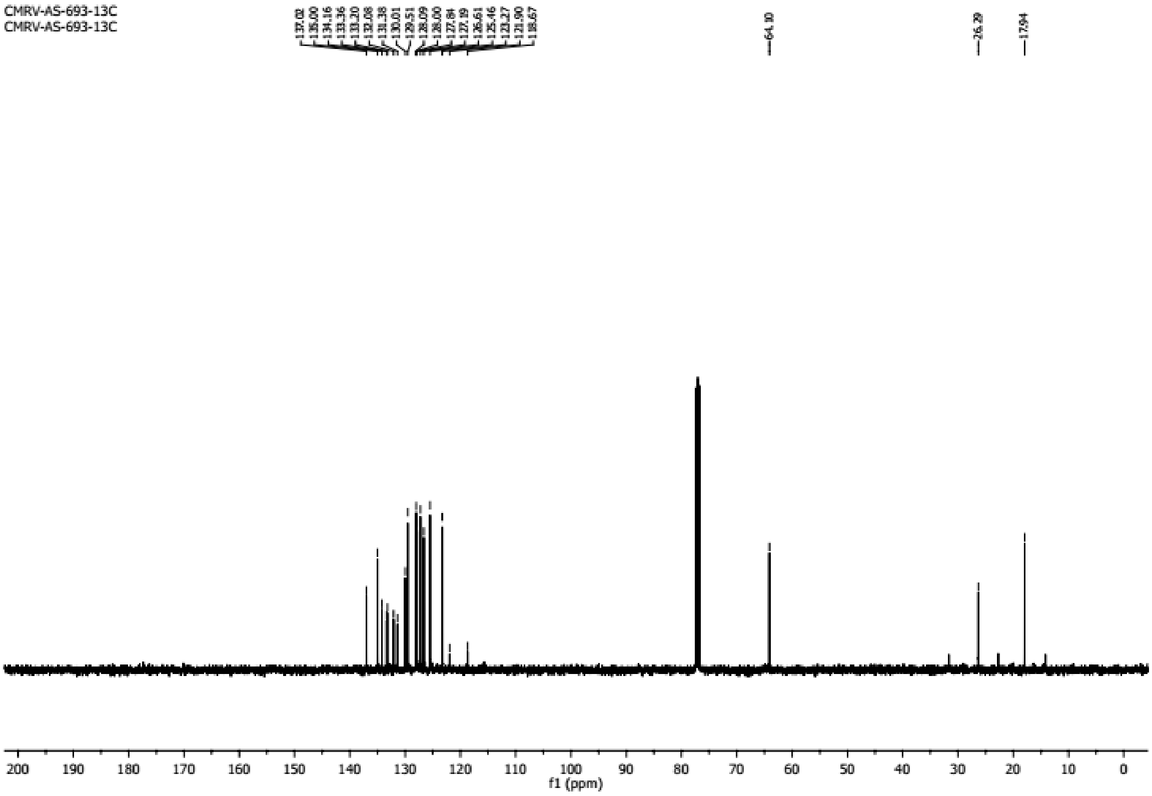
CMRV-AS-693-13C
CMRV-AS-693-13C
137.02
135.00
134.16
133.36
133.20
132.08
131.38
130.01
129.51
128.09
128.00
127.84
127.19
126.61
125.46
123.27
121.90
118.67
64.10
26.29
17.94
200
190
180
170
160
150
140
130
120
110
100
90
80
70
60
50
40
30
20
10
0
f1 (ppm)

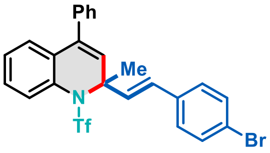

Ph
Me
N
Tf
Br

7z, 84%

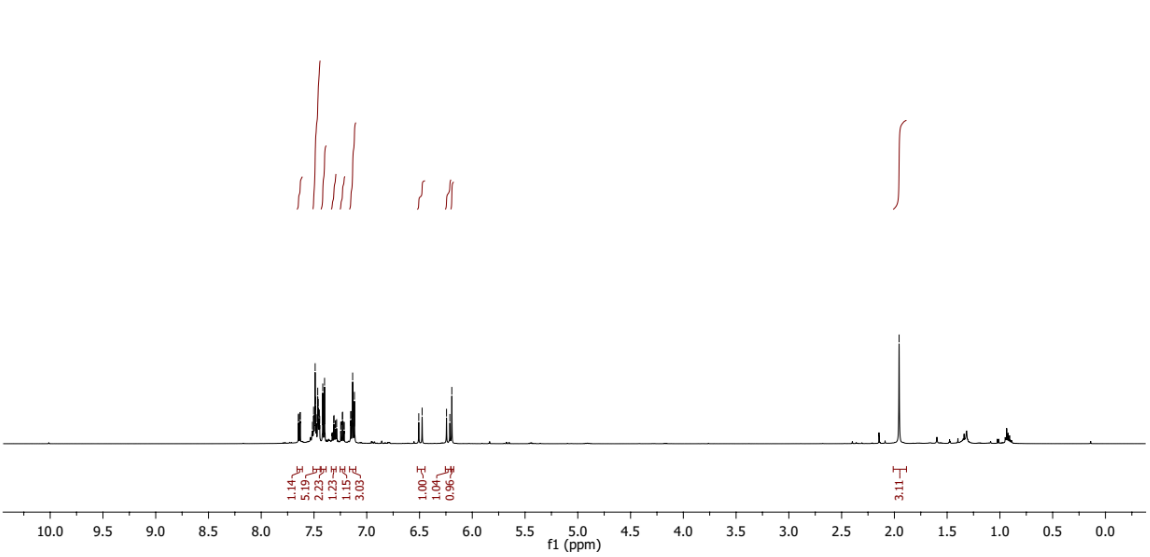

CMRV-AS-468-Br-1H
CMRV-AS-468-Br-13C
7.48
7.46
7.46
7.45
7.45
7.45
7.45
7.42
7.41
7.40
7.40
7.31
7.31
7.29
7.23
7.23
7.15
7.15
7.13
6.51
6.48
6.24
6.21
6.19
1.96
1.14
5.19
2.23
1.23
1.15
3.03
1.00
1.04
0.96
3.11
10.0 9.5 9.0 8.5 8.0 7.5 7.0 6.5 6.0 5.5 5.0 4.5 4.0 3.5 3.0 2.5 2.0 1.5 1.0 0.5 0.0
f1 (ppm)

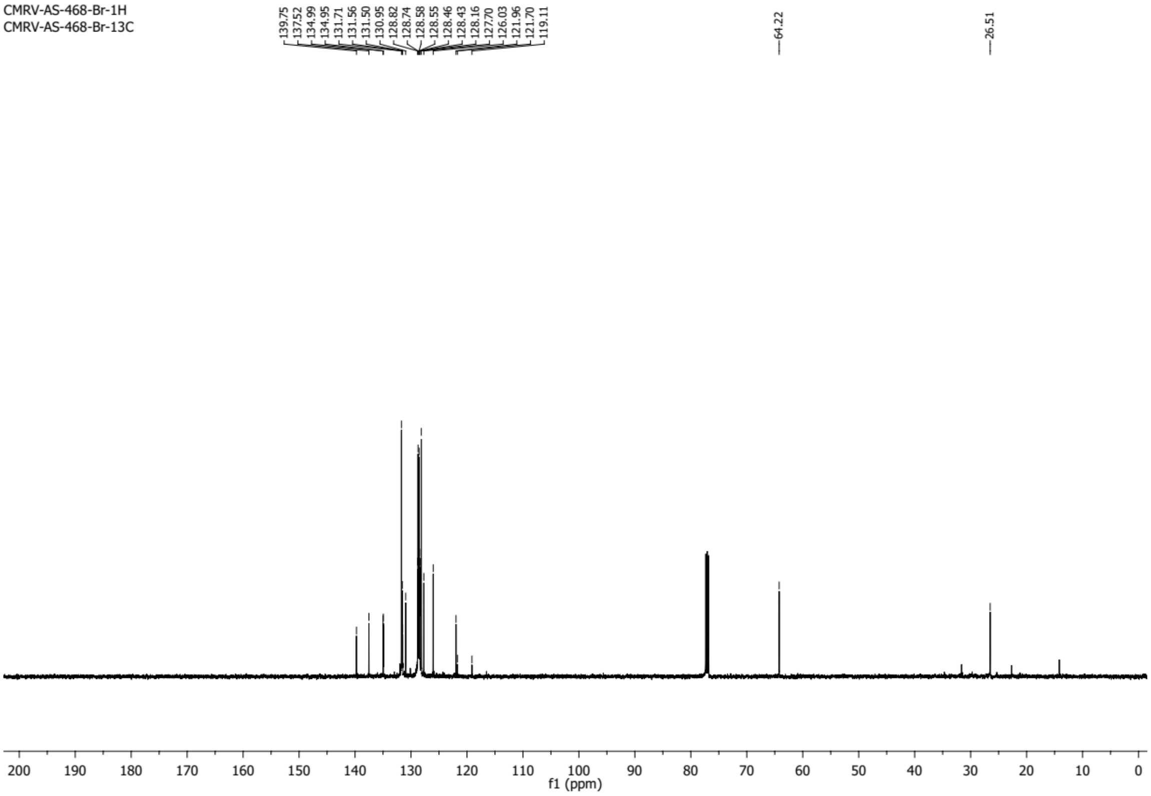

CMRV-AS-468-Br-1H
CMRV-AS-468-Br-13C
139.75
137.52
134.99
134.95
131.71
131.56
131.50
130.95
128.82
128.74
128.58
128.55
128.46
128.43
128.16
127.70
126.03
121.96
121.70
119.11
64.22
26.51
200 190 180 170 160 150 140 130 120 110 100 90 80 70 60 50 40 30 20 10 0
f1 (ppm)

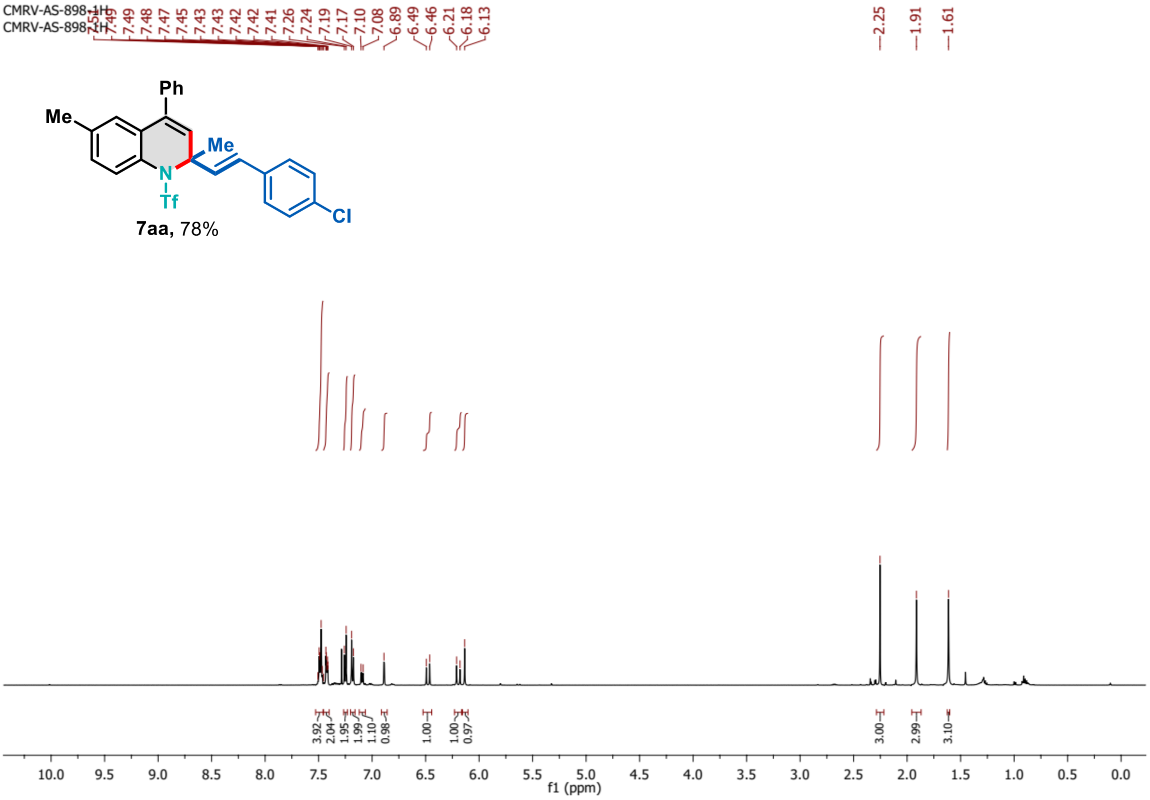
CMRV-AS-898-1H
7.49
7.49
7.48
7.47
7.45
7.43
7.43
7.42
7.42
7.41
7.26
7.24
7.19
7.17
7.10
7.08
6.89
6.49
6.46
6.21
6.18
6.13
2.25
1.91
1.61
Ph
Me
Me
N
Tf
Cl
7aa, 78%
3.92
2.04
1.95
1.99
1.10
0.98
1.00
1.00
0.97
3.00
2.99
3.10
f1 (ppm)

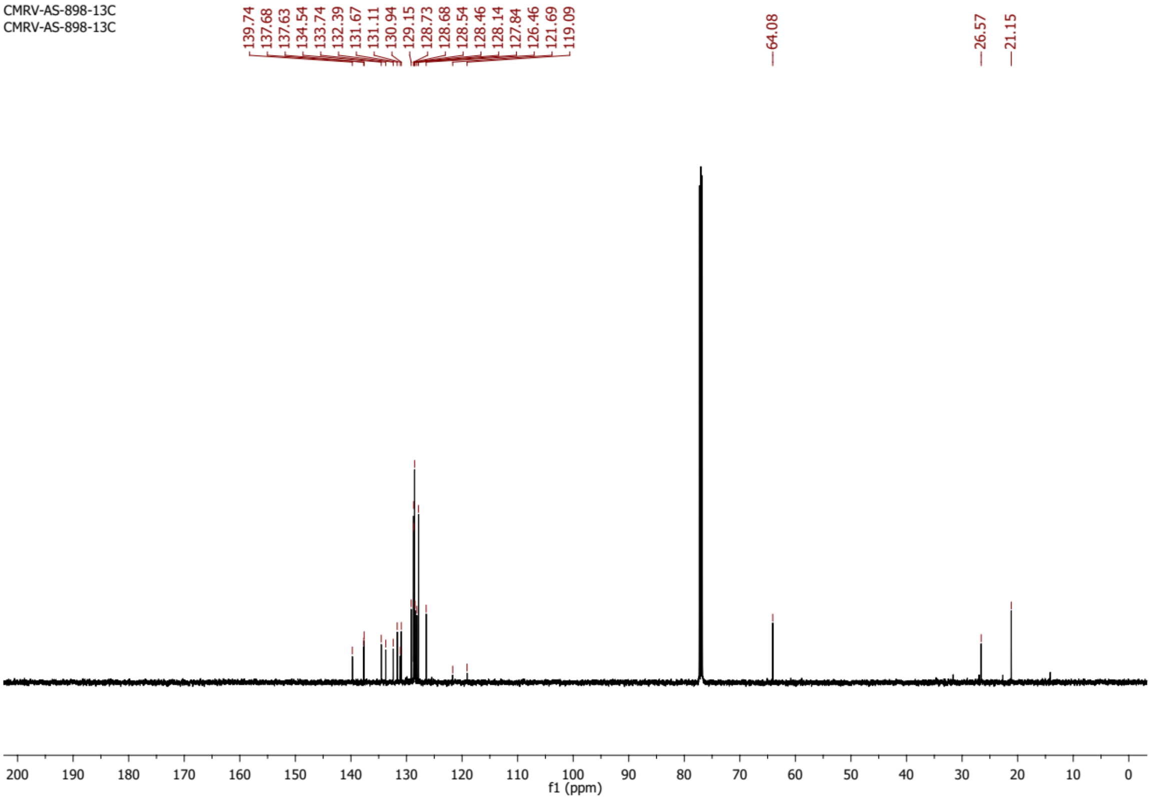
CMRV-AS-898-13C
CMRV-AS-898-13C
139.74
137.68
137.63
134.54
133.74
132.39
131.67
131.11
130.94
129.15
128.73
128.68
128.54
128.46
128.14
127.84
126.46
121.69
119.09
64.08
26.57
21.15
f1 (ppm)

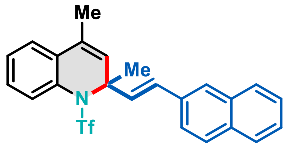

7ab, 84%

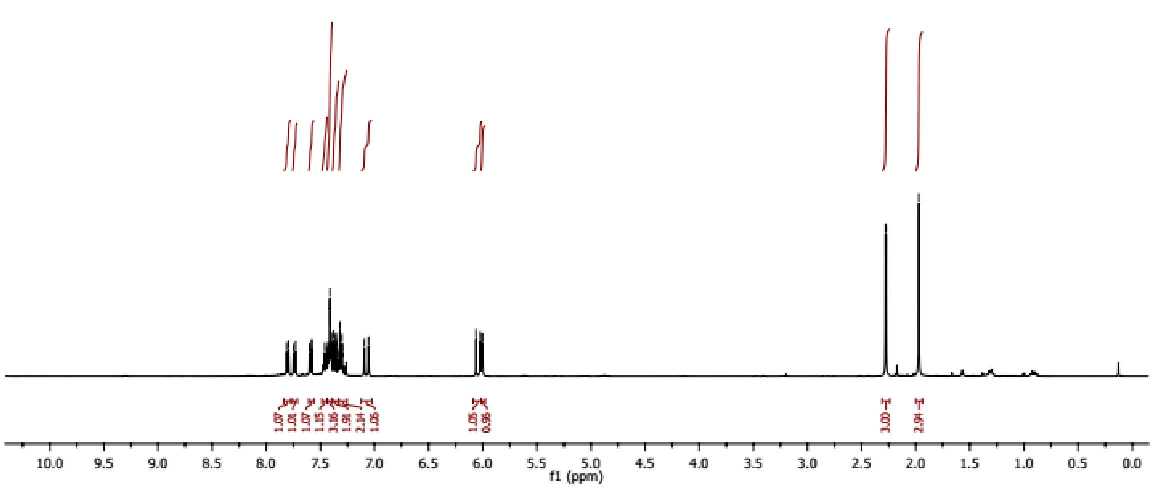

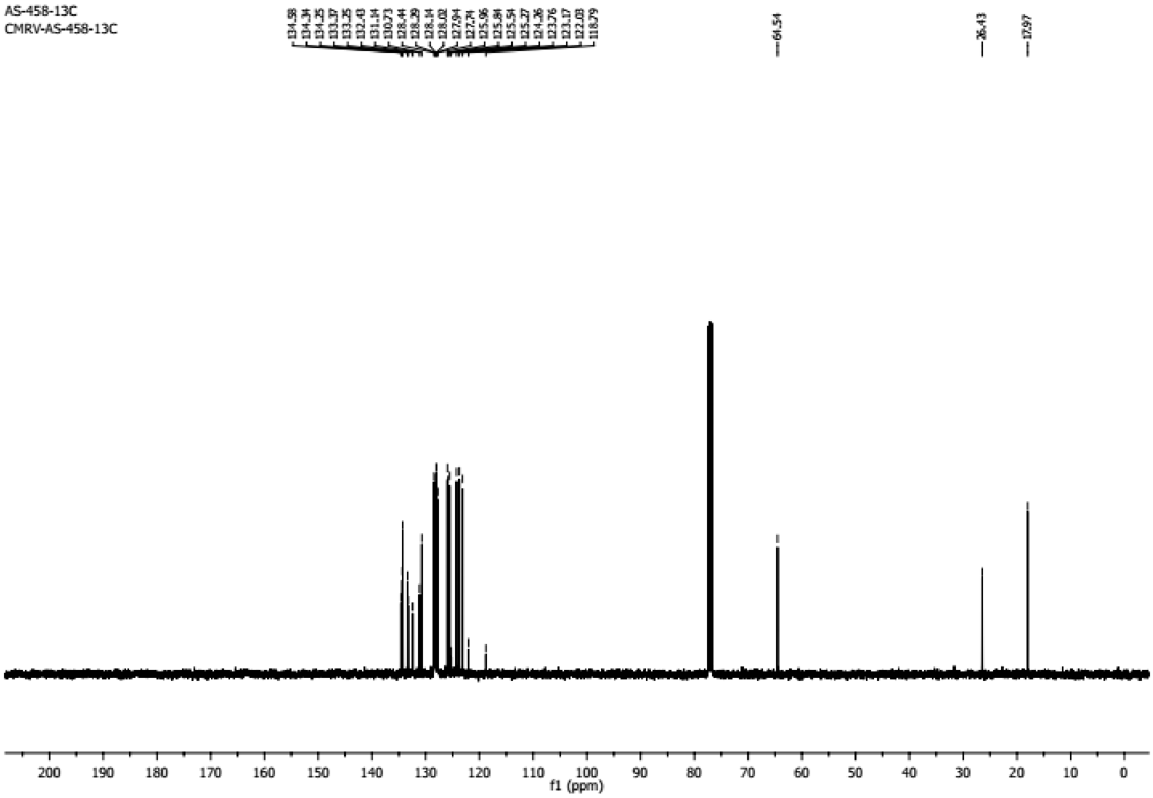

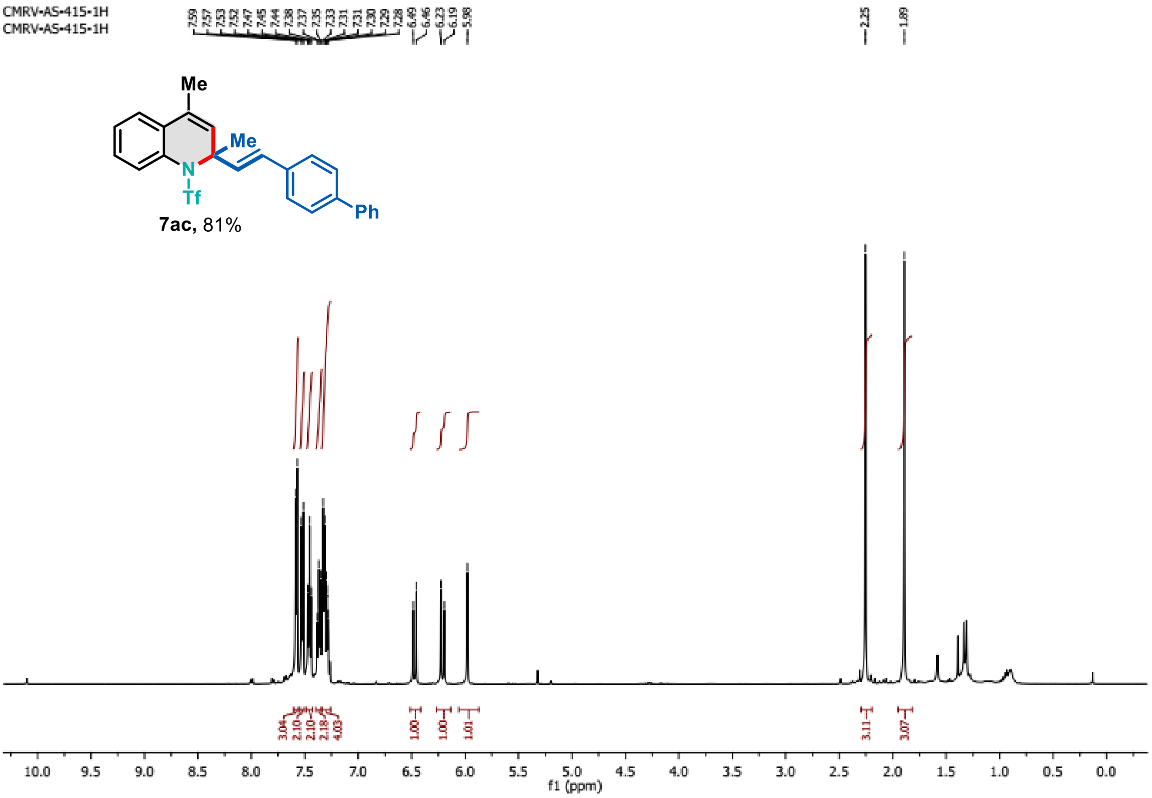
CMRV-AS-415-1H
CMRV-AS-415-1H
Me
Me
N
Tf
Ph
f1 (ppm)

7ac, 81%

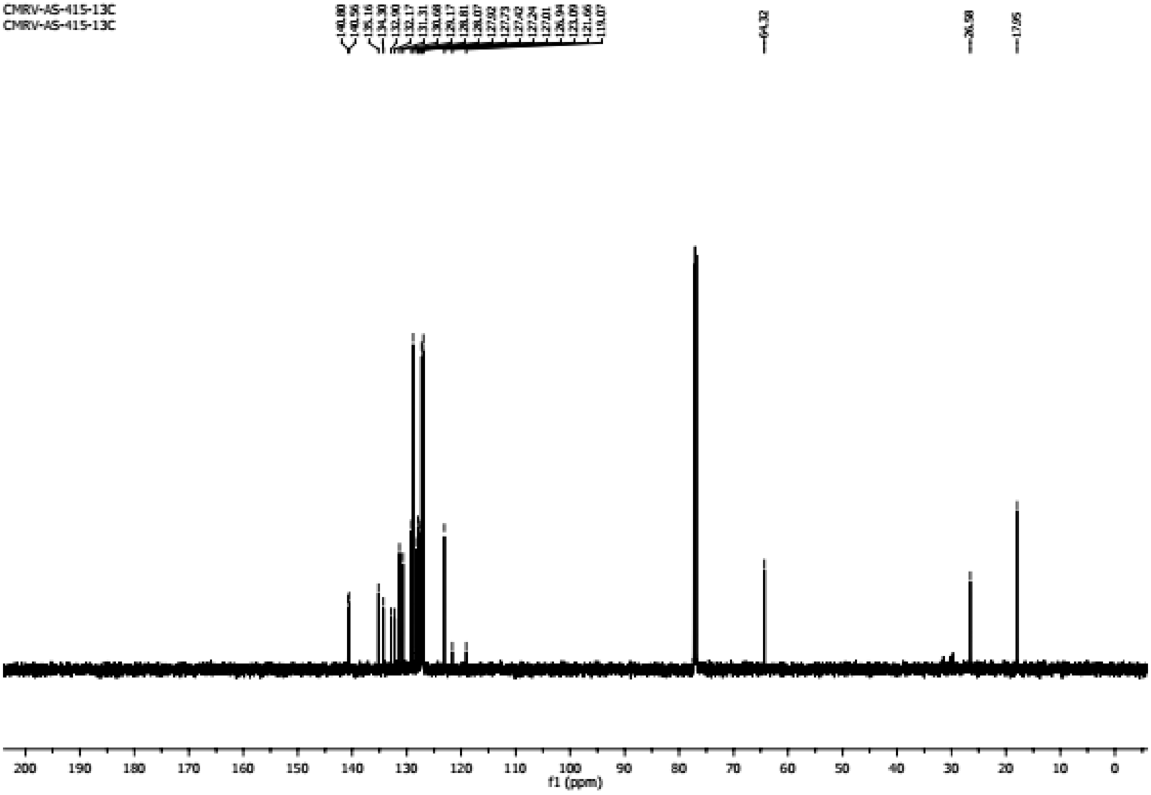
CMRV-AS-415-13C
CMRV-AS-415-13C
f1 (ppm)

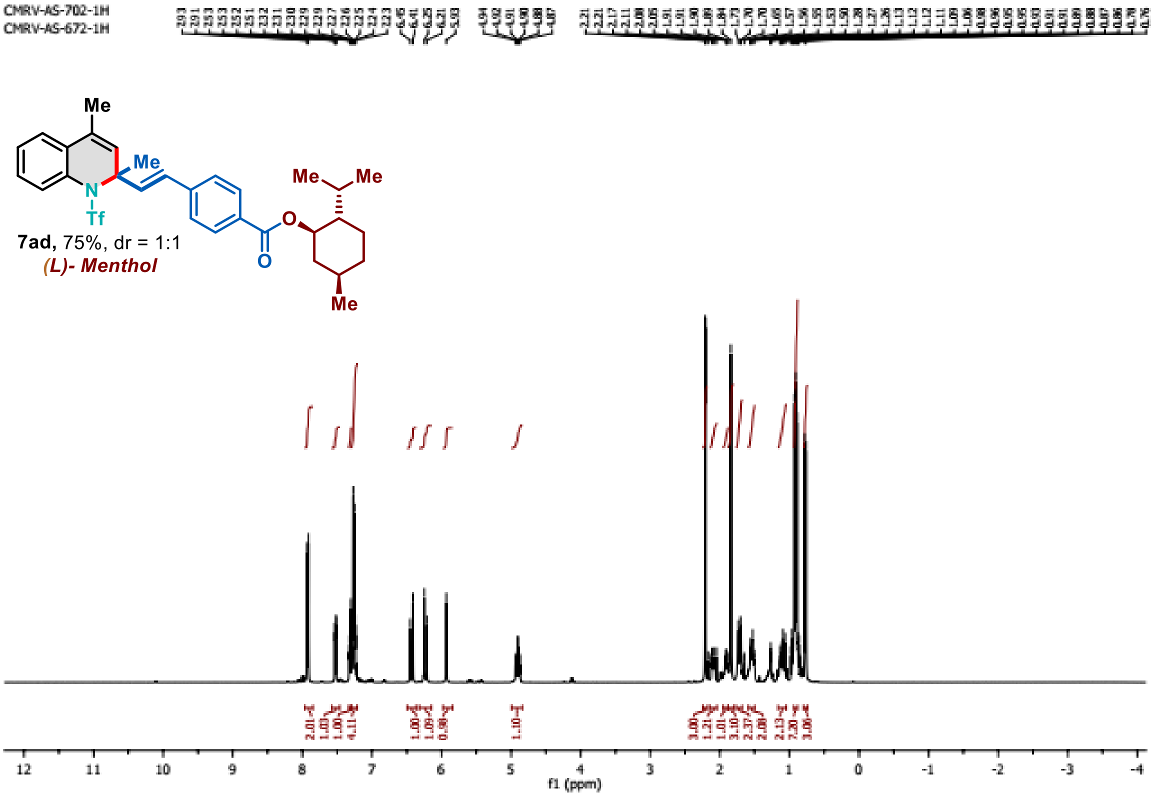
CMRV-AS-702-1H
CMRV-AS-672-1H
Me
Me
N
Tf
Me
Me
O
O
Me
7ad, 75%, dr = 1:1
(L)- Menthol
f1 (ppm)

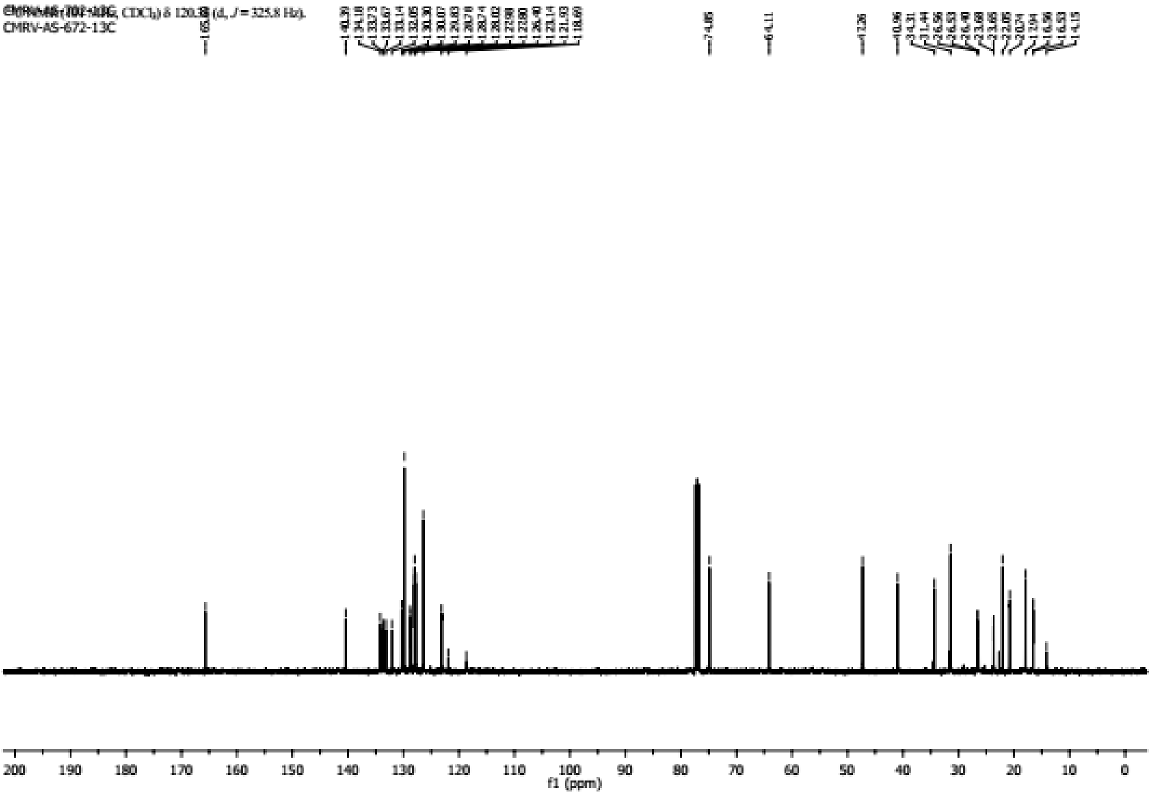
CMRV-AS-672-13C
f1 (ppm)

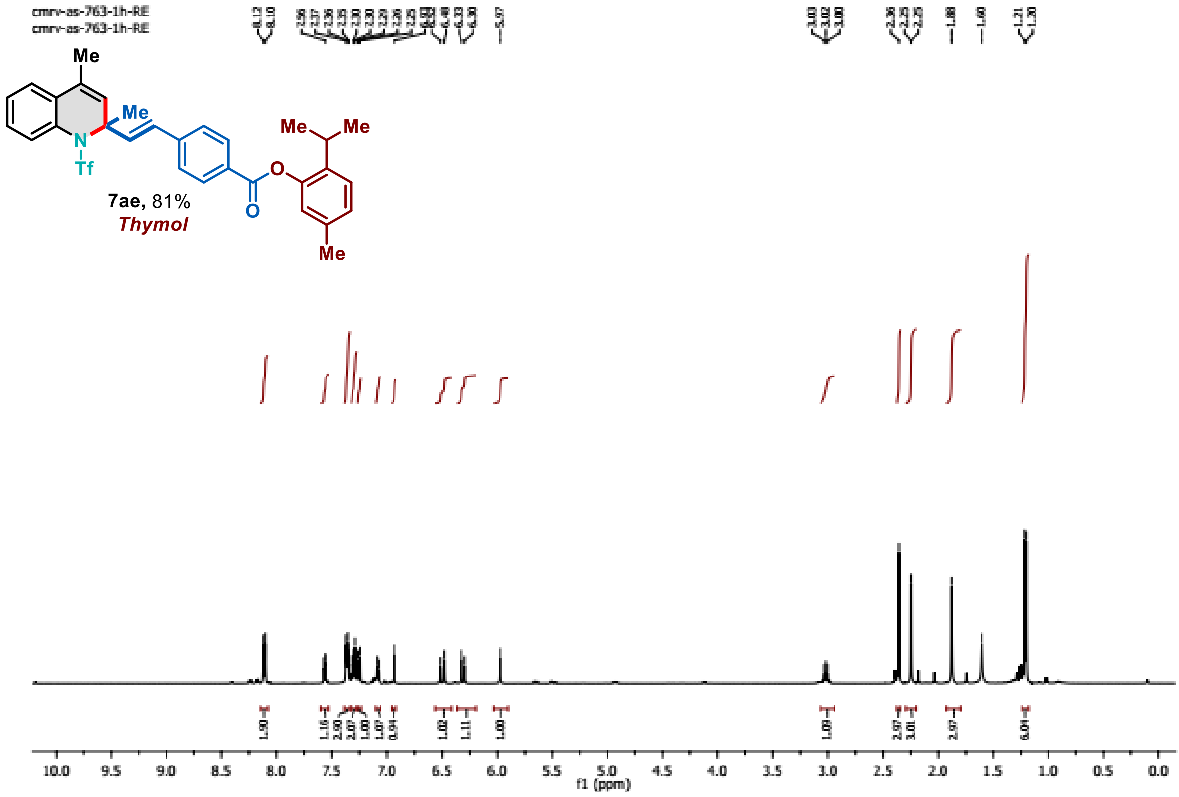
cmrv-as-763-1h-RE
cmrv-as-763-1h-RE
Me
Me
N
Tf
Me
Me
O
O
Me
7ae, 81%
Thymol
8.12
8.10
6.48
6.33
6.30
5.97
3.03
3.02
3.00
2.36
2.25
1.88
1.60
1.21
1.20
1.90
1.16
2.90
2.07
1.00
1.07
0.94
1.02
1.11
1.00
1.09
2.97
3.01
2.97
6.04
10.0 9.5 9.0 8.5 8.0 7.5 7.0 6.5 6.0 5.5 5.0 4.5 4.0 3.5 3.0 2.5 2.0 1.5 1.0 0.5 0.0
f1 (ppm)

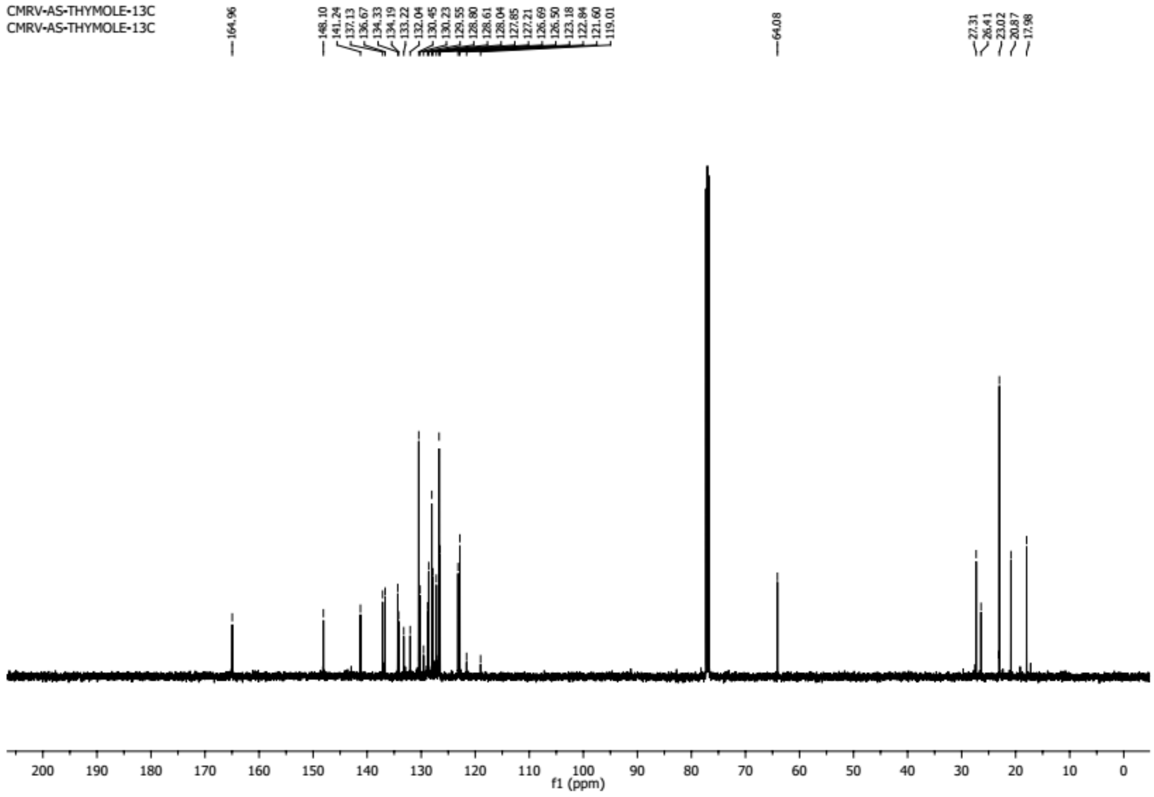
CMRV-AS-THYMOLE-13C
CMRV-AS-THYMOLE-13C
164.96
148.10
141.24
137.13
136.67
134.33
134.19
133.22
132.04
130.45
130.23
129.55
128.80
128.61
128.04
127.85
127.21
126.69
126.50
123.18
122.84
121.60
119.01
64.08
27.31
26.41
23.02
20.87
17.98
200 190 180 170 160 150 140 130 120 110 100 90 80 70 60 50 40 30 20 10 0
f1 (ppm)

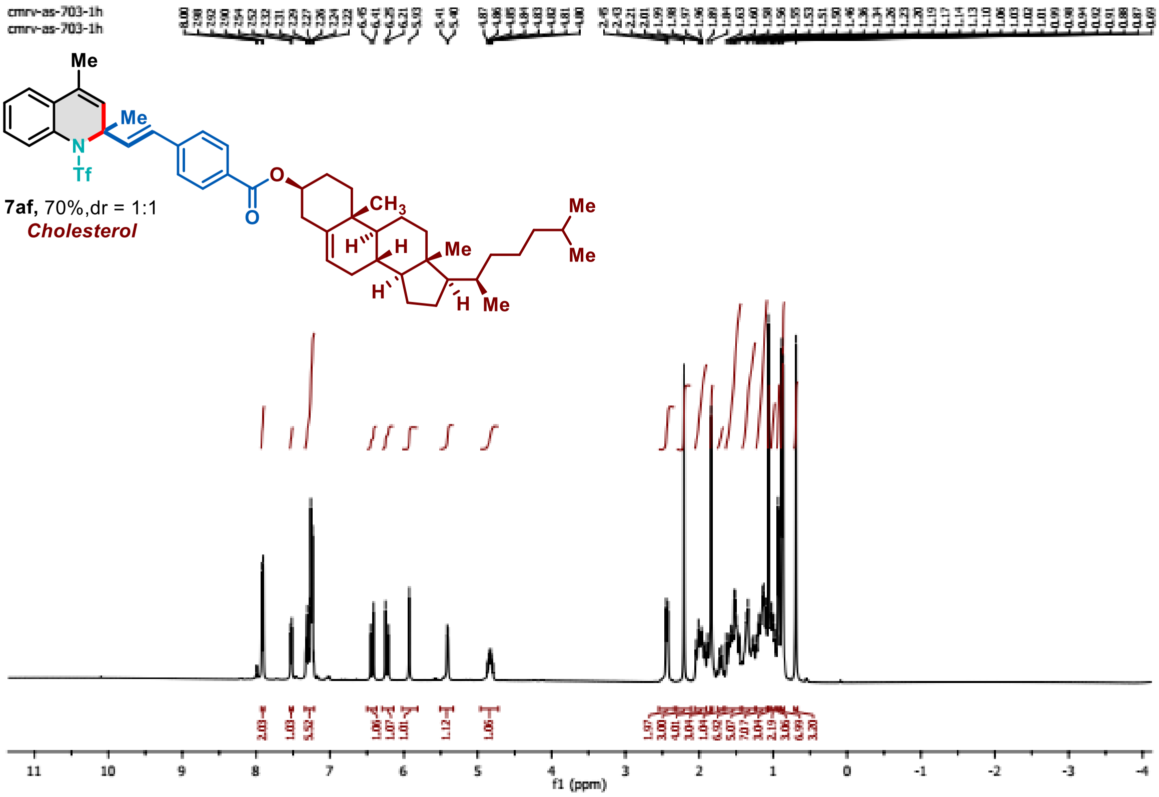
cmrv-as-703-1h
cmrv-as-703-1h
Me
Me
N
Tf
O
O
CH3
H
H
H
Me
H
Me
Me
Me
7af, 70%,dr = 1:1
Cholesterol
f1 (ppm)

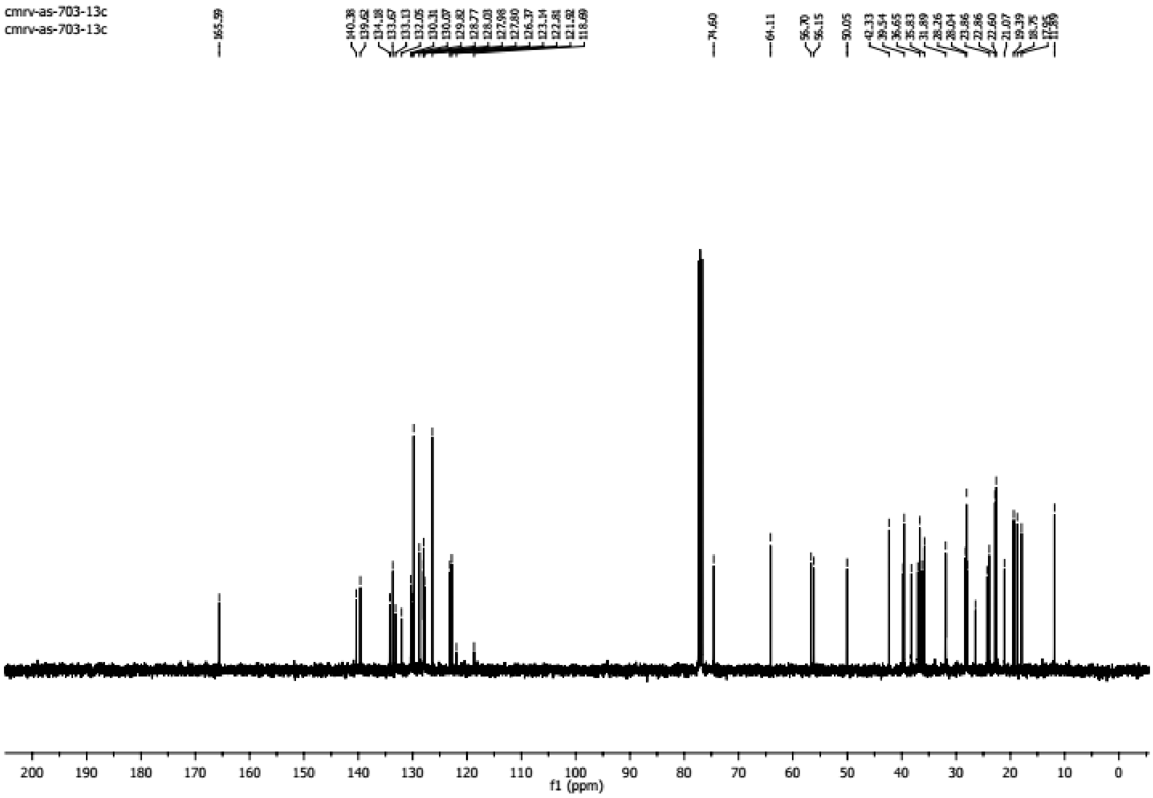
cmrv-as-703-13c
cmrv-as-703-13c
f1 (ppm)

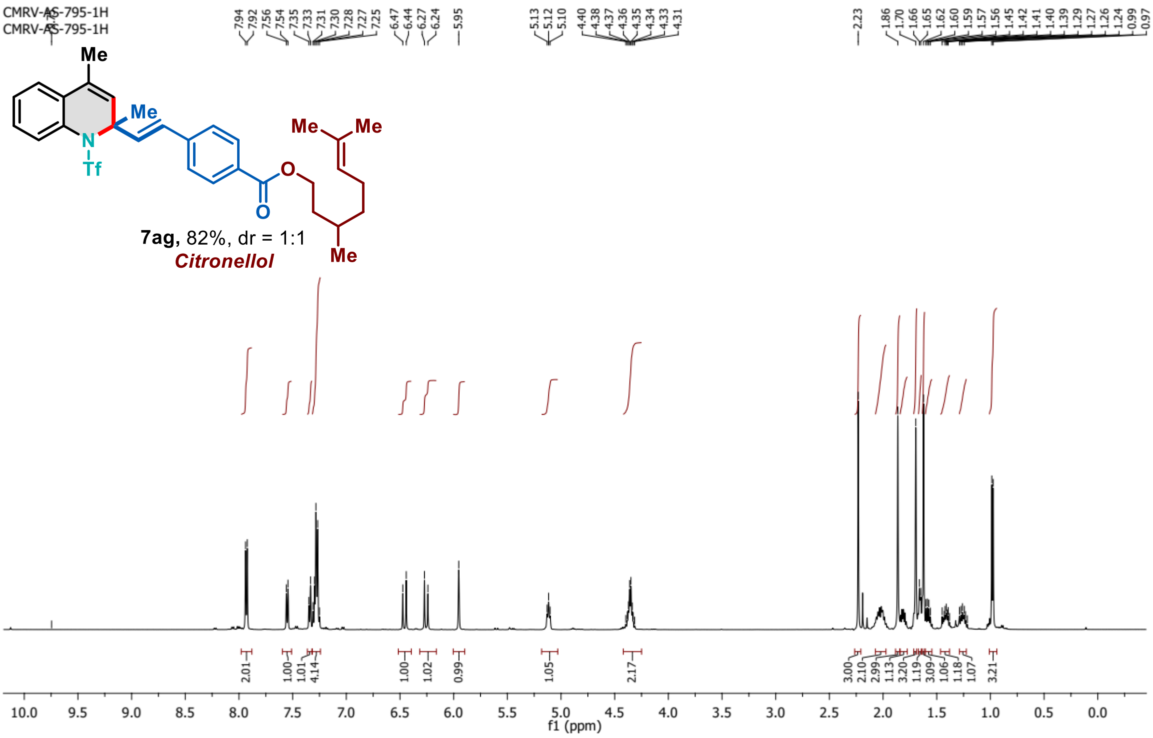
CMRV-AS-795-1H
CMRV-AS-795-1H
7.94 7.92 7.56 7.54 7.35 7.33 7.31 7.30 7.28 7.27 7.25 6.47 6.44 6.27 6.24 5.95 5.13 5.12 5.10 4.40 4.38 4.37 4.36 4.35 4.34 4.33 4.31 2.23 1.86 1.70 1.66 1.65 1.62 1.60 1.59 1.57 1.56 1.45 1.42 1.41 1.40 1.39 1.29 1.27 1.26 1.24 0.99 0.97
Me
Me
N
Tf
Me
Me
O
O
Me
7ag, 82%, dr = 1:1
Citronellol
2.01 1.00 1.01 4.14 1.00 1.02 0.99 1.05 2.17 3.00 2.10 2.99 1.13 3.20 1.19 3.09 1.06 1.18 1.07 3.21
f1 (ppm)

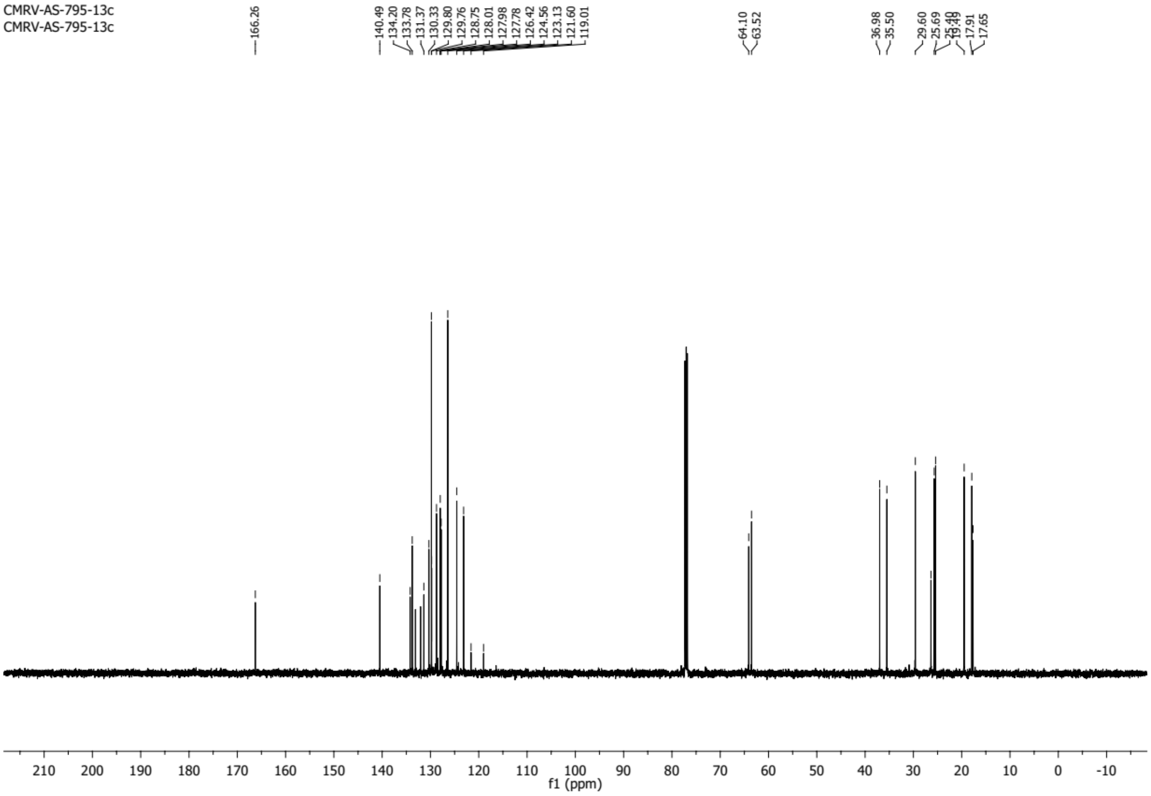
CMRV-AS-795-13c
CMRV-AS-795-13c
166.26
140.49 134.20 133.78 131.37 130.33 129.80 129.76 128.75 128.01 127.98 127.78 126.42 124.56 123.13 121.60 119.01
64.10 63.52
36.98 35.50
29.60 25.69 17.91 17.65
f1 (ppm)

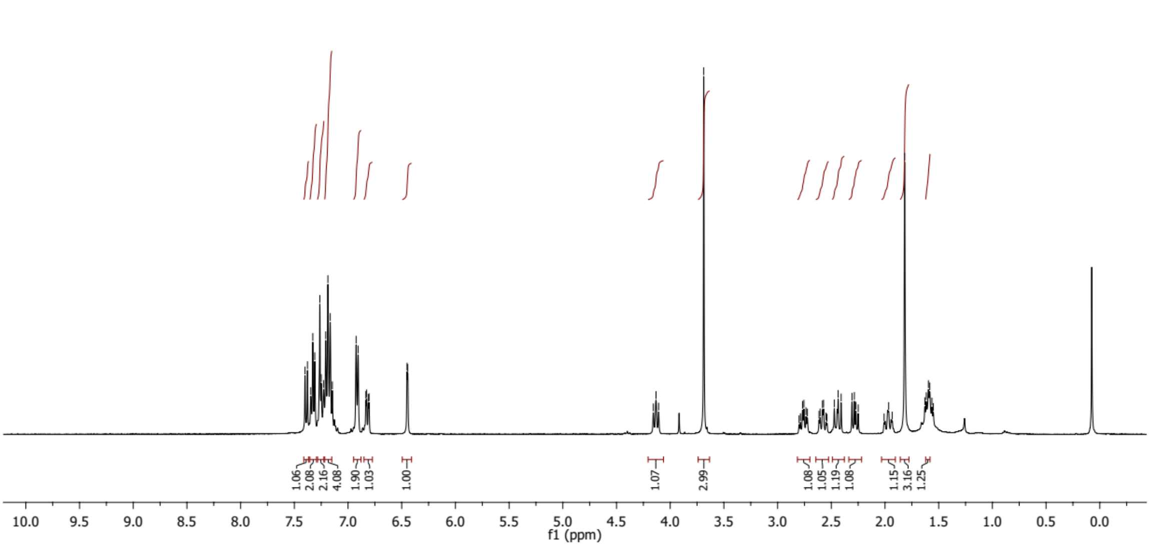
CMRV-AS-1H
CMRV-AS-1H
f1 (ppm)

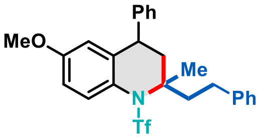
MeO
Ph
Me
Ph
N
Tf

8, 82%

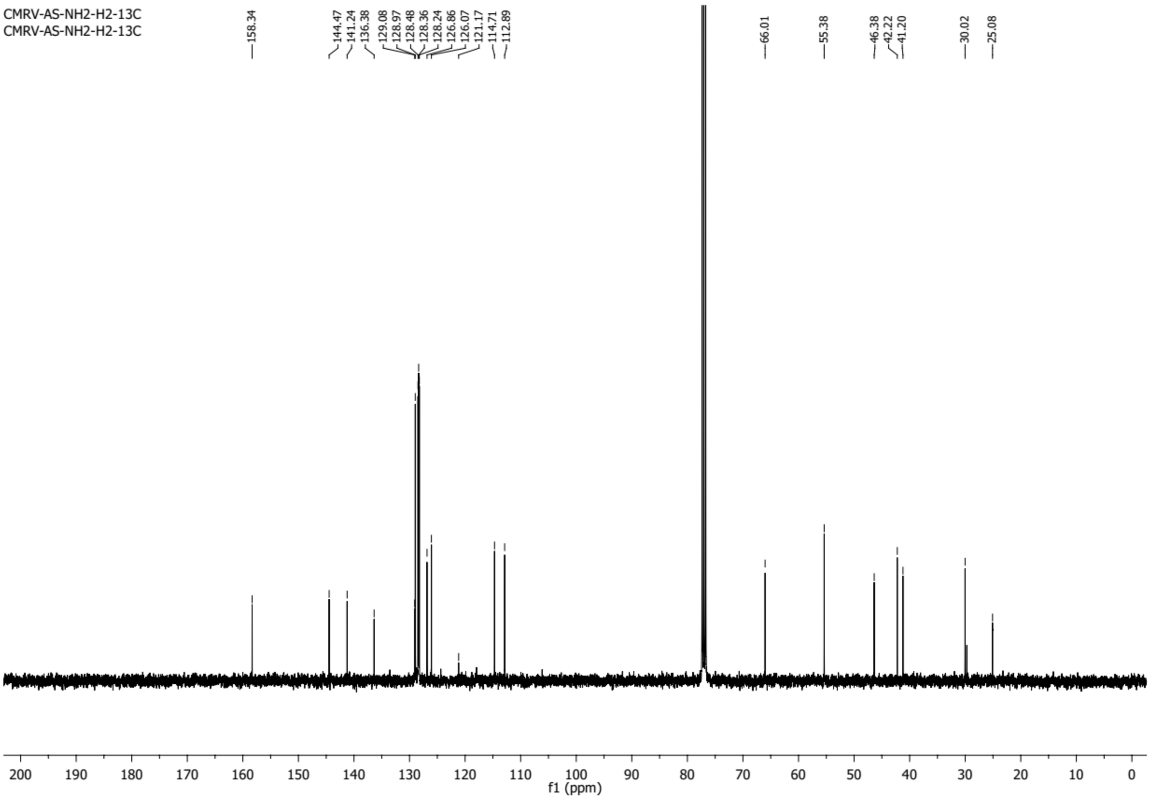
CMRV-AS-NH2-H2-13C
CMRV-AS-NH2-H2-13C
158.34
144.47
141.24
136.38
66.01
55.38
46.38
42.22
41.20
30.02
25.08
f1 (ppm)

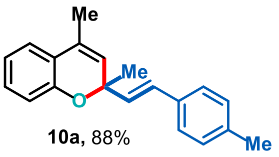
Me
Me
O
Me

10a, 88%

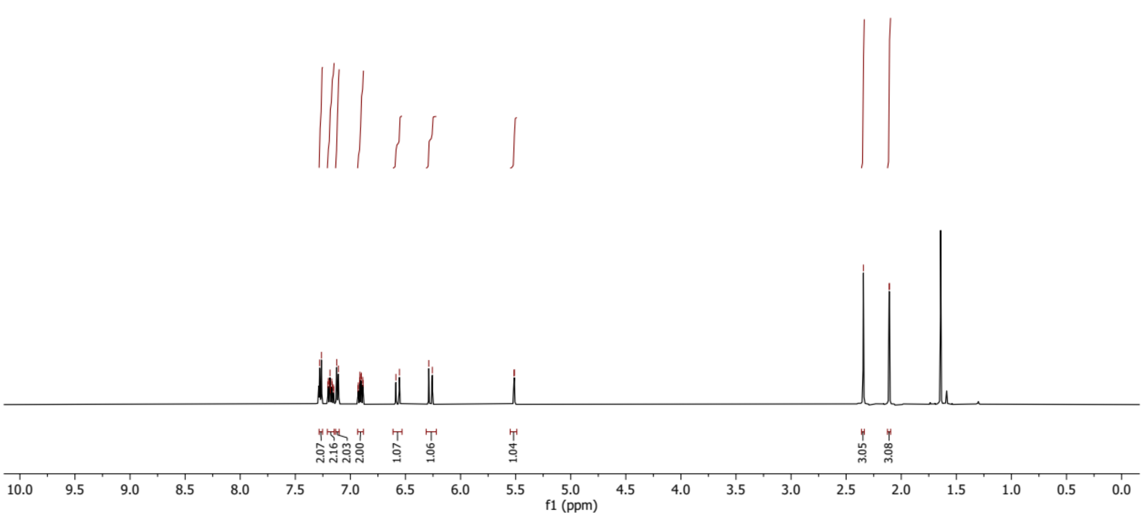
AS-378-1H.1.fid
CMRV-AS-378-1H
7.20 7.20 7.19 7.18 7.18 7.17 7.17 7.15 7.15 7.12 7.11 6.93 6.93 6.92 6.91 6.90 6.90 6.89 6.89 6.59 6.55 6.29 6.26 5.51 5.51
2.34 2.11 2.11
2.07 2.16 2.03 2.00 1.07 1.06 1.04 3.05 3.08
10.0 9.5 9.0 8.5 8.0 7.5 7.0 6.5 6.0 5.5 5.0 4.5 4.0 3.5 3.0 2.5 2.0 1.5 1.0 0.5 0.0
f1 (ppm)

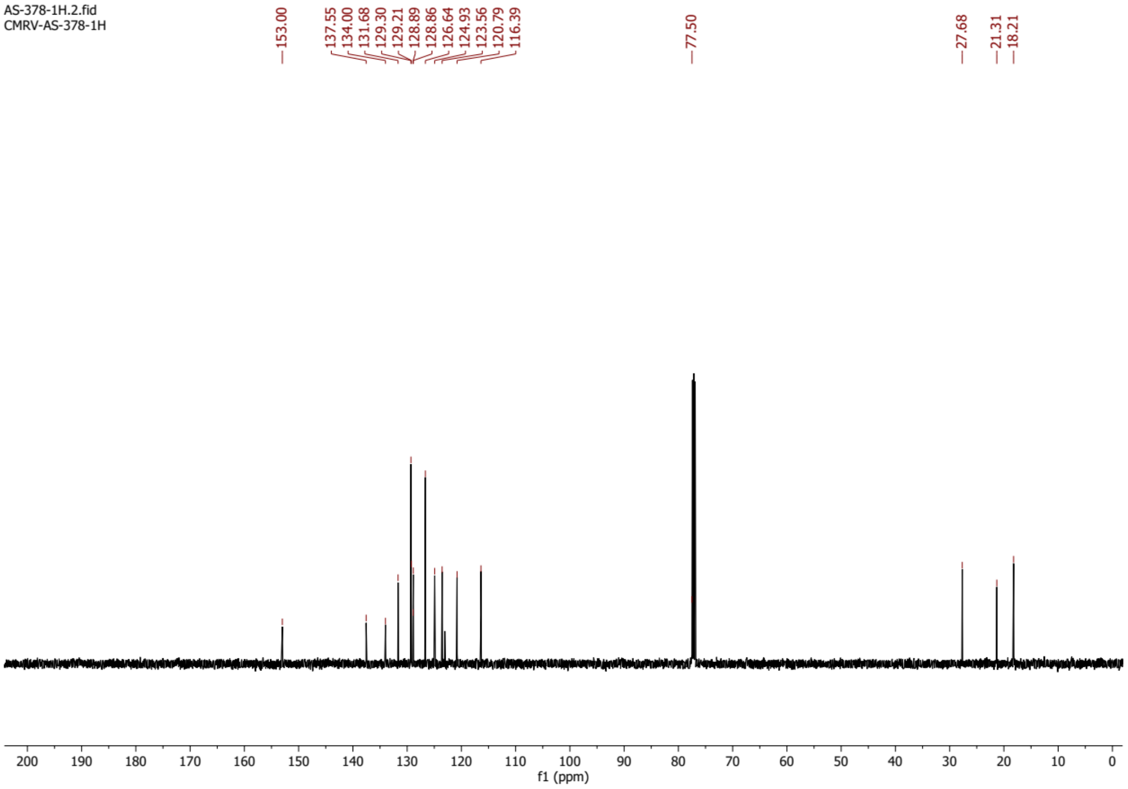
AS-378-1H.2.fid
CMRV-AS-378-1H
153.00
137.55 134.00 131.68 129.30 129.21 128.89 128.86 126.64 124.93 123.56 120.79 116.39
77.50
27.68 21.31 18.21
200 190 180 170 160 150 140 130 120 110 100 90 80 70 60 50 40 30 20 10 0
f1 (ppm)

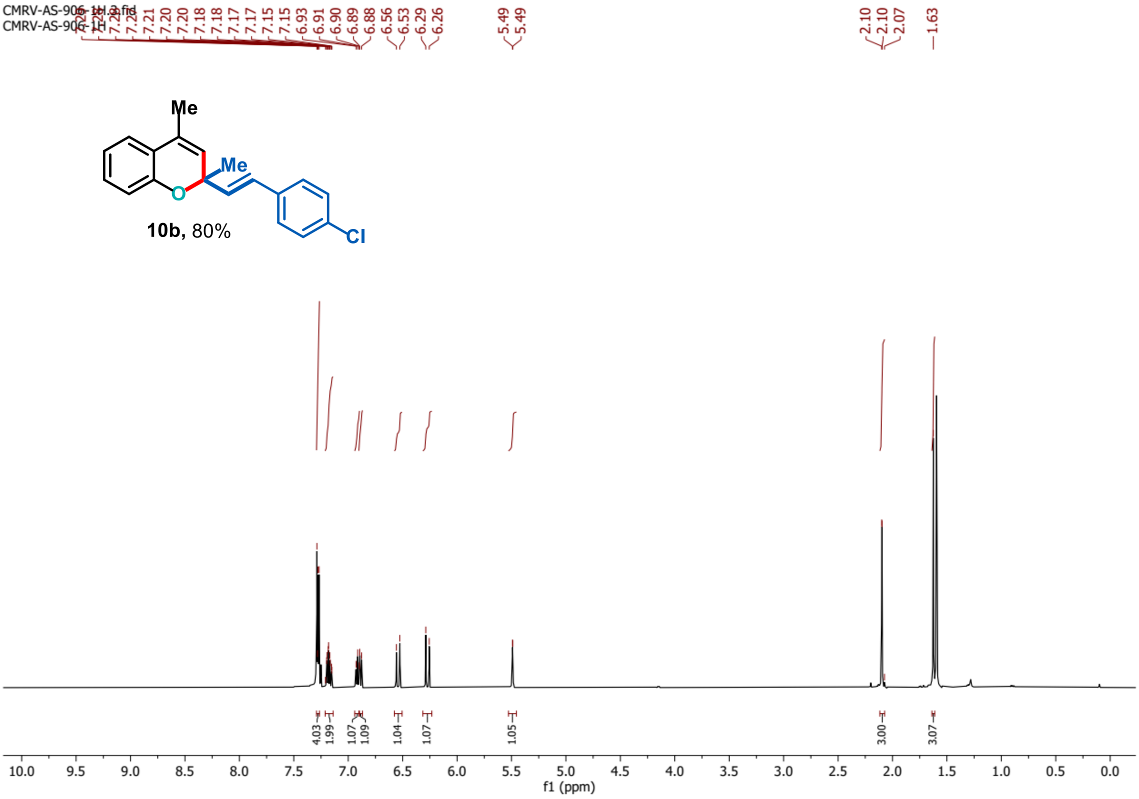
CMRV-AS-906-1H.3.fid
CMRV-AS-906-1H
7.21
7.20
7.20
7.18
7.18
7.17
7.17
7.15
7.15
6.93
6.91
6.90
6.89
6.88
6.56
6.53
6.29
6.26
5.49
5.49
2.10
2.10
2.07
1.63
Me
Me
O
Cl
10b, 80%
4.03
1.99
1.07
1.09
1.04
1.07
1.05
3.00
3.07
f1 (ppm)

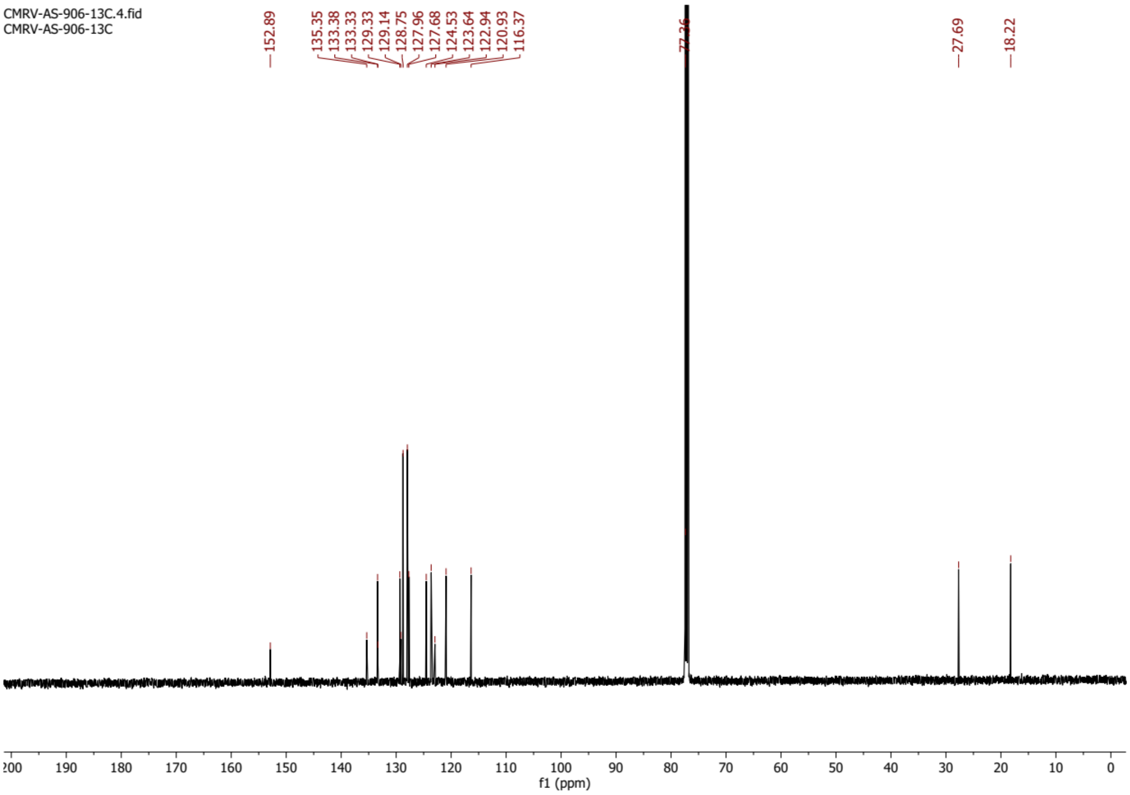
CMRV-AS-906-13C.4.fid
CMRV-AS-906-13C
152.89
135.35
133.38
133.33
129.33
129.14
128.75
127.96
127.68
124.53
123.64
122.94
120.93
116.37
27.69
18.22
f1 (ppm)

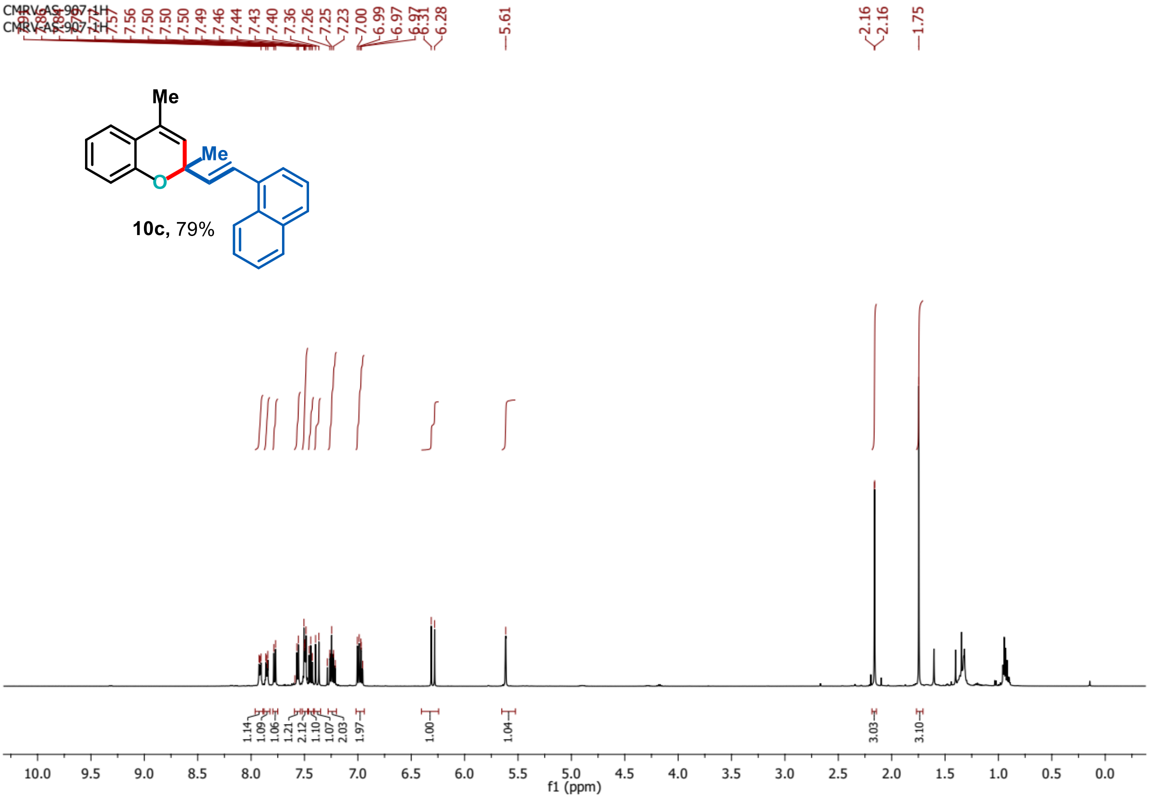
CMRV-AS-907-1H
7.57
7.56
7.50
7.50
7.50
7.49
7.46
7.44
7.43
7.40
7.36
7.26
7.25
7.23
7.00
6.99
6.97
6.31
6.28
5.61
2.16
2.16
1.75
Me
Me
O
10c, 79%
1.14
1.09
1.06
1.21
2.12
1.10
1.07
2.03
1.97
1.00
1.04
3.03
3.10
f1 (ppm)

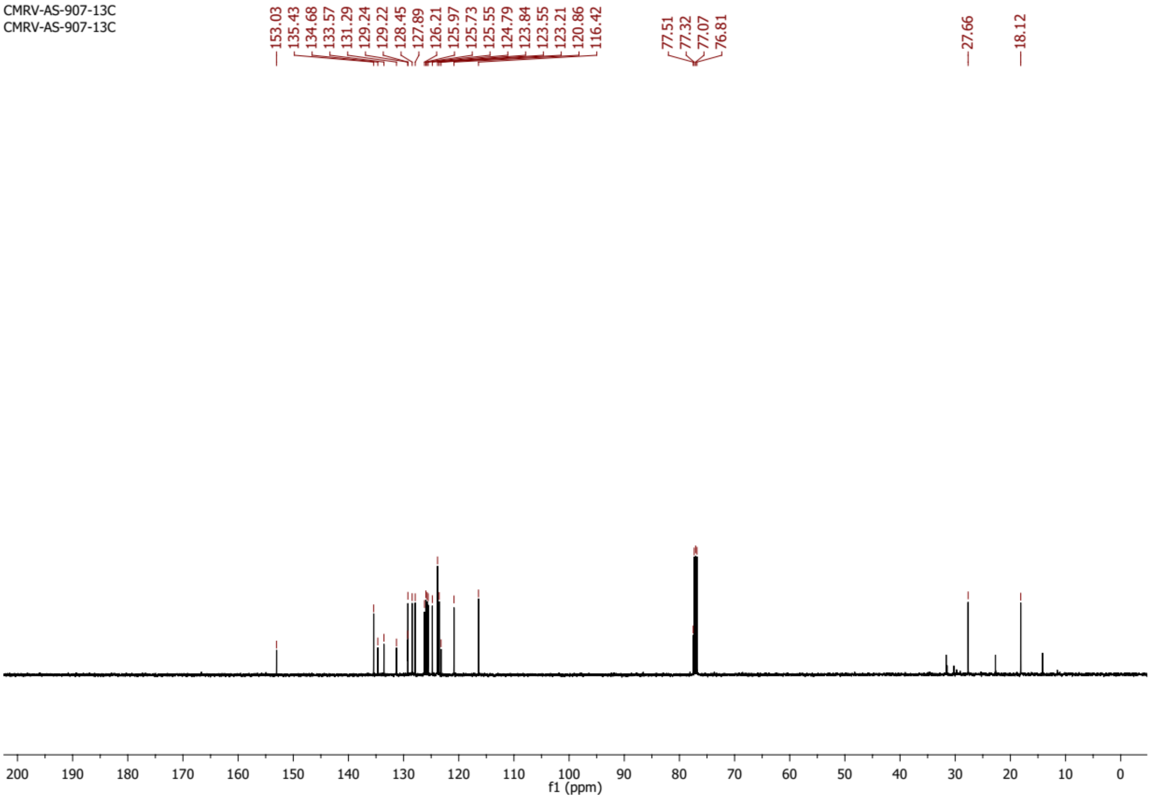
CMRV-AS-907-13C
CMRV-AS-907-13C
153.03
135.43
134.68
133.57
131.29
129.24
129.22
128.45
127.89
126.21
125.97
125.73
125.55
124.79
123.84
123.55
123.21
120.86
116.42
77.51
77.32
77.07
76.81
27.66
18.12
f1 (ppm)

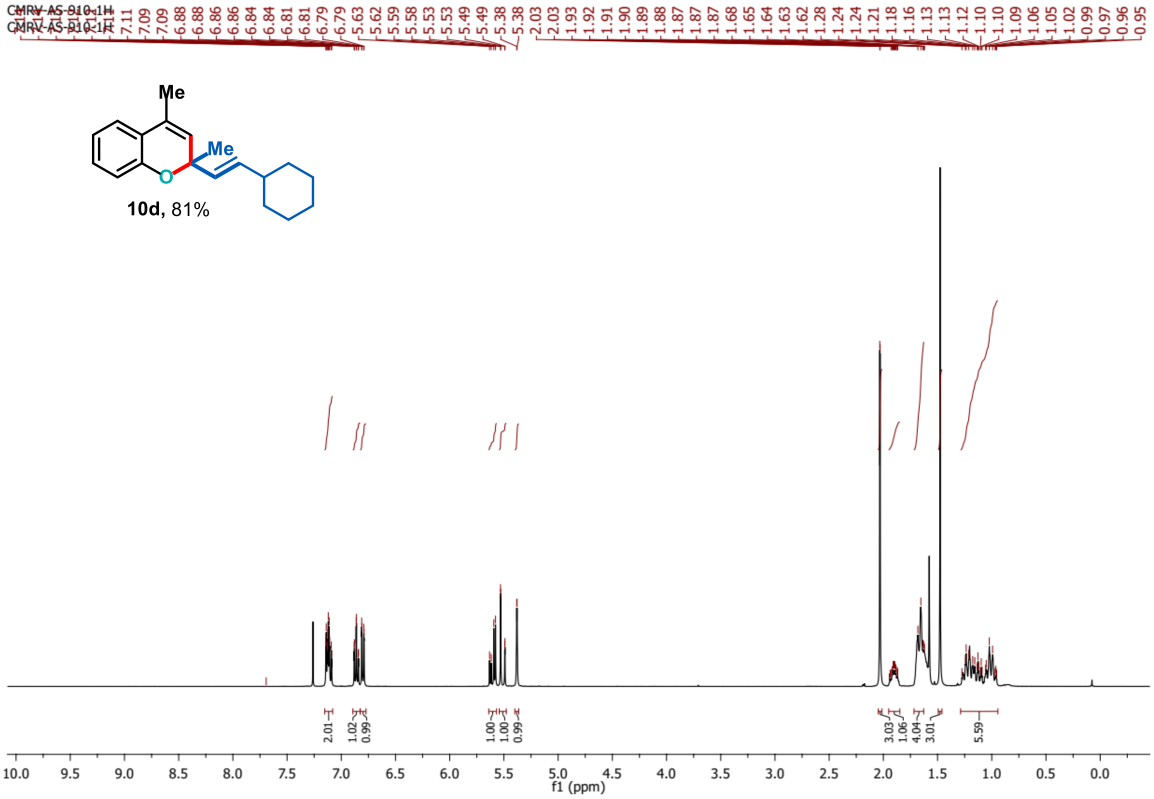
CMRV-AS-910-1H
CMRV-AS-910-1H
7.11 7.09 7.09 6.88 6.88 6.86 6.86 6.84 6.84 6.81 6.81 6.79 6.79 5.63 5.62 5.59 5.58 5.53 5.53 5.49 5.49 5.38 5.38 2.03 2.03 1.93 1.92 1.91 1.90 1.89 1.88 1.87 1.87 1.68 1.65 1.64 1.63 1.62 1.28 1.24 1.24 1.21 1.18 1.16 1.13 1.13 1.12 1.10 1.10 1.09 1.06 1.05 1.02 0.99 0.97 0.96 0.95
Me
Me
O
10d, 81%
2.01 1.02 0.99 1.00 1.00 0.99 3.03 1.06 4.04 3.01 5.59
10.0 9.5 9.0 8.5 8.0 7.5 7.0 6.5 6.0 5.5 5.0 4.5 4.0 3.5 3.0 2.5 2.0 1.5 1.0 0.5 0.0
f1 (ppm)

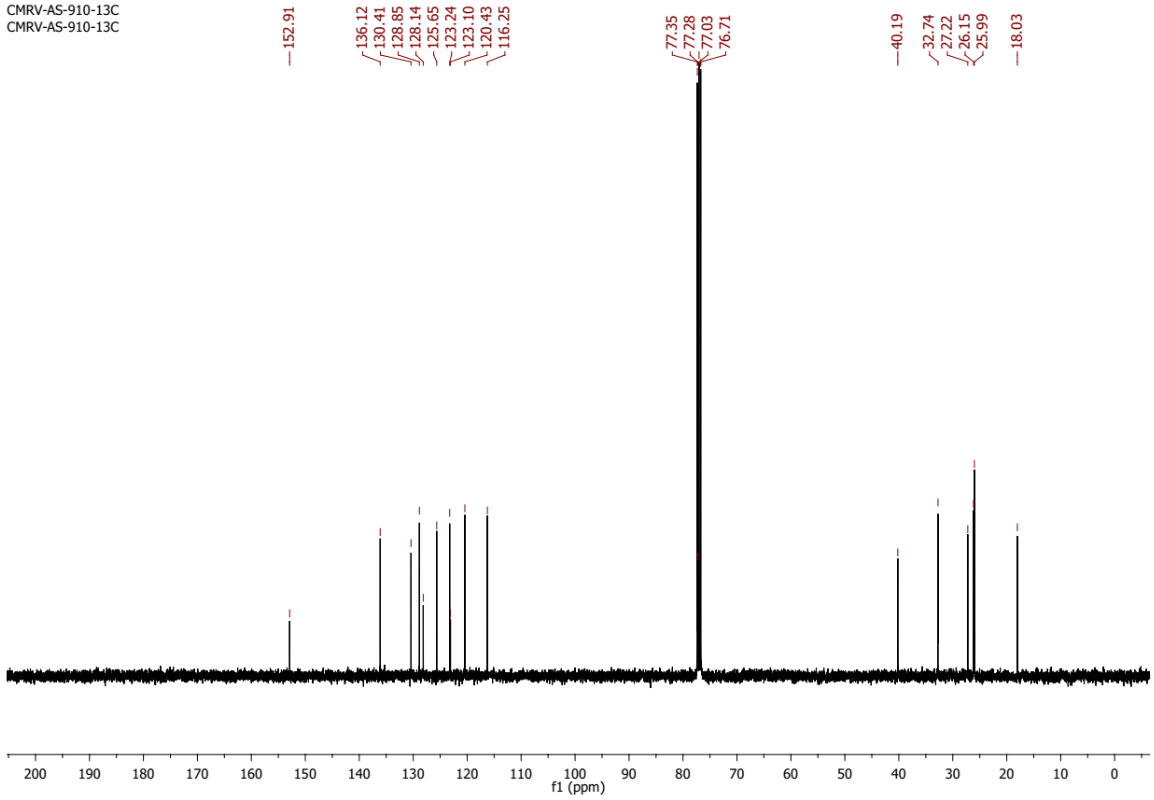
CMRV-AS-910-13C
CMRV-AS-910-13C
152.91 136.12 130.41 128.85 128.14 125.65 123.24 123.10 120.43 116.25 77.35 77.28 77.03 76.71 40.19 32.74 27.22 26.15 25.99 18.03
200 190 180 170 160 150 140 130 120 110 100 90 80 70 60 50 40 30 20 10 0
f1 (ppm)

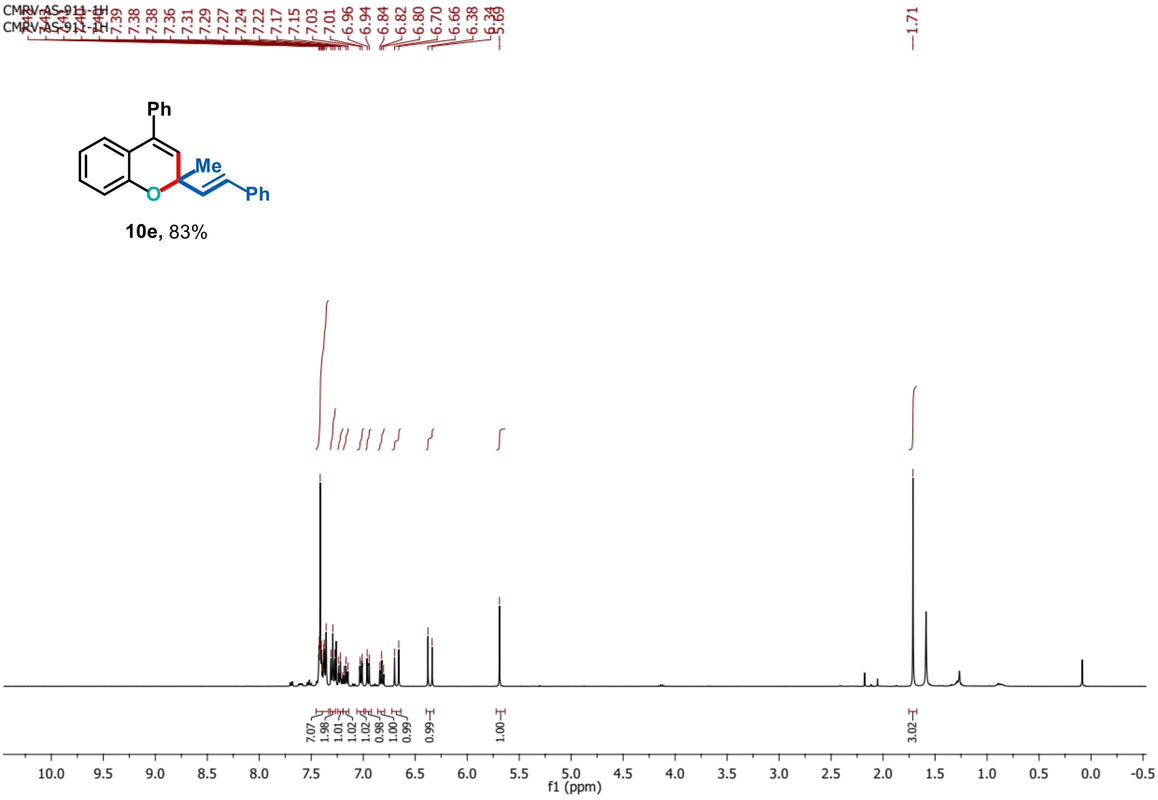
CMRV-AS-911-1H
7.39
7.38
7.38
7.36
7.31
7.29
7.27
7.24
7.22
7.17
7.15
7.03
7.01
6.96
6.94
6.84
6.82
6.80
6.70
6.66
6.38
6.34
5.69
1.71
Ph
Me
O
Ph
10e, 83%
7.07
1.98
1.01
1.02
0.98
1.00
0.99
0.99
1.00
3.02
f1 (ppm)

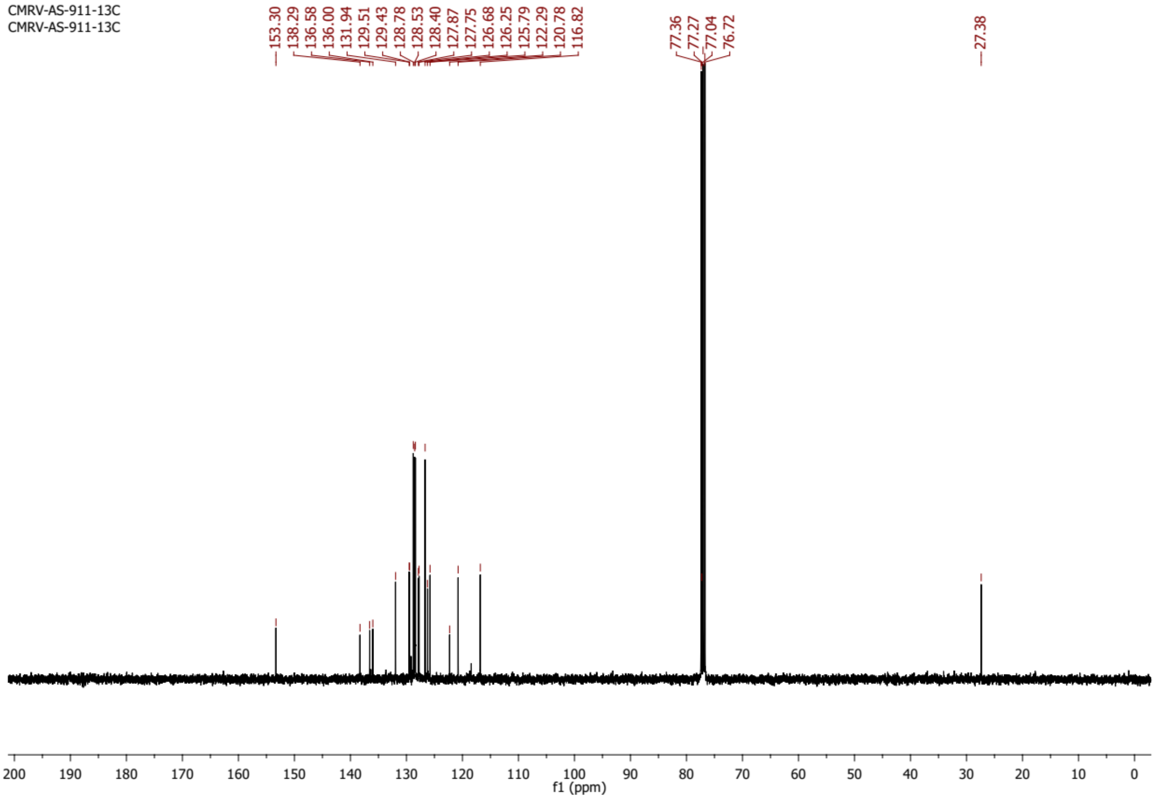
CMRV-AS-911-13C
CMRV-AS-911-13C
153.30
138.29
136.58
136.00
131.94
129.51
129.43
128.78
128.53
128.40
127.87
127.75
126.68
126.25
125.79
122.29
120.78
116.82
77.36
77.27
77.04
76.72
27.38
f1 (ppm)

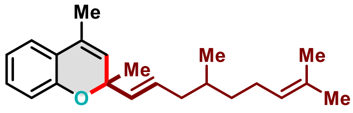

10f, 78%, dr = 1:1
Citronellal

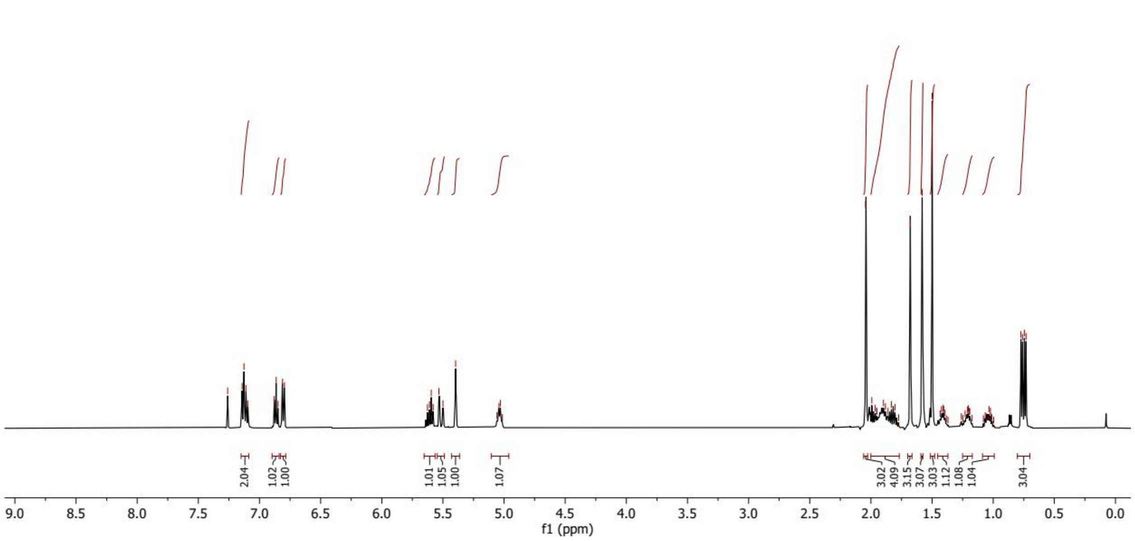

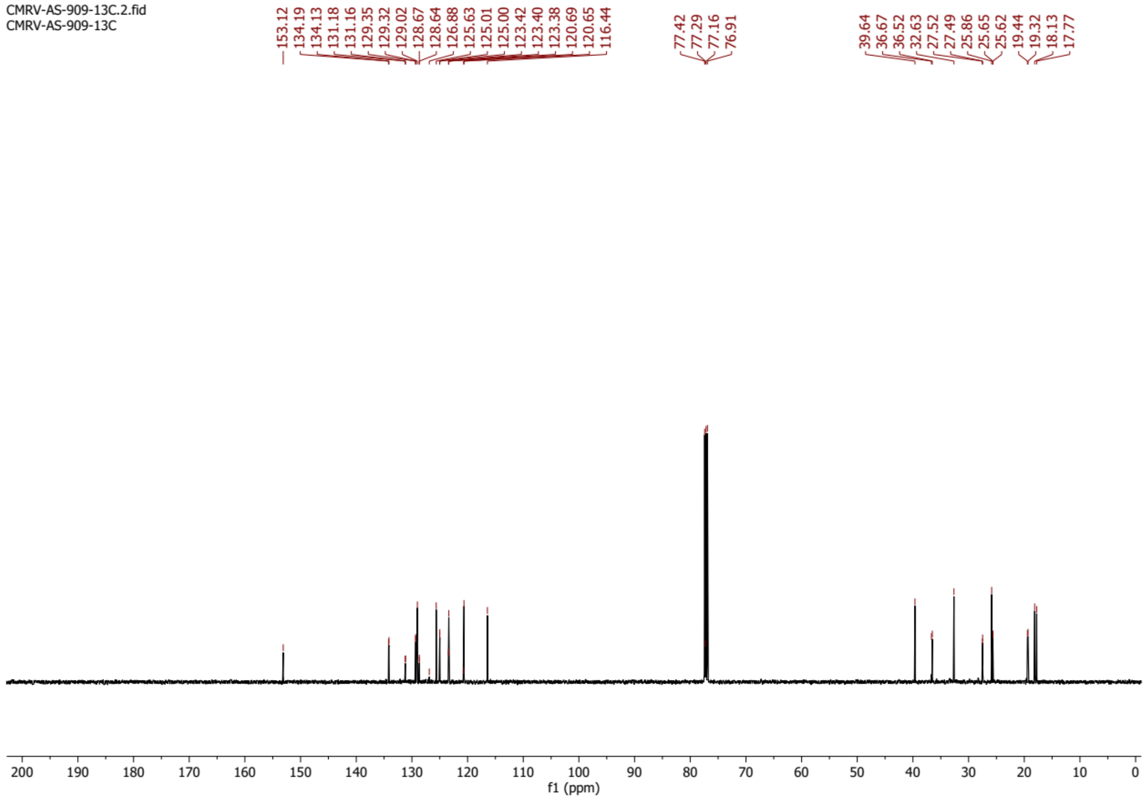

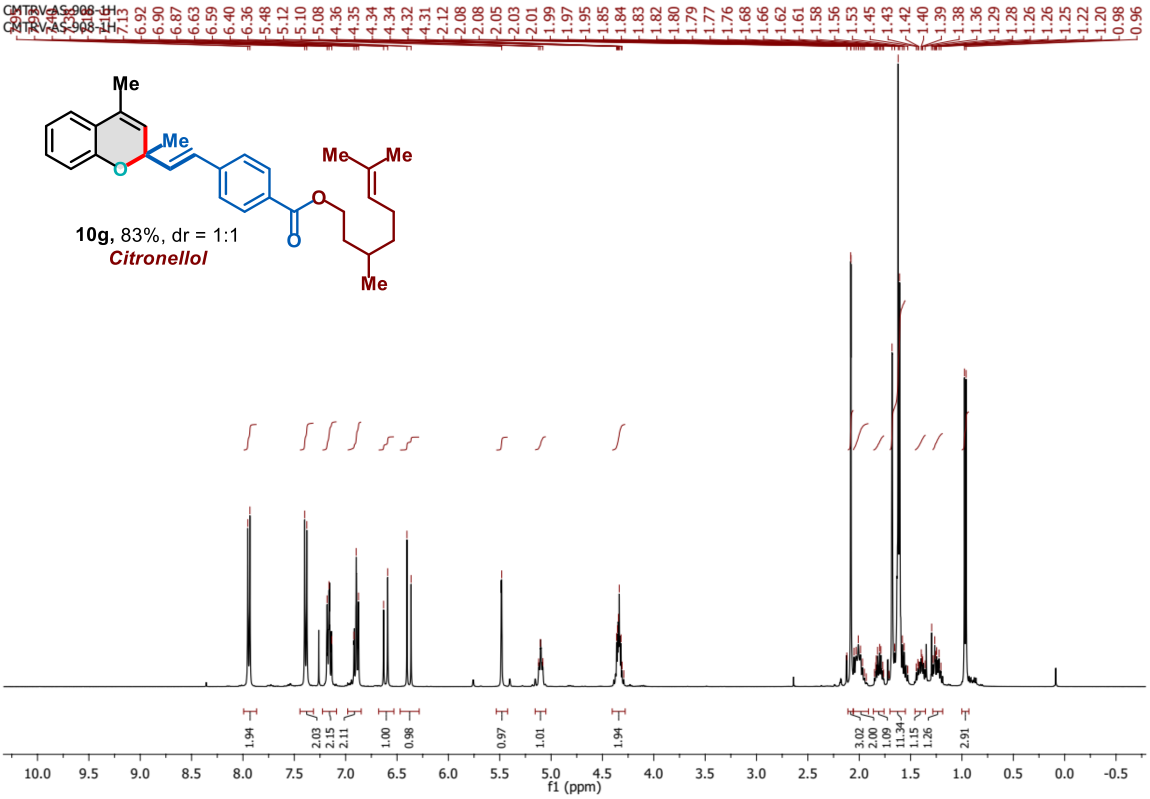
CMTRV-AS-908-1H
7.13
6.92
6.90
6.87
6.63
6.59
6.40
6.36
5.48
5.12
5.10
5.08
4.36
4.35
4.34
4.34
4.32
4.31
2.12
2.08
2.08
2.05
2.03
2.01
1.99
1.97
1.95
1.85
1.84
1.83
1.82
1.80
1.79
1.76
1.68
1.66
1.62
1.61
1.58
1.56
1.53
1.45
1.43
1.42
1.40
1.39
1.38
1.36
1.29
1.28
1.26
1.26
1.25
1.22
1.20
0.98
0.96
Me
Me
O
Me
Me
O
O
Me
10g, 83%, dr = 1:1
Citronellol
1.94
2.03
2.15
2.11
1.00
0.98
0.97
1.01
1.94
3.02
2.00
1.09
11.34
1.15
1.26
2.91
f1 (ppm)

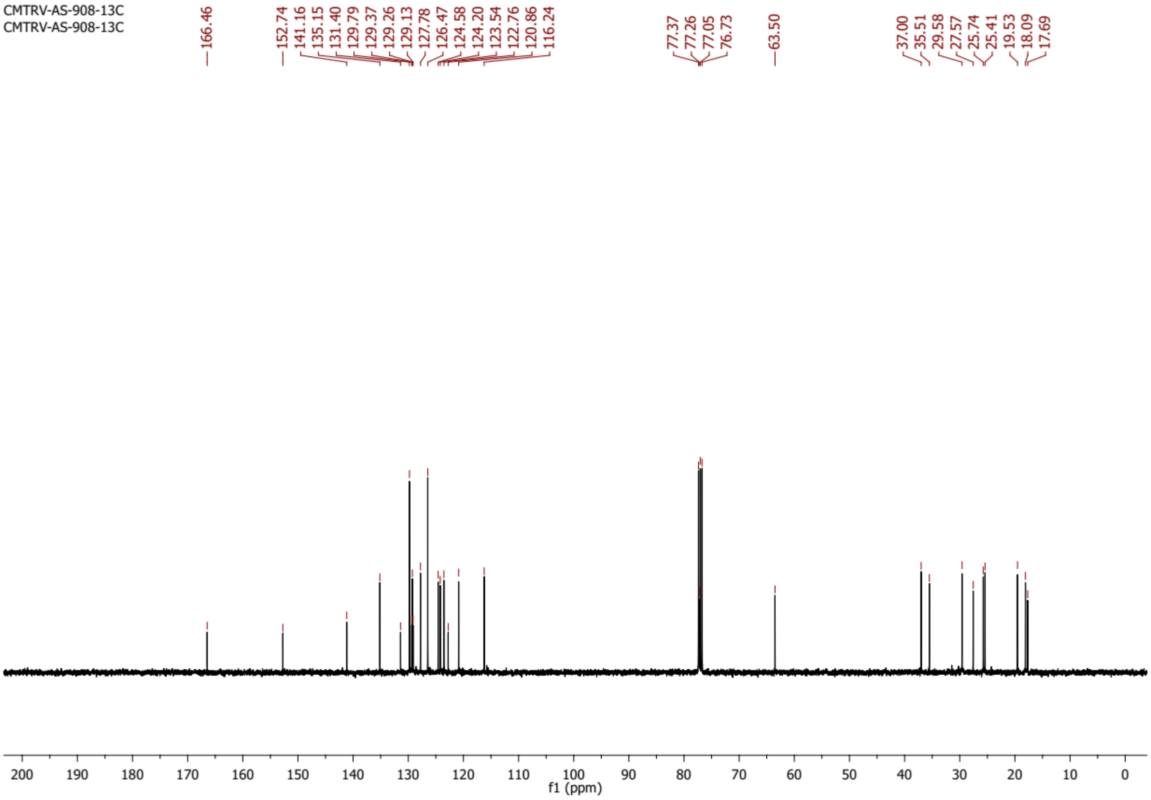
CMTRV-AS-908-13C
CMTRV-AS-908-13C
166.46
152.74
141.16
135.15
131.40
129.79
129.37
129.26
129.13
127.78
126.47
124.58
124.20
123.54
122.76
120.86
116.24
77.37
77.26
77.05
76.73
63.50
37.00
35.51
29.58
27.57
25.74
25.41
19.53
18.09
17.69
f1 (ppm)

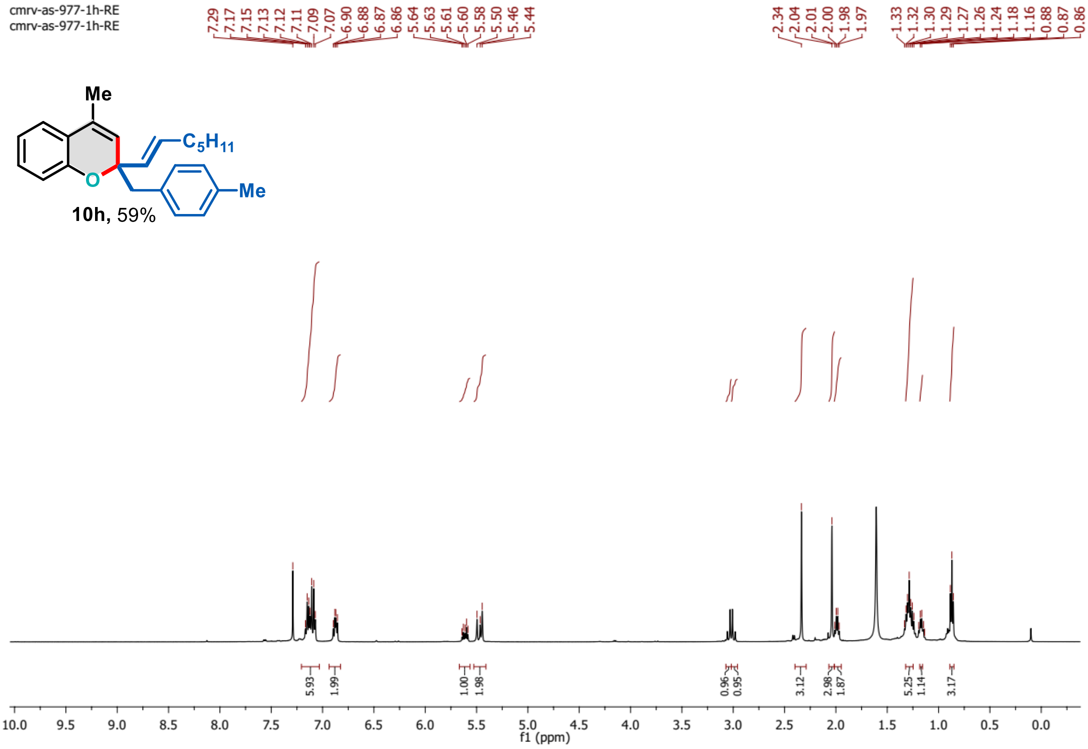
cmrv-as-977-1h-RE
cmrv-as-977-1h-RE
7.29
7.17
7.15
7.13
7.12
7.11
7.09
7.07
6.90
6.88
6.87
6.86
5.64
5.63
5.61
5.60
5.58
5.50
5.46
5.44
2.34
2.04
2.01
2.00
1.98
1.97
1.33
1.32
1.30
1.29
1.27
1.26
1.24
1.18
1.16
0.88
0.87
0.86
Me
C5H11
O
Me
5.93
1.99
1.00
1.98
0.96
0.95
3.12
2.98
1.87
5.25
1.14
3.17
f1 (ppm)

10h, 59%

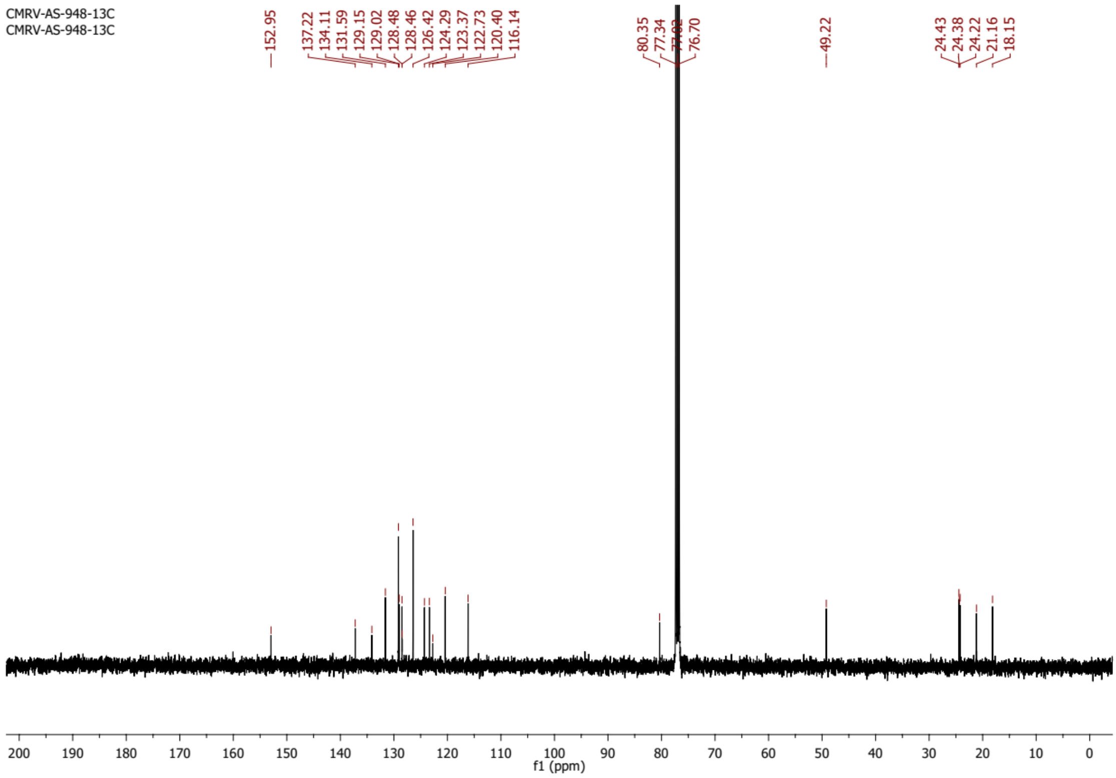
CMRV-AS-948-13C
CMRV-AS-948-13C
152.95
137.22
134.11
131.59
129.15
129.02
128.48
128.46
126.42
124.29
123.37
122.73
120.40
116.14
80.35
77.34
76.70
49.22
24.43
24.38
24.22
21.16
18.15
f1 (ppm)

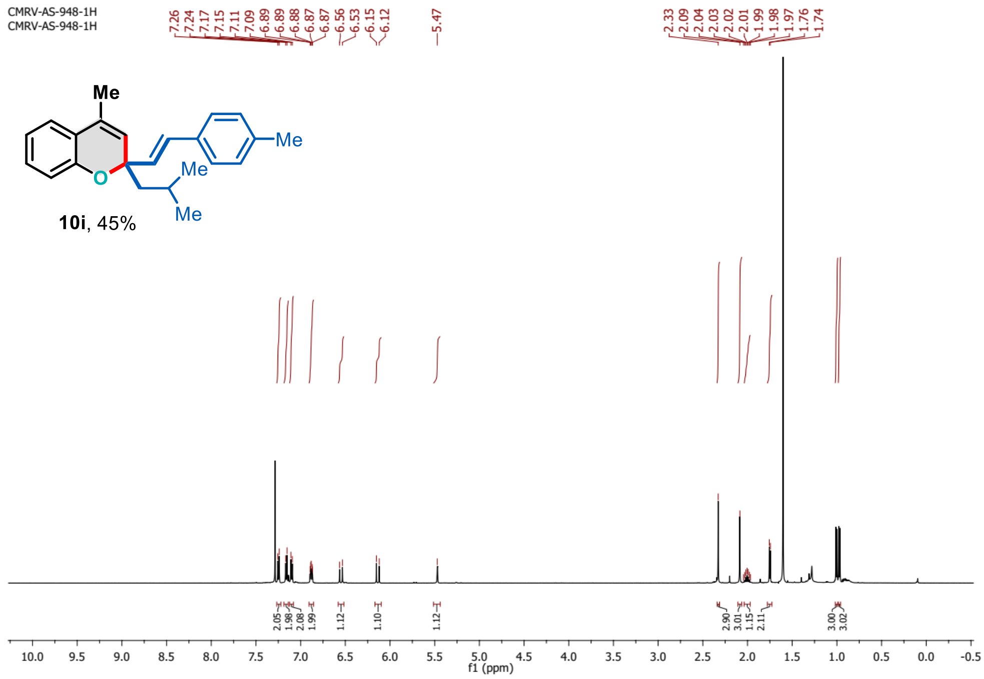
CMRV-AS-948-1H
CMRV-AS-948-1H
7.26 7.24 7.17 7.15 7.11 7.09 6.89 6.89 6.88 6.87 6.87 6.56 6.53 6.15 6.12
5.47
2.33 2.09 2.04 2.03 2.02 2.01 1.99 1.98 1.97 1.76 1.74
Me
O
Me
Me
Me
10i, 45%
2.05 1.98 2.08 1.99 1.12 1.10 1.12 2.90 3.01 1.15 2.11 3.00 3.02
10.0 9.5 9.0 8.5 8.0 7.5 7.0 6.5 6.0 5.5 5.0 4.5 4.0 3.5 3.0 2.5 2.0 1.5 1.0 0.5 0.0 -0.5
f1 (ppm)

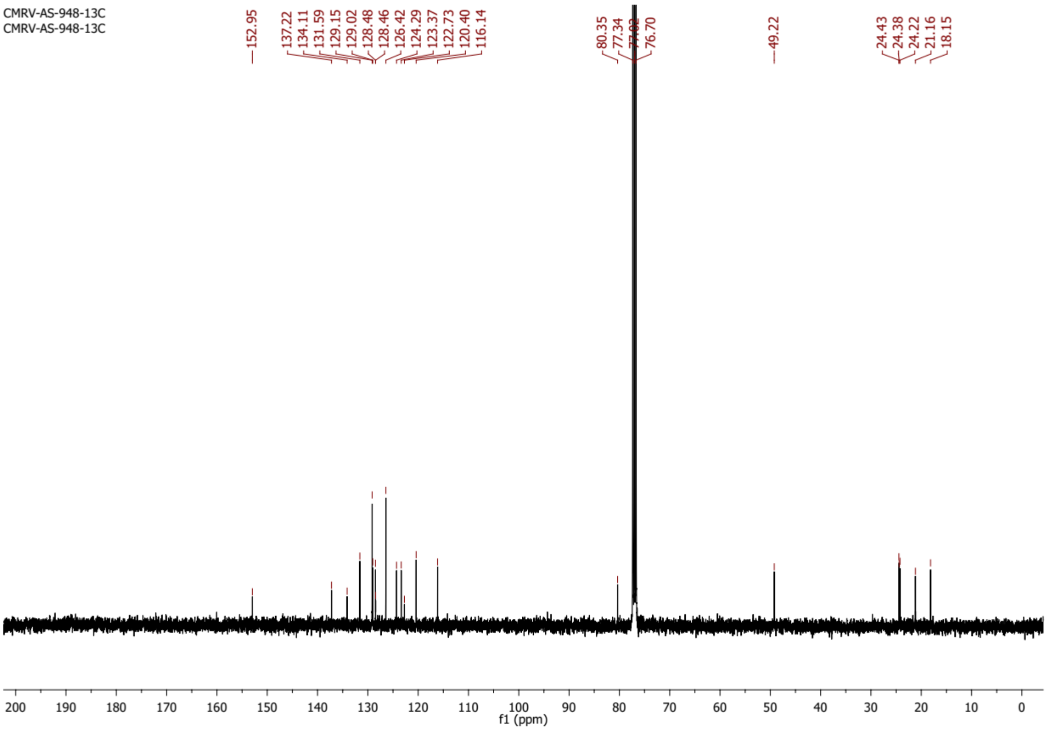
CMRV-AS-948-13C
CMRV-AS-948-13C
152.95
137.22 134.11 131.59 129.15 129.02 128.48 128.46 126.42 124.29 123.37 122.73 120.40 116.14
80.35 77.34 76.70
49.22
24.43 24.38 24.22 21.16 18.15
200 190 180 170 160 150 140 130 120 110 100 90 80 70 60 50 40 30 20 10 0
f1 (ppm)

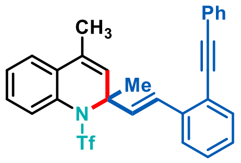
CH3
Me
Ph
N
Tf

13, 75%

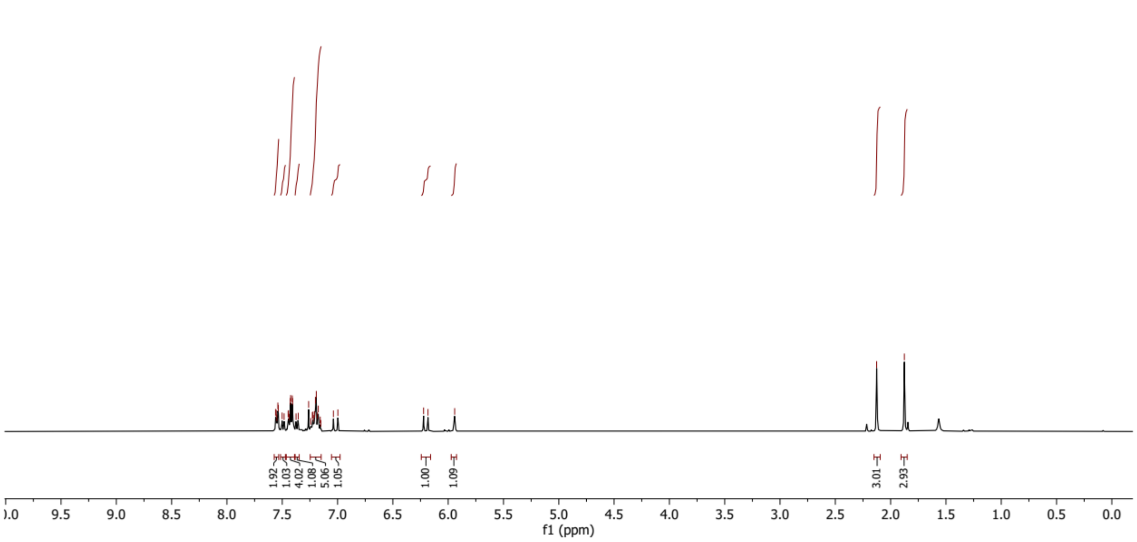
cmrv-as-923a-1h.1.fid
cmrv-as-923a-1h
7.44
7.43
7.42
7.41
7.41
7.38
7.36
7.26
7.23
7.22
7.21
7.20
7.19
7.18
7.17
7.04
6.22
6.18
5.94
2.13
1.88
1.92
1.03
4.02
1.08
5.06
1.05
1.00
1.09
3.01
2.93
f1 (ppm)

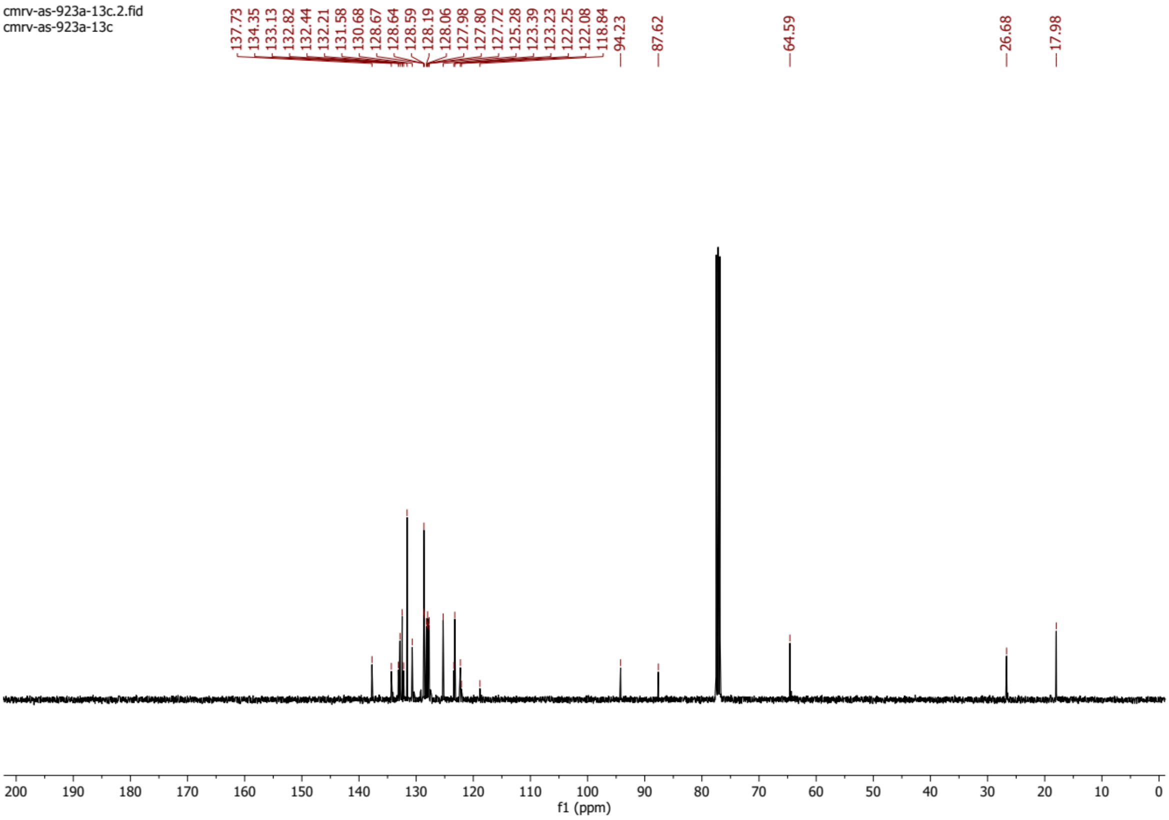
cmrv-as-923a-13c.2.fid
cmrv-as-923a-13c
137.73
134.35
133.13
132.82
132.44
132.21
131.58
130.68
128.67
128.64
128.59
128.19
128.06
127.98
127.80
127.72
125.28
123.39
123.23
122.25
122.08
118.84
94.23
87.62
64.59
26.68
17.98
f1 (ppm)

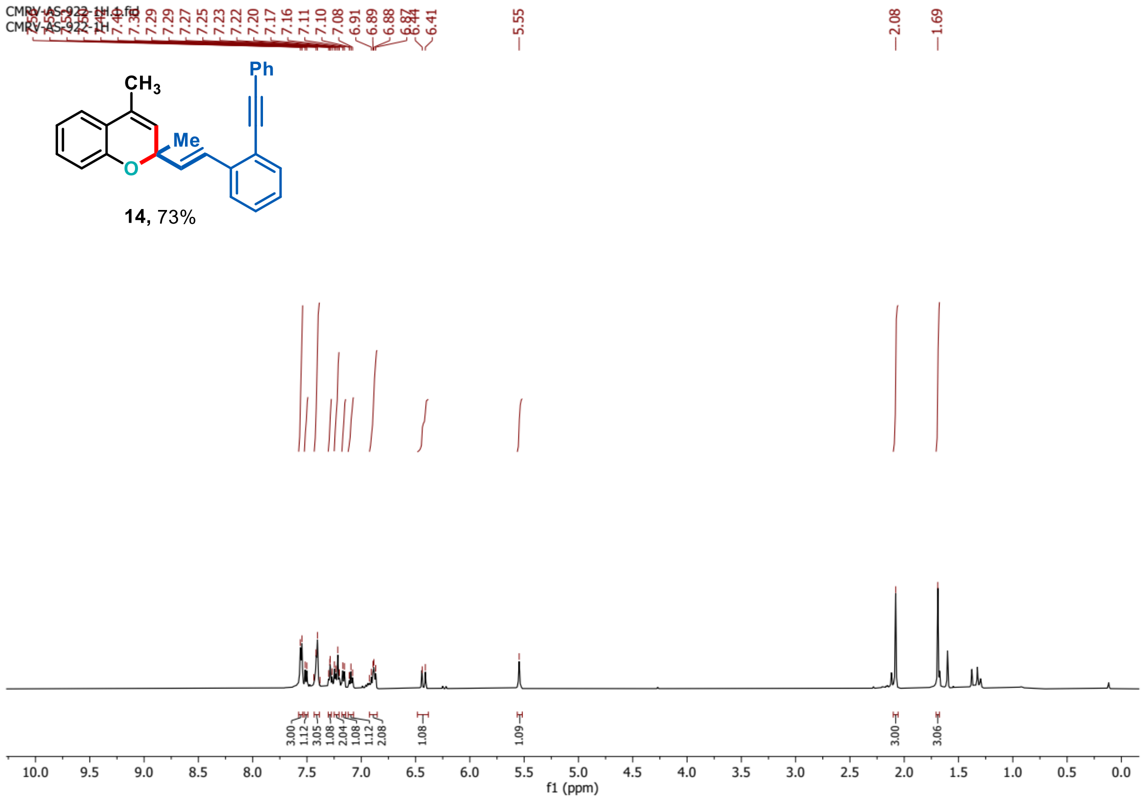
CMRV-AS-922-1H.1.fid
CMRV-AS-922-1H
7.29
7.29
7.27
7.25
7.23
7.22
7.20
7.17
7.16
7.11
7.10
7.08
6.91
6.89
6.88
6.87
6.44
6.41
5.55
2.08
1.69
CH3
Ph
Me
O
14, 73%
3.00
1.12
3.05
1.08
2.04
1.08
1.12
2.08
1.08
1.09
3.00
3.06
f1 (ppm)

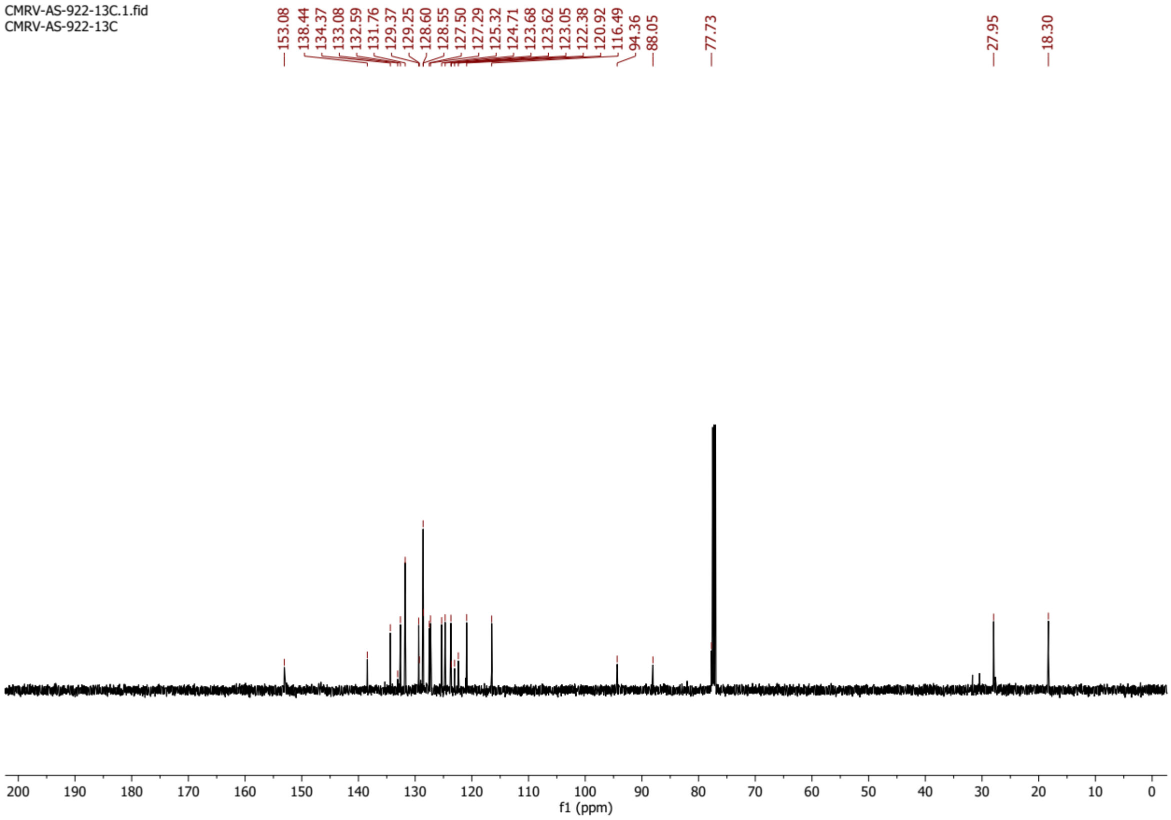
CMRV-AS-922-13C.1.fid
CMRV-AS-922-13C
153.08
138.44
134.37
133.08
132.59
131.76
129.37
129.25
128.60
128.55
127.50
127.29
125.32
124.71
123.68
123.62
123.05
122.38
120.92
116.49
94.36
88.05
77.73
27.95
18.30
f1 (ppm)
